# Global burden of hypertensive heart disease attributable to high body mass index from 1990 to 2021: a multidimensional analysis and public health response

**DOI:** 10.3389/fcvm.2025.1570390

**Published:** 2025-08-12

**Authors:** Zhenhai Sun, Rui Zhang, Mingyang Cong, Menghe Zhang, Tailong Lv, Huidan Xie, Shouqiang Chen

**Affiliations:** ^1^ Second School of Clinical Medicine, Shandong University of Traditional Chinese Medicine, Jinan, China; ^2^ Department of Acupuncture and Tuina, Dongying Shengli Oilfield Central Hospital, Dongying, China; ^3^ Department of Orthopedic Rehabilitation, Dongying Shengli Oilfield Central Hospital, Dongying, China; ^4^ Department of Cardiology, The Second Affiliated Hospital of Shandong University of Traditional Chinese Medicine, Jinan, China

**Keywords:** high body mass index, hypertensive heart disease, disability-adjusted life years, mortality, global burden

## Abstract

**Background:**

As the global population of obese individuals surpasses 878 million, the impact of high body mass index (BMI) on hypertensive heart disease (HHD) has risen to the third position among all diseases. However, the specific contribution of high BMI to the burden of HHD remains unclear.

**Methods:**

Data on deaths, disability-adjusted life years (DALYs), and their age-standardized rates (ASR) were obtained from the Global Burden of Disease (GBD) database. Population attributable fractions (PAF) was used to assess the contribution of risk factors. Various analytical methods, including decomposition analysis, cluster analysis, frontier analysis, age-period-cohort (APC) analysis, and Bayesian age-period-cohort (BAPC) analysis, were employed to investigate changes in disease burden.

**Results:**

The results showed an increasing global burden of HHD due to high BMI, with both mortality and DALYs doubling over the past 30 years. Their ASR also continued to rise. By 2021, the PAF for deaths and DALYs reached 44% and 49%, respectively. Population growth and aging were significant contributors to this disease burden. Low- and middle- Socio-Demographic Index (SDI) regions experienced the highest burden, particularly in East Asia, South Asia, North Africa and Middle East. Although the disease burden was lower in high-income areas, the increase was notable, especially in North America. Women and older populations faced higher risks, particularly alarming is the rapid increase in risk among younger populations in high SDI regions.

**Conclusion:**

HHD resulting from high BMI poses a significant global public health challenge, particularly in regions with middle and low SDI. While the heightened risk among women and older individuals has garnered considerable attention, the increasing risk among younger populations also necessitates greater focus. Targeted interventions should prioritize diet, exercise, medical security, and health education, with particular emphasis on enhancing policy support for low-income and high-risk groups. Future policies must integrate the social, economic, and cultural contexts of each region, implement comprehensive prevention and control strategies, and establish a multi-dimensional health promotion system.

## 1 Introduction

Hypertension is defined as a systolic blood pressure ≥140 mmHg and/or diastolic blood pressure ≥90 mmHg (based on the average of at least two measurements in a clinical resting state), and it is a significant risk factor for cardiovascular diseases (1). According to international guidelines, lifestyle interventions should be initiated immediately upon reaching this threshold. For individuals with high-risk conditions (such as diabetes) or those whose blood pressure remains at ≥140/90 mmHg, pharmacological treatment is recommended (1). Long-term uncontrolled hypertension can cause damage to the heart, leading to hypertensive heart disease (HHD). This condition involves abnormalities in cardiac structure and function, such as left ventricular hypertrophy, systolic and diastolic dysfunction, and arrhythmias (2). It can gradually progress to severe complications, including coronary artery disease, stroke, heart failure, and even sudden cardiac death (2). Importantly, HHD accounts for approximately one-quarter of global heart failure cases and has become the fourth leading cause of death from cardiovascular diseases (3, 4). Recent studies have estimated that approximately 18.6 million people globally suffer from HHD, with a disability-adjusted life years (DALYs, a measure that combines the years of life lost due to premature death and the years lived with disability) burden of 21.51 million and a death toll of 1.16 million, which is projected to rise to 1.57 million by 2034 (5, 6). These burdens have significant economic implications. In the United States, the economic impact of hypertension is over $11 billion annually in lost productivity (7). Additionally, the cost of cardiovascular events directly associated with hypertension in Europe is estimated to reach €51.3 billion (8).

Concurrently, the issue of obesity has surged to pandemic levels, with the global obesity rate rising from 6.1% to 16% over the past few decades, now affecting 878 million people (9). At present, Obesity has become a key driver of HHD through pathological changes such as metabolic disorders, insulin resistance, and chronic inflammation (10). It is concerning that the impact of high body mass index (BMI) on HHD has escalated from fifth place in terms of risk factors in 2017 to third place in 2021, surpassing its influence on the burden of stroke and chronic kidney disease (11, 12). Critically, as the global prevalence of high BMI continues to rise, the disease burden associated with HHD is anticipated to increase further.

Although previous studies have shown that, with the increasing number of individuals with high BMI globally, the contribution of high BMI to HHD has gradually become dominant, this relationship has not yet been fully quantified (12). Current research primarily focuses on the overall disease burden of HHD and health inequalities, but the disease burden of HHD attributable to high BMI has not been systematically explored (13, 14). Therefore, this study aims to fill this gap by assessing the impact of high BMI on the burden of HHD in different regions, genders, and age groups, based on the Global Burden of Disease (GBD) database, and providing data to support public health interventions considering socioeconomic, cultural, and environmental factors.

## 2 Materials and methods

### 2.1 Data sources

The GBD 2021 provides a comprehensive evaluation of epidemiological data regarding the health effects associated with 371 diseases, injuries, or disabilities, as well as 88 risk factors, across 21 GBD regions and 204 countries worldwide. All data utilized in this study are sourced from the Global Health Data Exchange. HHD is classified according to International Classification of Diseases (ICD) codes: ICD-9 codes 402-402.9 and ICD-10 codes I11-I11.9 (15). High BMI is defined as a BMI of 25 kg/m^2^ or greater (16). We obtained global data on high-BMI-related HHD from 1990 to 2021, which encompasses information on deaths, DALYs, population attributable fraction (PAF, a measure that quantifies the proportion of disease burden attributable to a specific risk factor), as well as pertinent age, gender, and geographic location data.

### 2.2 The social demographic index

The Social Demographic Index (SDI) is an indicator that combines factors such as per capita income, education level, and total fertility rate. It reflects the levels of social and demographic development across different regions. Based on the SDI value, we categorized countries into five categories: Low, Low-Middle, Middle, High-Middle, and High (17).

### 2.3 Estimates of the global burden and its trends

Deaths and DALYs are utilized to characterize disease burden in two quantitative forms: the number of cases and the age-standardized rate (ASR). The latter allows for cross-regional comparisons by adjusting for the age structure of the population and reports a 95% uncertainty interval (UI) (18). To attribute the burden to high BMI, we calculated the PAF and its 95% UI (19).

Additionally, we assessed temporal trend patterns in disease burden by calculating the estimated annual percentage change (EAPC, a measure that quantifies the yearly rate of change in health indicators over time) with 95% confidence interval (CI) (20). We also employed hierarchical cluster analysis to identify groups exhibiting similar trends based on EAPC values, thereby evaluating the changing patterns of disease burden across 21 GBD regions and 204 countries.

### 2.4 Decomposition analysis

Decomposition analysis was used to examine the changes in DALYs and deaths by considering three primary factors: aging, population growth, and epidemiological changes. This approach aims to elucidate the specific contribution of each factor to the overall change (21). A positive value indicates an increase in disease burden driven by certain factors (e.g., an increase in case numbers due to population growth). A negative value signifies a decrease in disease burden attributable to certain factors (e.g., a reduction in disease incidence due to improvements in healthcare).

### 2.5 Frontier analysis

We employed frontier analysis to evaluate the relative efficiency of various regions or countries in managing the burden of HHD linked to high BMI (22). This facilitates the identification of the best-performing regions, highlights opportunities for optimizing resource allocation, and emphasizes the potential for burden reduction.

### 2.6 Age-period-cohort model

This study employed the age-period-cohort (APC) model to dynamically analyze the changing trends in deaths and DALYs, both globally and within each SDI region. This analysis was based on various temporal dimensions, including age, period, and birth cohort. Further details regarding this methodology have been discussed in the existing literature (23).

### 2.7 Prediction model

We employed the Bayesian age-period-cohort (BAPC) model in conjunction with an ensemble nested Laplacian approximation to forecast sex- and age-stratified deaths and DALYs from 2022 to 2036 (24). In order to verify the robustness and sensitivity of the predicted trends, we applied the Autoregressive Integrated Moving Average (ARIMA) model at both the global level and by gender. This model, based on time series analysis methods, provides independent cross-validation for the BAPC results (25).

### 2.8 Statistical analysis

We utilized deaths, DALYs, Age-Standardized Mortality Rate (ASMR), Age-Standardized Disability-Adjusted Life Year Rate (ASDR), along with associated EAPC and PAF, to compare the disease burden and temporal trends across sex, age, and geographic regions. ASMR and ASDR adjust for the differences in age structure of deaths and DALYs, respectively, enabling cross-population comparisons. We further explored the relationship between ASDR, ASMR, and SDI. Decomposition and frontier analyses were applied to evaluate the factors driving the increase in disease burden and potential areas for improvement. The APC model was used to supplement the analysis of the impact of different time dimensions on disease burden. Finally, the BAPC model was used to predict the trends in the disease burden of high BMI-related HHD by 2036. All data processing, analysis, and graphical outputs were performed using R software 4.2.3.

## 3 Results

### 3.1 PAF of high-BMI related HHD by regional and age groups

**Figure 1:**
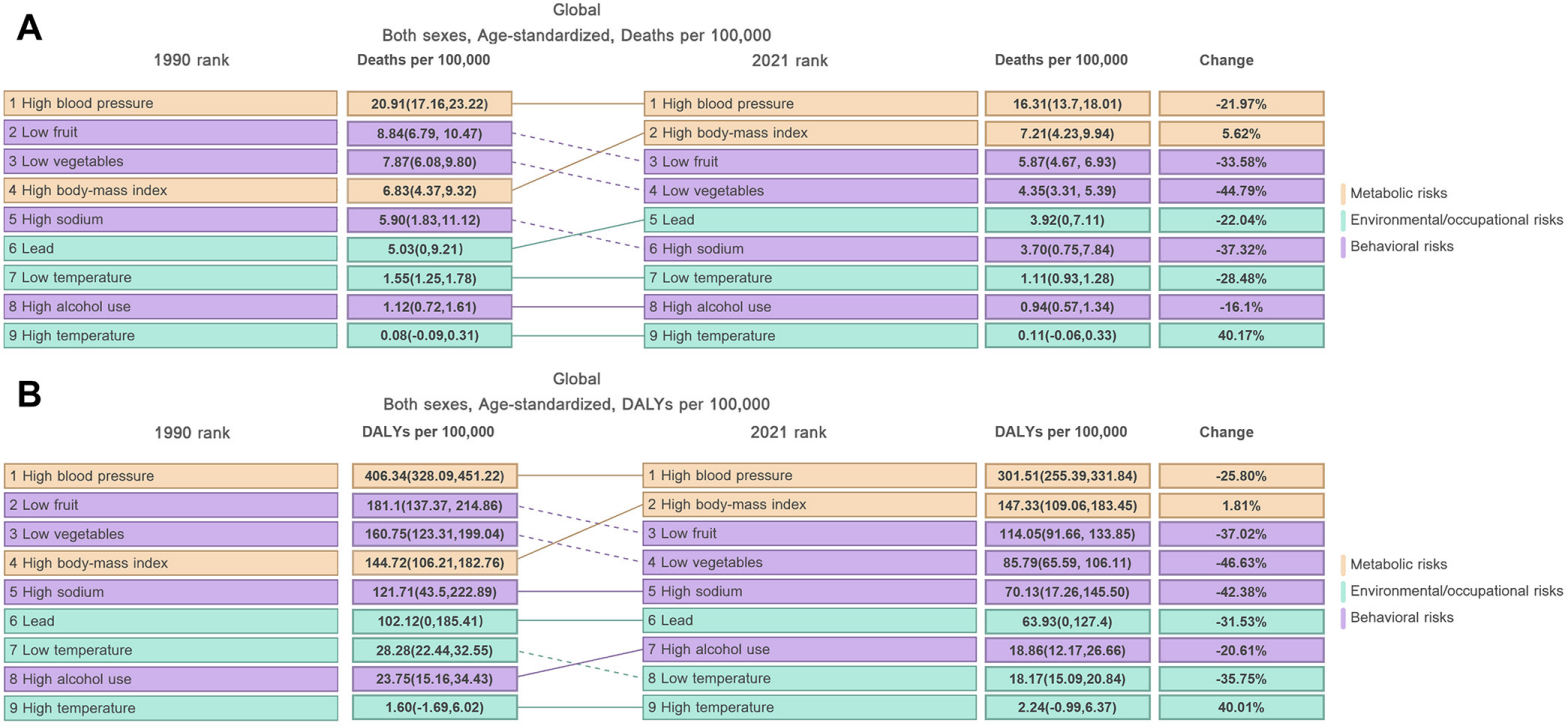
Temporal trends in the relative contribution of various risk factors to HHD from 1990 to 2021: **(A)** ASMR, **(B)** ASDR. Risk categories are color-coded: Metabolic risks (orange), Environmental/occupational risks (green), and Behavioral risks (purple).

**Figure 2:**
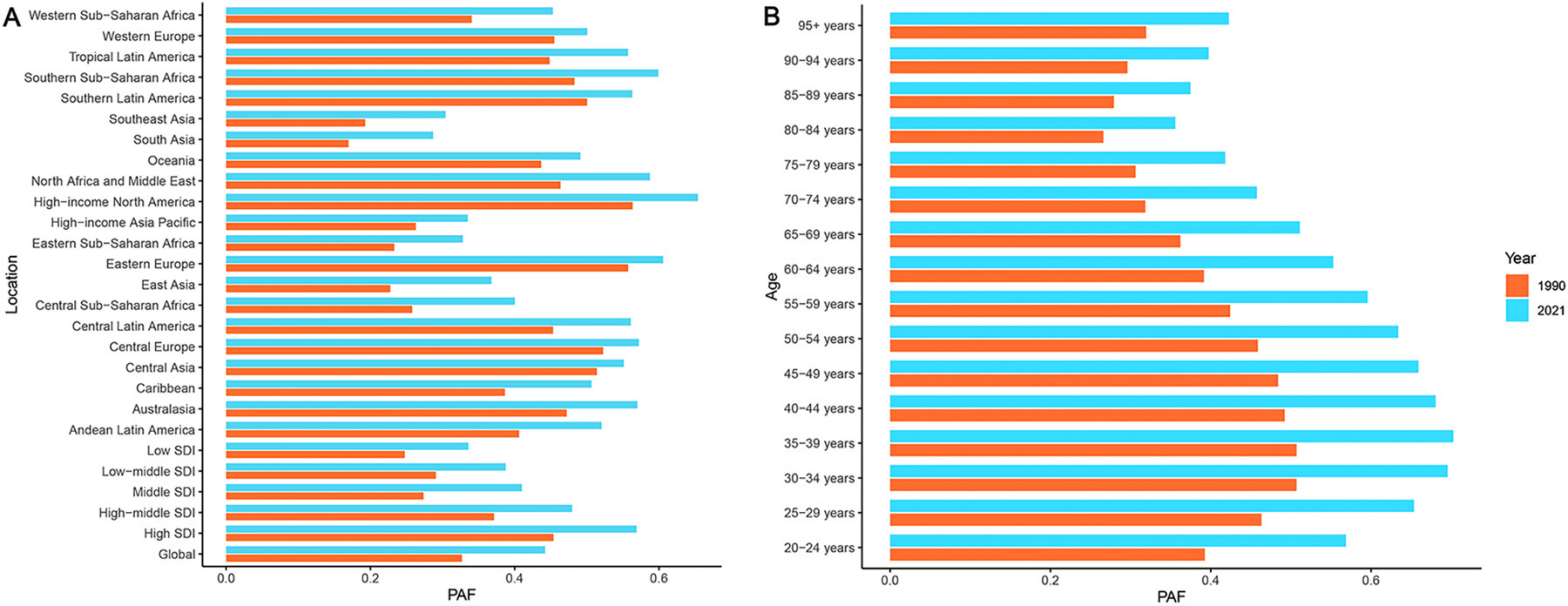
Changes in the PAF of deaths for HHD attributable to high BMI from 1990 to 2021: **(A)** Location, **(B)** Age group. Orange represents 1990, blue represents 2021.

From 1990 to 2021, high BMI rose from the fourth to the second position, becoming the most significant risk factor for HHD, second only to hypertension in terms of mortality and disability burden (Figure 1). Specifically, the PAF for global deaths increased significantly from 33% in 1990 to 44% in 2021, while the PAF for DALYs reached 49% by 2021 (Figure 2; Supplementary Table S1). Regionally, high-income North America had the highest PAF for deaths, reaching 65% (Supplementary Table S1). Across all age groups, the PAF for deaths from HHD increased significantly over the past 30 years. In 2021, the PAF for the 20–69 age group surpassed 50%, with the 35–39 age group exhibiting the highest PAF at 70% (Supplementary Table S2). The PAF for DALYs demonstrated a similar trend to that of the PAF for deaths (Supplementary Figure S1). This indicates that the younger working-age population is facing increasingly severe challenges.

### 3.2 Global burden of high BMI-related HHD

**Figure 3:**
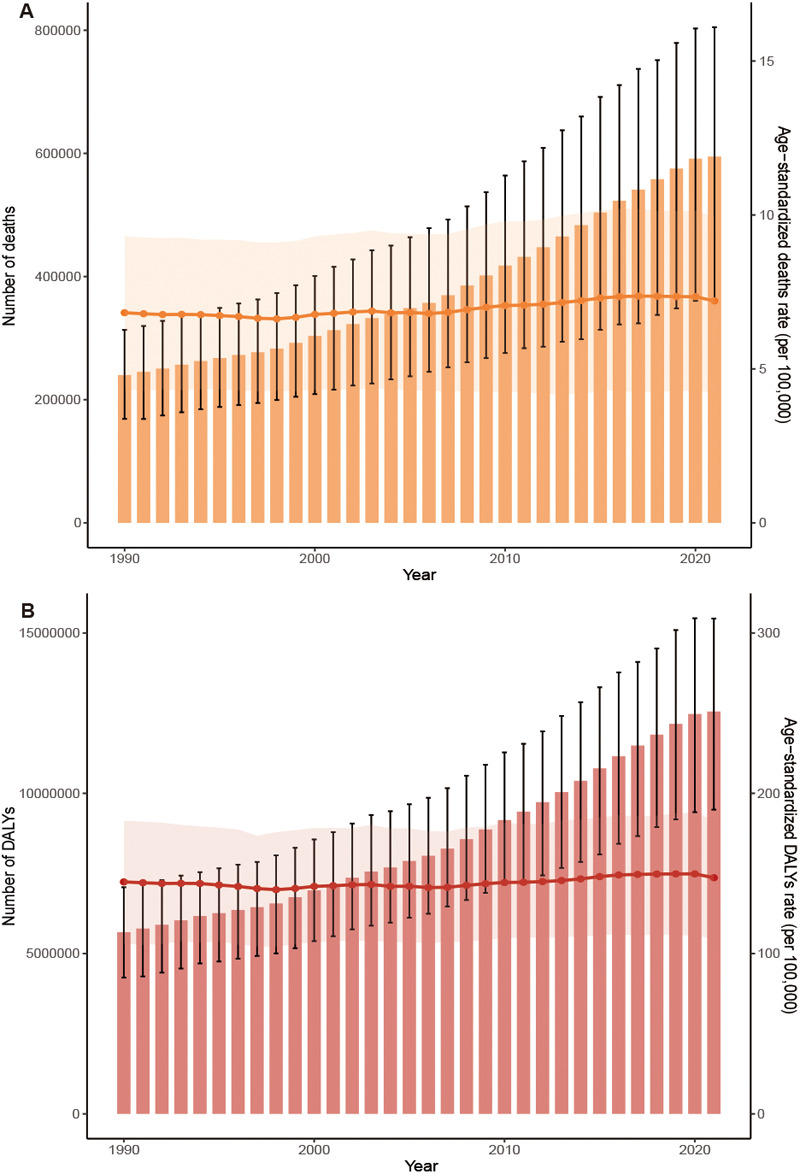
Trends in the global burden of HHD attributable to high BMI from 1990 to 2021: **(A)** deaths and ASMR, **(B)** DALYs and ASDR.

Tables 1, 2 present the global disease burden of HHD attributable to high BMI for the years 1990 and 2021, respectively. The data show a significant increasing trend in the absolute disease burden from 1990 to 2021, with global deaths rising by approximately 148% and DALYs increasing by about 122%. However, both ASMR and ASDR remained relatively stable overall, showing only a slight increasing trend, with EAPCs of 0.33 (95% CI: 0.27–0.39) and 0.15 (95% CI: 0.10–0.21), respectively (Tables 1, 2; Figure 3).

**Table 1:** Deaths and ASMR of high BMI-related HHD in 1990 and 2021, with the temporal trends from 1990 to 2021, by global, sex, SDI and GBD region.

Deaths	1990		2021		1990–2021
Location	Deaths cases No. (95% UI)	ASR per 100,000 No. (95% UI)	Deaths cases No. (95% UI)	ASR per 100,000 No. (95% UI)	EAPC No. (95% CI)
Global	240,096 (168,851–313,374)	6.83 (4.37–9.32)	594,899 (362,924–804,914)	7.21 (4.23–9.94)	0.33 (0.27–0.39)
Sex					
Male	93,613 (66,086–120,398)	5.97 (3.74–8.25)	243,060 (159,715–323,101)	6.69 (3.98–9.3)	0.55 (0.47–0.63)
Female	146,483 (97,562–196,210)	7.37 (4.6–10.12)	351,839 (201,116–494,197)	7.53 (4.36–10.53)	0.2 (0.15–0.25)
SDI					
Low SDI	19,835 (11,275–27,741)	10.04 (5.58–14.81)	49,041 (31,965–64,989)	11.27 (6.94–15.88)	0.3 (0.22–0.39)
Low-middle SDI	41,406 (28,211–55,455)	8.11 (5.03–11.49)	113,743 (81,654–147,945)	8.96 (5.9–12.38)	0.43 (0.39–0.47)
Middle SDI	80,183 (52,533–106,376)	9.61 (5.53–13.78)	196,805 (122,494–276,061)	8.3 (4.53–12.23)	−0.44 (−0.63 to −0.26)
High-middle SDI	55,916 (37,393–74,692)	6.62 (3.92–9.36)	132,675 (67,107–191,444)	7 (3.38–10.17)	0.44 (0.26–0.62)
High SDI	42,339 (25,102–57,793)	3.88 (2.28–5.34)	101,715 (51,587–144,018)	4.38 (2.59–5.87)	0.83 (0.69–0.98)
GBD region					
East Asia	60,550 (35,297–84,646)	9.53 (4.68–14.49)	129,831 (67,188–204,255)	6.89 (3.18–11.52)	−1.11 (−1.52 to −0.69)
Oceania	311 (201–434)	11.36 (7.24–16.17)	683 (479–996)	9.27 (6.25–13.18)	−0.75 (−0.81 to −0.7)
Southeast Asia	13,602 (8,334–18,952)	5.7 (3.36–8.2)	43,048 (29,946–57,024)	6.97 (4.62–9.6)	0.69 (0.63–0.75)
Central Asia	3,545 (2,503–4,646)	8.24 (5.49–11.12)	7,827 (5,329–10,301)	11.36 (7.06–15.74)	1.39 (0.78–2.01)
Central Europe	16,693 (11,459–22,125)	12.18 (7.52–16.66)	34,417 (18,115–47,345)	14.52 (7.94–19.75)	1.11 (0.85–1.37)
Southern Latin America	3,502 (2,278–4,659)	8.15 (4.96–11.09)	7,116 (3,480–10,123)	7.75 (3.88–10.95)	0.21 (0.05–0.36)
Eastern Europe	6,652 (5,127–8,086)	2.5 (1.86–3.13)	15,863 (10,102–21,120)	4.49 (2.96–5.91)	1.92 (1–2.85)
High-income Asia Pacific	4,662 (2,307–7,154)	2.77 (1.18–4.44)	6,724 (2,088–11,979)	1 (0.42–1.66)	−2.93 (−3.67 to −2.19)
Western Europe	23,441 (11,486–34,450)	3.94 (1.93–5.81)	52,473 (16,490–84,212)	4.11 (1.5–6.37)	0.72 (0.54–0.91)
Australasia	356 (204–509)	1.63 (0.85–2.38)	838 (337–1,224)	1.34 (0.62–1.9)	−0.47 (−0.87 to −0.07)
High-income North America	13,621 (9,177–17,789)	3.9 (2.73–5.02)	44,695 (28,218–58,029)	6.81 (4.69–8.59)	2.04 (1.89–2.2)
Caribbean	1,896 (1,366–2,466)	7.81 (5.33–10.44)	5,459 (3,576–7,226)	9.98 (6.59–13.15)	1.29 (1.07–1.52)
Andean Latin America	1,094 (742–1,481)	5.86 (3.68–8.28)	2,475 (1,383–3,575)	4.38 (2.37–6.42)	−0.33 (−0.74 to 0.09)
Central Latin America	5,929 (4,039–7,927)	8.45 (5.15–11.79)	12,767 (7,671–17,935)	5.4 (3.14–7.69)	−1.55 (−1.76 to −1.34)
Tropical Latin America	8,417 (6,357–10,542)	10.36 (7.02–13.75)	17,129 (10,978–22,648)	6.9 (4.29–9.26)	−1.15 (−1.3 to −1.01)
North Africa and Middle East	35,436 (24,345–47,300)	26.35 (15.88–36.47)	85,253 (57,659–110,088)	23.2 (14.02–31.44)	−0.29 (−0.4 to −0.18)
South Asia	14,746 (8,200–21,658)	3.03 (1.56–4.88)	61,433 (40,106–90,559)	4.73 (2.81–7.34)	1.58 (1.52–1.64)
Southern Sub-Saharan Africa	4,449 (3,187–5,890)	18.43 (11.7–25.51)	13,652 (9,929–17,284)	28.4 (17.52–37.54)	1.56 (1.1–2.01)
Eastern Sub-Saharan Africa	8,702 (4,742–12,349)	13.61 (7.08–19.8)	19,481 (11,561–27,470)	13.95 (7.65–21.25)	−0.04 (−0.13 to 0.05)
Central Sub-Saharan Africa	3,259 (1,621–4,943)	17.42 (8.38–27.05)	11,187 (6,476–17,053)	26.46 (14.82–40.53)	1.32 (1.28–1.35)
Western Sub-Saharan Africa	9,233 (6,019–12,929)	11.76 (7.05–17.59)	22,547 (13,463–30,630)	13.03 (7.79–18.62)	0.1 (−0.05 to 0.26)

**Table 2:** DALYs and ASDR of high BMI-related HHD in 1990 and 2021, with the temporal trends from 1990 to 2021, by global, sex, SDI and GBD region.

DALYs	1990		2021		1990–2021
Location	DALYs cases No. (95% UI)	ASR per 100,000 No. (95% UI)	DALYs cases No. (95% UI)	ASR per 100,000 No. (95% UI)	EAPC No. (95% CI)
Global	5,666,017 (4,251,059–7,069,738)	144.72 (106.21–182.76)	12,551,752 (9,489,124–15,451,016)	147.33 (109.06–183.45)	0.15 (0.1–0.21)
Sex					
Male	2,414,649 (1,794,252–3,011,391)	131.37 (96.12–166.66)	5,646,140 (4,247,052–6,976,635)	142.25 (104.32–179.41)	0.4 (0.34–0.47)
Female	3,251,367 (2,294,581–4,161,209)	154.37 (107.15–198.32)	6,905,612 (5,009,097–8,756,181)	150.26 (109.6–190.15)	−0.04 (−0.09 to 0.01)
SDI					
Low SDI	550,419 (306,312–765,530)	233.39 (132.99–326.55)	1,318,232 (895,030–1,781,084)	249.65 (164.84–328.72)	0.1 (0.02–0.18)
Low-middle SDI	1,064,622 (740,286–1,387,682)	174.5 (122.01–231.14)	2,774,973 (2,163,092–3,408,019)	192.73 (147.45–244.13)	0.39 (0.37–0.41)
Middle SDI	1,979,473 (1,355,214–2,590,232)	197.12 (132.36–261.86)	4,255,947 (3,088,318–5,431,403)	163.33 (112.87–214.45)	−0.6 (−0.77 to −0.42)
High-middle SDI	1,203,848 (903,408–1,536,884)	127.41 (92.84–165.58)	2,296,315 (1,583,465–3,023,135)	118.94 (81.23–157.67)	−0.11 (−0.29 to 0.06)
High SDI	858,426 (643,921–1,067,628)	79.52 (59.95–98.57)	1,888,250 (1,342,869–2,367,629)	97.25 (76.46–115.81)	1.16 (0.99–1.33)
GBD region					
East Asia	1,430,316 (849,323–1,974,447)	180.32 (105.12–256.67)	2,468,556 (1,553,650–3,558,544)	120.29 (72.71–178.97)	−1.38 (−1.81 to −0.96)
Oceania	9,812 (6,241–13,922)	290.91 (188.24–402.99)	21,673 (14,664–31,794)	243.69 (171.23–353.9)	−0.65 (−0.69 to −0.61)
Southeast Asia	388,646 (235,386–525,913)	141.2 (86.26–193.98)	1,159,653 (809,395–1,490,326)	168.45 (118.2–217.74)	0.61 (0.55–0.67)
Central Europe	341,701 (266,977–420,820)	234.6 (179.04–292.21)	576,781 (392,431–742,466)	256.77 (182.69–324.76)	0.79 (0.55–1.04)
Central Asia	85,508 (68,292–103,807)	182.97 (142.27–227)	177,446 (135,756–223,717)	225.17 (167.21–286.4)	0.73 (0.11–1.36)
Eastern Europe	168,680 (139,917–195,265)	61.1 (50.73–70.84)	312,736 (239,270–385,024)	90.73 (71.47–110.51)	1.05 (0.11–1.99)
High-income Asia Pacific	87,925 (58,624–119,645)	47.35 (29.06–66.74)	93,541 (44,903–149,938)	18.25 (11.86–25.8)	−2.84 (−3.51 to −2.16)
Australasia	6,699 (4,801–8,677)	29.57 (20.8–38.89)	13,457 (8,334–17,818)	24.32 (16.24–31)	−0.52 (−0.95 to −0.09)
Western Europe	390,360 (248,712–526,128)	66.72 (44.26–88.38)	667,214 (285,609–993,373)	59.46 (31.29–83.16)	0.23 (0.06–0.39)
Southern Latin America	71,894 (55,357–88,611)	158.65 (118.36–198.15)	115,625 (75,591–149,422)	129.88 (86.97–166.68)	−0.34 (−0.47 to −0.2)
High-income North America	318,549 (254,229–382,521)	95.99 (77.8–113.6)	983,533 (778,733–1,178,286)	172.21 (142.82–201.13)	2.31 (2.17–2.46)
Caribbean	47,198 (36,942–58,825)	180.52 (138.74–228.31)	124,033 (94,913–153,885)	230.58 (176.69–285.28)	1.2 (1.01–1.4)
Andean Latin America	26,361 (20,151–32,833)	125.44 (93.2–159.74)	51,491 (36,479–68,298)	87.42 (60.88–117.12)	−0.59 (−1.01 to −0.15)
Central Latin America	130,912 (103,854–159,737)	163.52 (120.71–206.61)	248,377 (179,783–324,940)	101.12 (71.3–132.94)	−1.7 (−1.94 to −1.45)
North Africa and Middle East	845,482 (596,104–1,089,020)	522.42 (363.93–686.68)	1,910,275 (1,455,255–2,382,733)	440.2 (317.57–558.72)	−0.49 (−0.57 to −0.4)
Tropical Latin America	216,908 (178,699–256,744)	231.5 (183.38–281.97)	366,921 (286,760–442,702)	143.42 (110.18–174.44)	−1.49 (−1.63 to −1.36)
Central Sub-Saharan Africa	89,500 (45,608–137,883)	390.74 (195.43–580.93)	291,995 (171,821–431,335)	546.75 (322.13–825.58)	1.03 (0.99–1.07)
South Asia	399,107 (230,380–577,004)	67.92 (38.4–98.92)	1,490,183 (1,049,058–2,077,926)	101.32 (70.52–143.72)	1.41 (1.37–1.45)
Eastern Sub-Saharan Africa	244,965 (134,295–342,482)	311.7 (169.9–439.48)	521,322 (338,086–709,150)	298.05 (180.48–414.03)	−0.32 (−0.42 to −0.22)
Southern Sub-Saharan Africa	116,312 (95,260–146,331)	412.08 (317.08–535.7)	328,748 (265,705–399,280)	578.12 (437.99–722.53)	1.27 (0.82–1.73)
Western Sub-Saharan Africa	249,180 (165,152–341,063)	272.01 (178–372.99)	628,194 (371,114–822,109)	296.3 (180.82–399.61)	0.07 (−0.09 to 0.23)

### 3.3 High BMI-related HHD burden stratified by SDI quintiles

**Figure 4:**
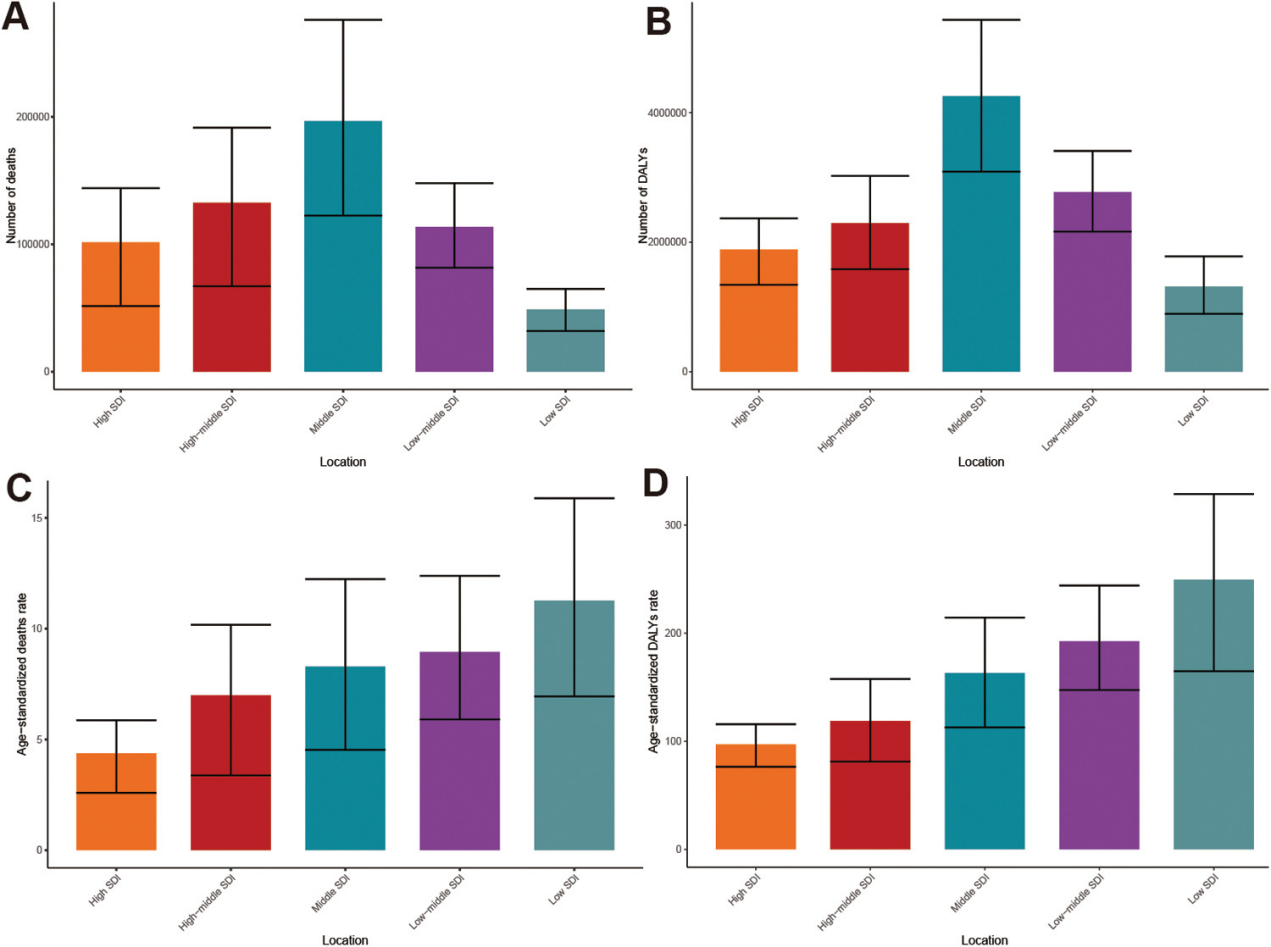
Burden of HHD attributable to high BMI in 2021 across different SDI quintiles: **(A)** deaths, **(B)** DALYs, **(C)** ASMR, **(D)** ASDR.

In 2021, the disease burden of HHD associated with high BMI exhibited significant variations across different SDI regions. The middle SDI region bore the heaviest absolute disease burden, recording the highest number of deaths at 196,805 (95% UI: 122,494–276,061) and DALYs at 4,255,947 (95% UI: 3,088,318–5,431,403) (Tables 1, 2; Figures 4A,B). Conversely, while the low SDI region reported the lowest deaths and DALYs, its ASMR and ASDR were the highest, at 11.27 (95% UI: 6.94–15.88) per 100,000 and 249.65 (95% UI: 164.84–328.72) per 100,000, respectively (Tables 1, 2; Figures 4C,D). In contrast, high SDI regions demonstrated the lowest ASMR and ASDR, recorded at 4.38 (95% UI: 2.59–5.87) per 100,000 and 97.25 (95% UI: 76.46–115.81) per 100,000, respectively (Tables 1, 2; Figures 4C,D).

In the 5 SDI regions, deaths and DALYs attributable to high BMI-related HHD exhibited an increasing trend from 1990 to 2021 (Supplementary Figure S2). The ASMR revealed only a slight decrease in the middle SDI region, while other SDI regions experienced an upward trend (Table 1). The ASDR in the middle SDI and high-middle SDI regions demonstrated a slight decline, respectively, whereas the remaining regions displayed an increasing trend (Table 2). Notably, the EAPC for both ASMR and ASDR was highest in high SDI regions, recorded at 0.83 (95% CI: 0.69–0.98) and 1.16 (95% CI: 0.99–1.33), respectively. Despite having the lowest number of deaths and DALYs, the low SDI regions maintained consistently high ASMR and ASDR throughout the period.

**Figure 5:**
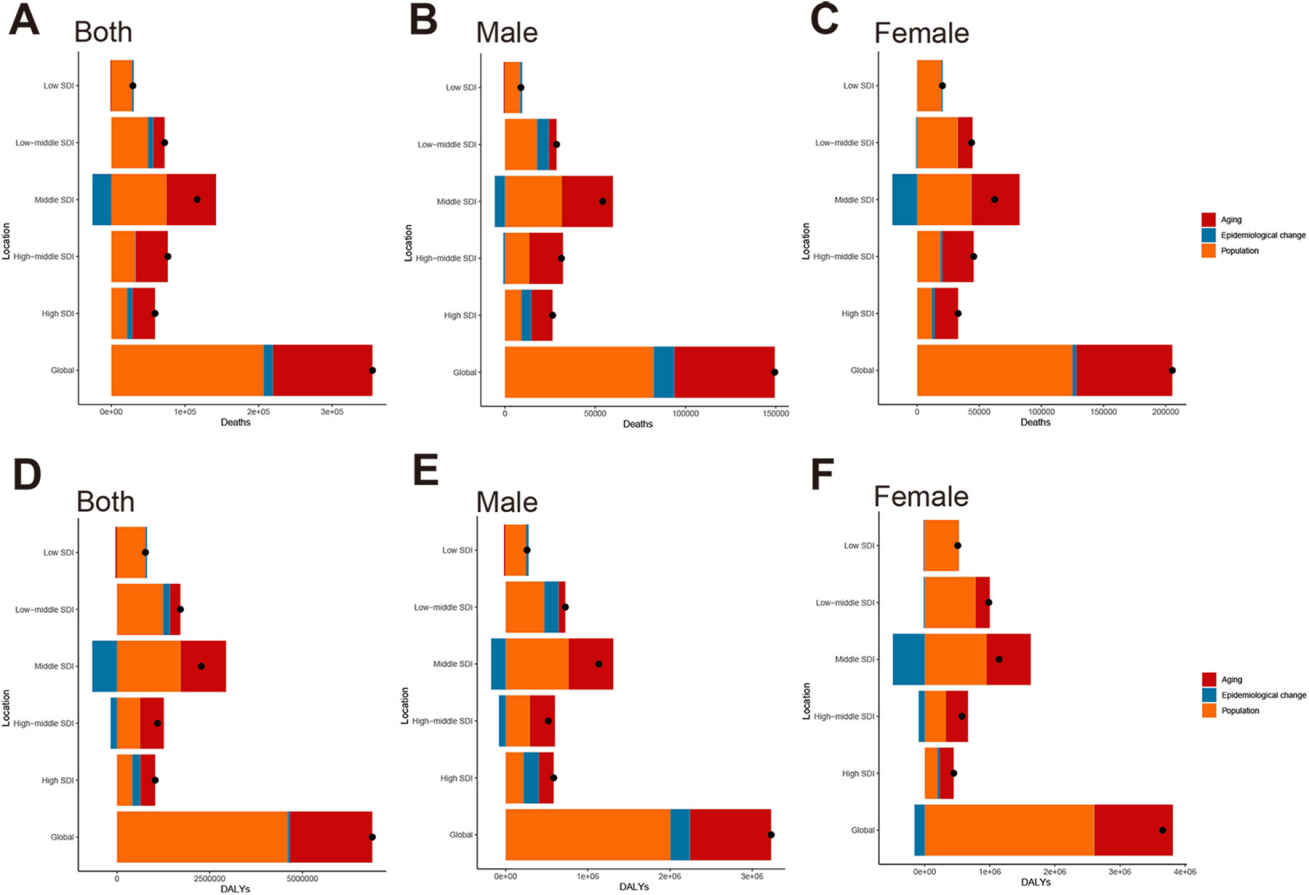
Decomposition analysis of global changes in deaths and DALYs for HHD attributable to high BMI from 1990 to 2021, stratified by SDI level and sex: **(A)** overall deaths, **(B)** deaths in males, **(C)** deaths in females; **(D)** overall DALYs, **(E)** DALYs in males, **(F)** DALYs in females.

Decomposition analysis revealed the driving factors behind the rising global burden. Population growth was the primary contributor, accounting for 58.41% of deaths and 66.87% of DALYs (Figure 5). Aging was the second largest factor, contributing 38.14% to the increase in mortality and 32.4% to the growth in DALYs (Figure 5). The impact of these factors varied significantly across different levels of the SDI. In low SDI regions, population growth was the main driver of disease increase, contributing 96.65% to deaths and 100.46% to DALYs. In contrast, in high SDI regions, the contributions of aging and population growth were nearly equal (Supplementary Table S3). An interesting phenomenon in middle SDI regions was that improvements in epidemiological changes helped alleviate the disease burden, offsetting some of the increases driven by population factors. This was likely related to the region's transition towards rapid economic development and enhanced healthcare standards. These beneficial changes led to a reduction in deaths by 21.97% and a decrease in DALYs by 29.22%. Additionally, global trends for men and women exhibit similarities (Figure 5).

### 3.4 High BMI-related HHD burden stratified by 21 GBD

**Figure 6:**
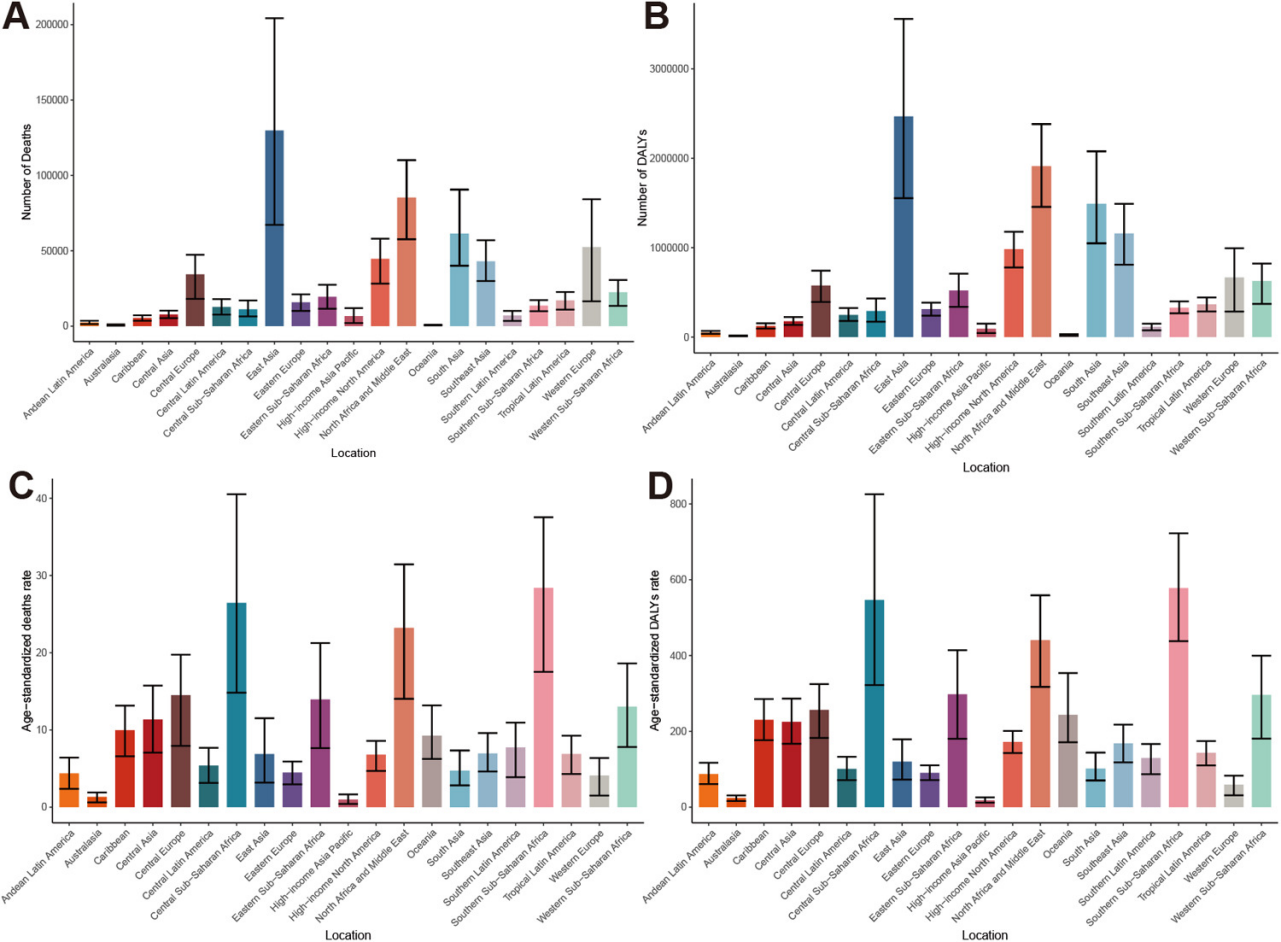
Global burden of HHD attributable to high BMI in 21 GBD regions in 2021: **(A)** deaths, **(B)** DALYs, **(C)** ASMR, **(D)** ASDR.

In 2021, there were significant disparities in the burden of HHD associated with high BMI across 21 GBD regions. East Asia carried the heaviest death burden with 129,831 (95% UI: 67,188–204,255). This was followed by North Africa and Middle East, and South Asia. A similar pattern was observed in the DALYs, with East Asia recording the highest values (Tables 1, 2; Figures 6A,B). ASR also revealed striking contrasts. Southern Sub-Saharan Africa exhibited the highest ASMR at 28.4 per 100,000 (95% UI: 17.52–37.54) and ASDR at 578.12 per 100,000 (95% UI: 437.99–722.53). This was followed by Central Sub-Saharan Africa, North Africa and Middle East, while High-Income Asia Pacific, Australasia, and Western Europe reported the lowest value (Tables 1, 2; Figures 6C,D).

**Figure 7:**
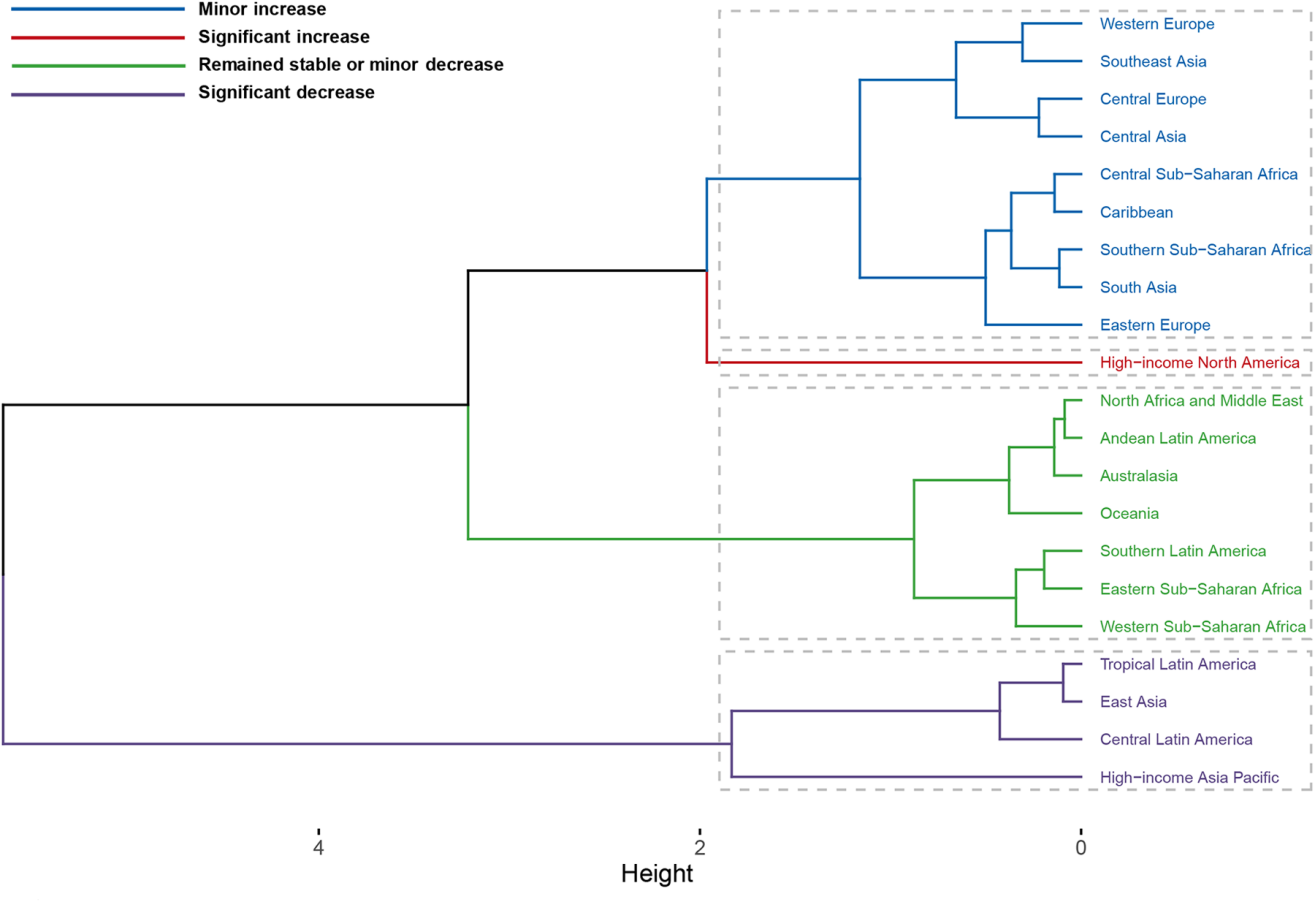
Comprehensive clustering of the EAPC for high BMI-related HHD across 21 GBD regions from 1990 to 2021.

Trend analysis from 1990 to 2021 revealed divergent patterns in disease burden changes across these regions. Comprehensive EAPC cluster analysis of ASMR and ASDR revealed that among the 21 GBD regions, 1 region experienced a significant increase, 9 regions showed a slight increase, 7 regions remained stable or exhibited a slight decrease, and 4 regions experienced a significant decrease (Figure 7). Notably, High-income North America recorded the largest increases in ASMR and ASDR, with EAPCs of 2.04 (95% CI: 1.89–2.20) and 2.31 (95% CI: 2.17–2.46), respectively. In contrast, High-income Asia Pacific showed the most substantial improvements in ASMR and ASDR, with EAPCs of −2.93 (95% CI: −3.67 to −2.19) and −2.84 (95% CI: −3.51 to −2.16), respectively (Tables 1, 2).

### 3.5 High BMI-related HHD burden stratified by 204 countries

**Figure 8:**
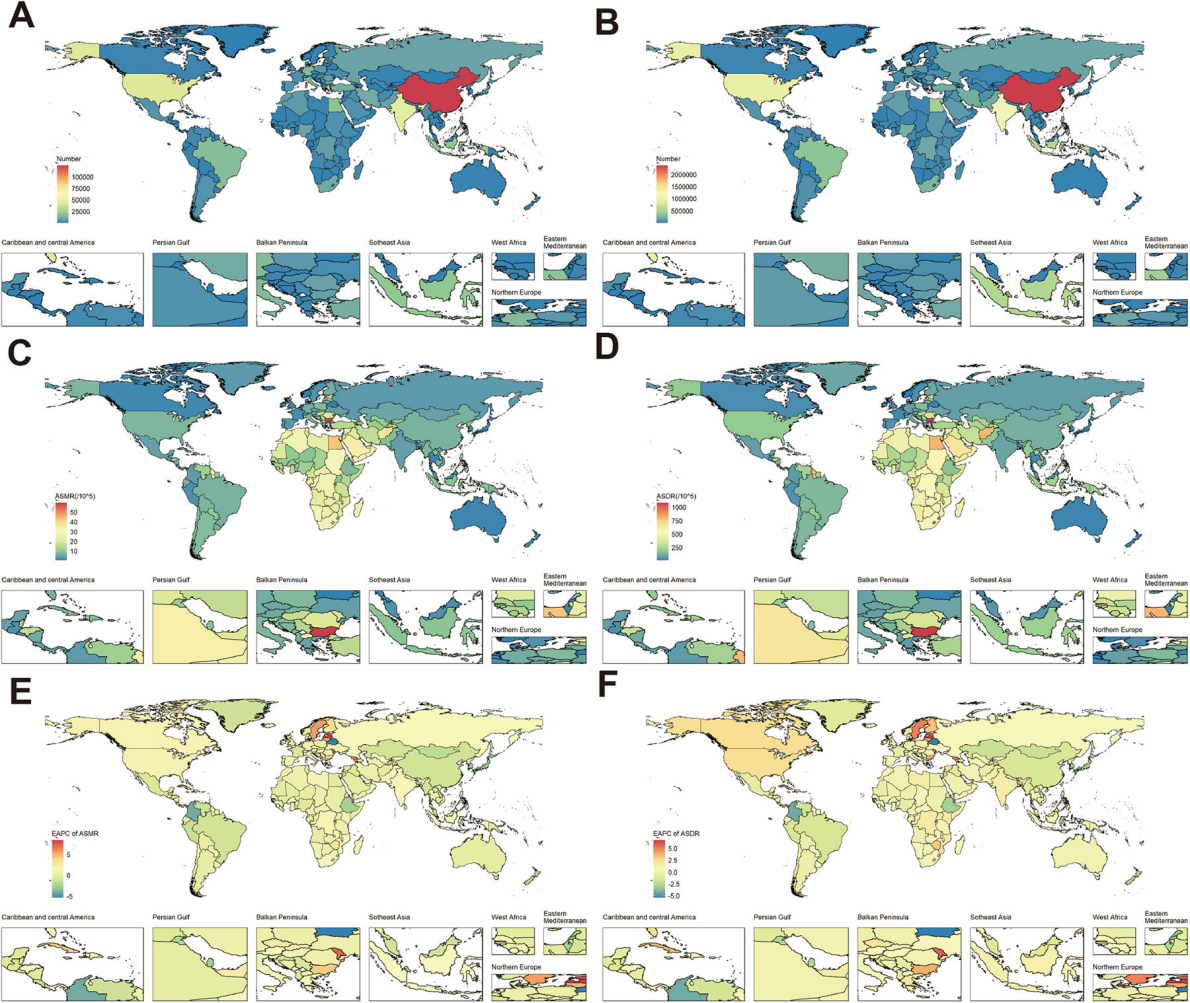
Burden of high BMI-related HHD in 204 countries in 2021: **(A)** deaths, **(B)** DALYs, **(C)** ASMR, **(D)** ASDR. Trends in the burden of high BMI-related HHD from 1990 to 2021 across 204 countries: **(E)** EAPC of ASMR, **(F)** EAPC of ASDR. World map from “Global country administrative boundary data” by Resource and Environmental Science Data Platform.

Notably, China accounted for the highest absolute disease burden in 2021, with 124,764 (95% UI: 63,334–198,271) in deaths and 2,373,040 (95% UI: 1,485,461–3,462,005) in DALYs, respectively (Supplementary Tables S4 and S5; Figures 8A,B). Geographic disparities in ASR were striking. The country with the highest ASMR was Bulgaria, followed by Lesotho and Egypt. In contrast, Belarus had the lowest ASMR (Figure 8C). The results for ASDR differ slightly from those of ASMR, with Bulgaria, Lesotho, and Eswatini exhibiting the highest ASDR, while Norway, Japan, and Belgium showed the lowest ASDR (Figure 8D).

Trend analysis also showed divergent trajectories. Following the integrated cluster analysis of the EAPC, it was observed that 4 out of 204 countries experienced a significant increase, 18 countries showed a slight increase, 175 countries remained stable or exhibited a slight decrease, and 7 countries encountered a significant decrease (Supplementary Figure S3). The EAPC of ASMR saw the most substantial increases in Latvia, Estonia, and the Republic of Moldova. Conversely, Belarus achieved the largest ASMR reductions, followed by Colombia. The countries that exhibited the most significant increases and decreases in ASDR were largely consistent with those observed in ASMR (Supplementary Tables S4 and S5; Figures 8E,F).

### 3.6 Correlation and frontier analysis with SDI

**Figure 9:**
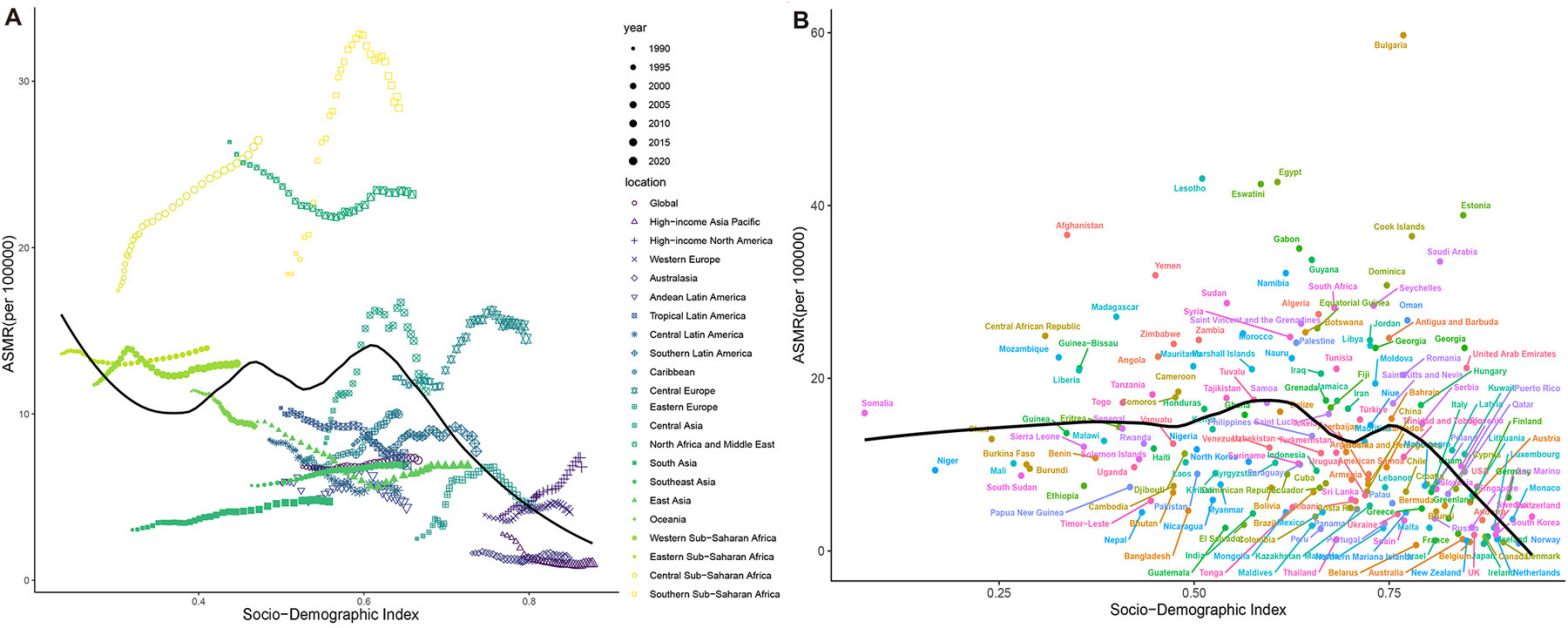
Correlation between ASMR of high BMI-related HHD and SDI from 1990 to 2021, stratified by region or country: **(A)** 21 GBD regions, **(B)** 204 countries.

The SDI of high BMI-related HHD exhibited a clear nonlinear relationship with ASDR and ASMR (Figure 9; Supplementary Figure S4). When SDI is less than 0.6, ASMR and ASDR fluctuate in a “W” pattern; however, when SDI exceeds 0.6, ASR decreases (Figure 9A). The ASMR and ASDR values for Southern Sub-Saharan Africa, Central Sub-Saharan Africa, and North Africa and Middle East were significantly higher than expected based on their SDI levels, whereas the High-Income Asia Pacific, Australasia, South Asia, and other regions were below expectations (Figure 9A; Supplementary Figure S4A). The findings from the country-level analysis align with the regional trends, as Bulgaria, Lesotho, and Egypt were notably higher than expected, while New Zealand and Australia were significantly lower than anticipated (Figure 9B; Supplementary Figure S4B).

**Figure 10:**
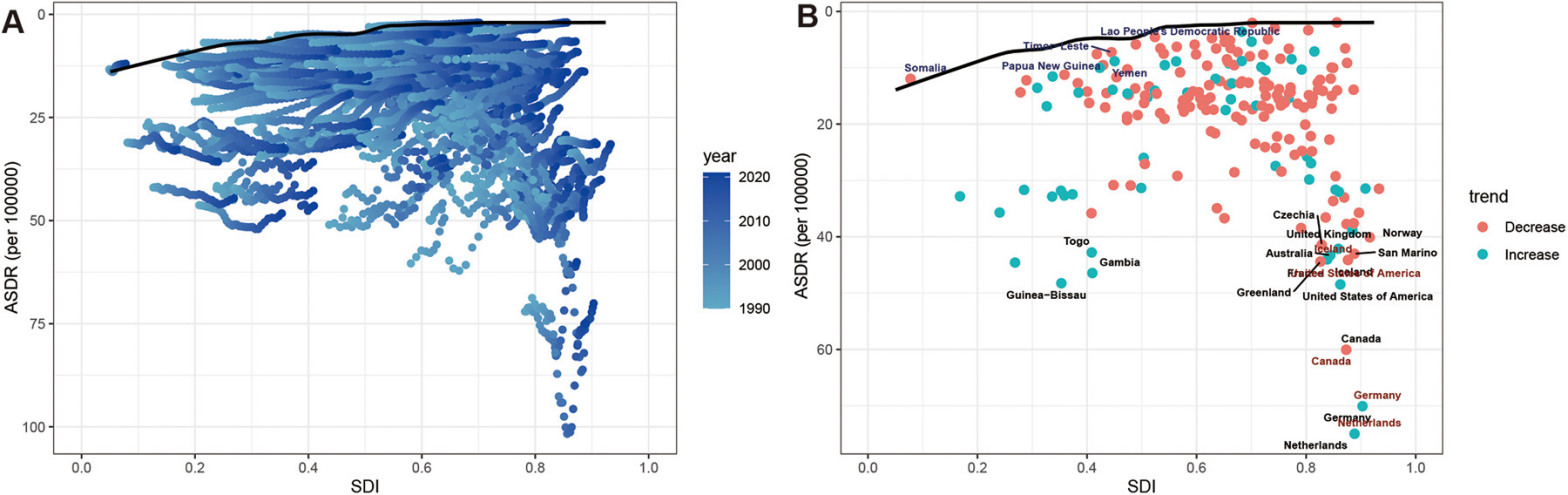
Frontier analysis of ASDR in high BMI-related HHD and SDI. **(A)** 1990–2021 **(B)** 2021. Dots represent countries, borders are drawn in black, and gaps between dots and borders are identified as effective differences.

Figure 10 illustrates the unrealized health gains for countries across varying SDIs. The top ten countries or regions exhibiting the largest effective differences from the frontier (effective difference: 41.27–72.99) include the Netherlands, Germany, Canada, the United States of America, Guinea-Bissau, Greenland, Iceland, France, Gambia, and Australia. Conversely, countries with lower SDIs, such as Somalia, Papua New Guinea, and Timor-Leste, demonstrate smaller effective differences. Frontier analysis indicates that countries with higher SDIs possess a greater potential for improvement in mitigating disease burden compared to those with lower SDIs.

### 3.7 Sex disparity of high BMI-related HHD burden

**Figure 11:**
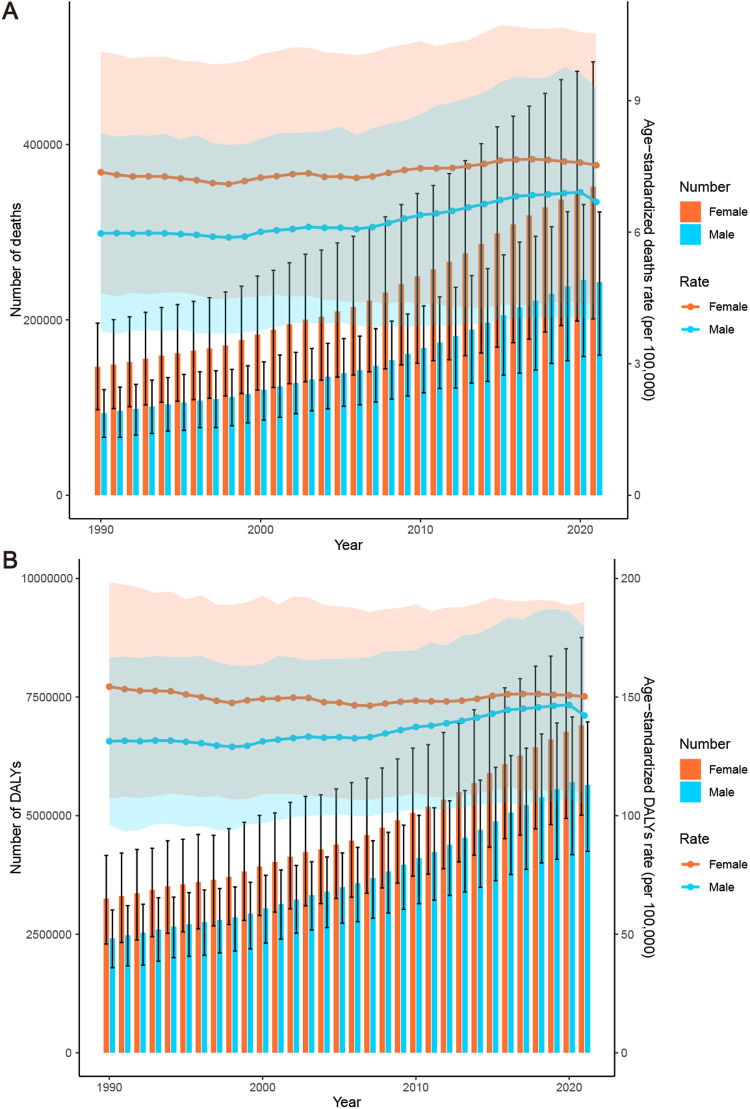
Burden of high BMI-related HHD from 1990 to 2021, stratified by sex: **(A)** deaths and ASMR, **(B)** DALYs and ASDR.

There are significant gender differences in the burden of high BMI-related HHD, with women consistently experiencing a higher burden than men (Figure 11). In 2021, the ASMR for females was 7.53 (95% UI: 4.36–10.53) per 100,000, which exceeded the male ASMR of 6.69 (95% UI: 3.98–9.3) per 100,000 (Table 1). The deaths for females were also 45% higher than those of males (Table 1). DALYs and ASDR also demonstrated similar gender differences (Figure 11B). This significant gender difference reminds us of the need to pay special attention to the disproportionate risks faced by women and their potential biological or social determinants.

Over the past 30 years, both the ASMR and ASDR have increased among men, with an EAPC of 0.55 (95% CI: 0.47–0.63) and 0.40 (95% CI: 0.34–0.47), respectively. In contrast, the ASMR in women has experienced a slight increase, with an EAPC of 0.20 (95% CI: 0.15–0.25), while the ASDR has shown a slight downward trend, with an EAPC of −0.04 (95% CI: −0.09–0.01) (Tables 1, 2). Concurrently, the total deaths and DALYs for both men and women have generally exhibited an upward trend (Figure 11).

### 3.8 High BMI-related HHD burden in age groups

**Figure 12:**
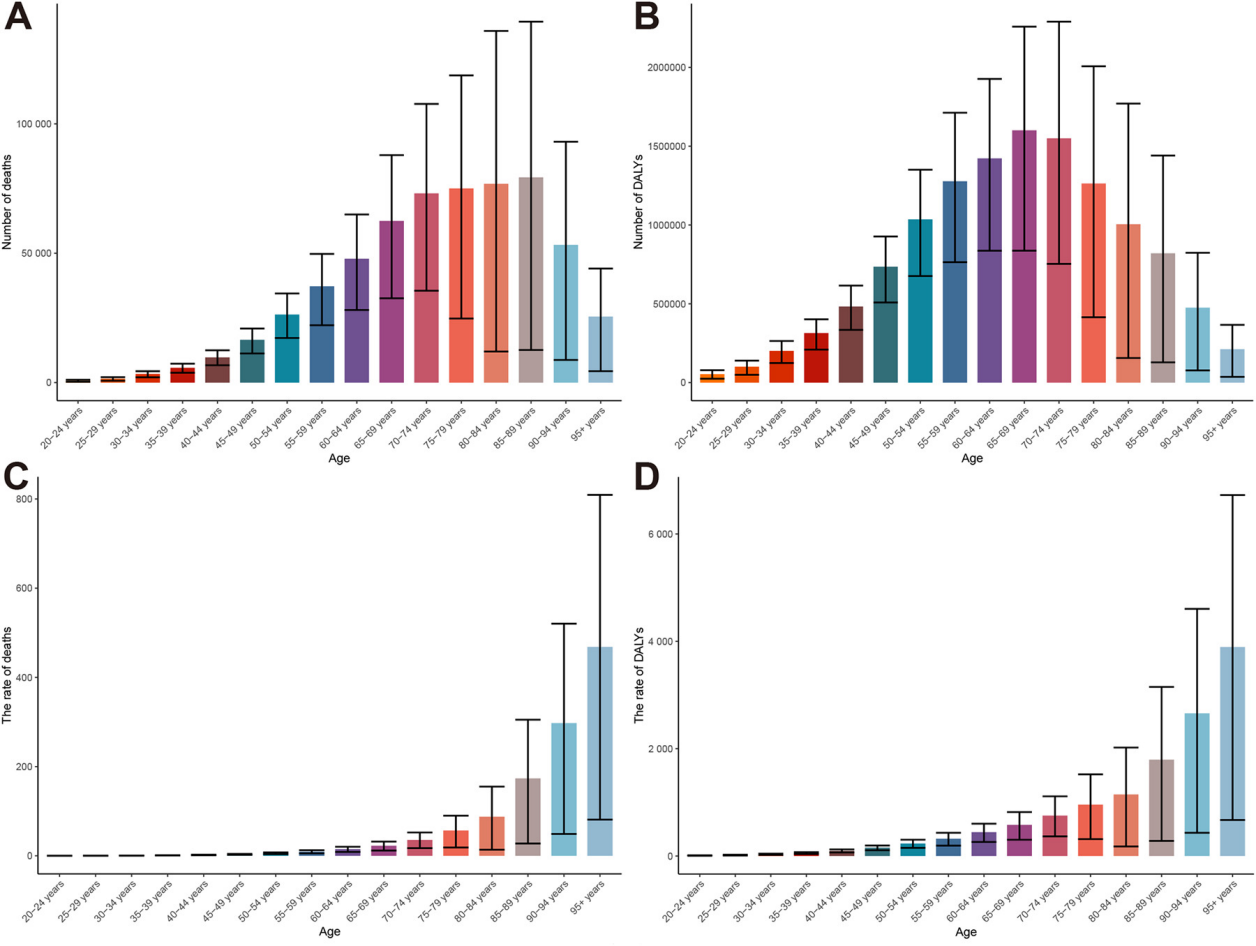
Global burden of high BMI-related HHD in 2021, stratified by age group: **(A)** deaths, **(B)** DALYs, **(C)** mortality rate, **(D)** DALY rate.

The disease burden concentrated heavily in older adults. In 2021, the distribution of deaths and DALYs across age groups exhibited an unimodal pattern. Deaths were predominantly concentrated among individuals aged 65–89 years, with the highest number recorded in the 85–89 age group at 79,346.87 (95% UI: 12,619.27–139,515.05) (Figure 12A). DALYs were primarily concentrated in the 55–79 age range, with the 65–69 age group exhibiting the highest level at 1,600,607.01 (95% UI: 836,837.70–2,257,960.31) (Figure 12B). Both mortality and DALY rates displayed a right-skewed distribution. The peak values were observed in individuals over 95 years old, with mortality rates reaching 468.40 (95% UI: 81.28–809.19) per 100,000 population and DALYs at 3.89 thousand (95% UI: 0.67–6.72 thousand) per 100,000 population, respectively (Figures 12C,D). From 1990 to 2021, mortality and DALY rates in the age group under 85 years remained generally stable or showed slight increases, while there was a significant rise in rates for those aged 85 and older, particularly in the group over 95 years old (Supplementary Figures S5C,D). Concurrently, the deaths and DALYs across each age group predominantly exhibited an upward trend (Supplementary Figures S5A,B).

APC analysis has once again confirmed that the risk of death and DALYs significantly increases with age, particularly among the elderly. Regarding period effects, disease risks have risen globally in recent years, especially in high-income countries, where the relative risk of death has increased to 1.29. Moreover, the global risk of death and DALYs has gradually escalated over time, particularly among cohorts born after 1995, indicating a growing burden on younger generations. Notably, this trend is more pronounced in high SDI regions (Supplementary Figures S6 and S7). This finding warns us that the long-term negative impact of high BMI on health is spreading to younger populations, potentially leading to greater challenges in disease burden in the coming decades.

### 3.9 Predictions of the disease burden of high BMI-related HHD

**Figure 13:**
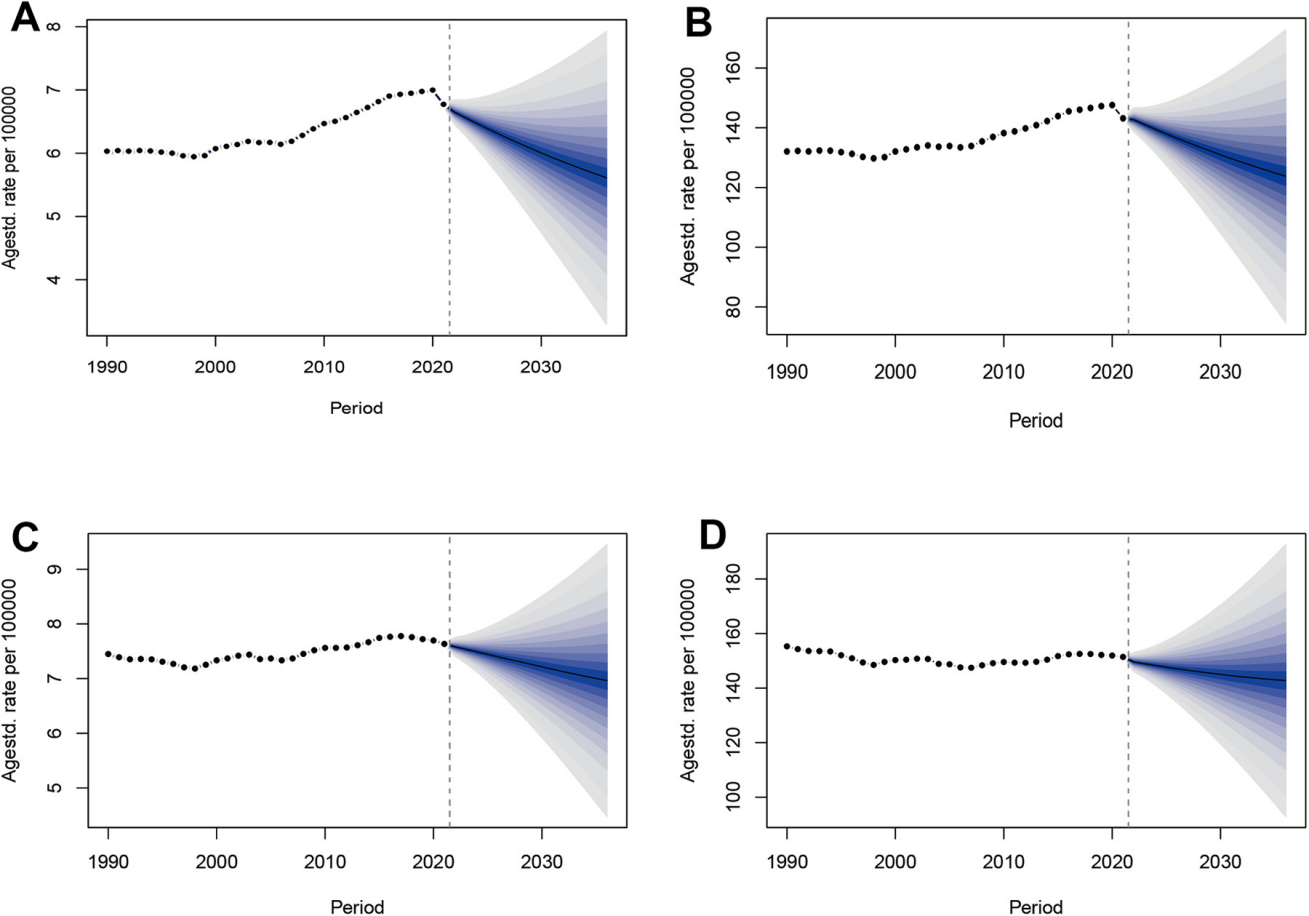
Trends in ASMR and ASDR for high BMI-related HHD from 1990 to 2036, observed and predicted using the BAPC model by sex: **(A)** male ASMR, **(B)** male ASDR, **(C)** female ASMR, **(D)** female ASDR.

The results of the two predictive models indicate a common trend: from 2021 to 2036, the disease burden is expected to show a slight downward trend, yet it will remain at a high level overall (Figure 13; Supplementary Figure S8). The BAPC model indicates that from 2021 to 2035, the male ASMR is expected to decrease from 6.78 per 100,000 to 5.61, while the ASDR will decline from 143.17 per 100,000 to 123.77. For females, the ASMR is projected to drop from 7.64 per 100,000 to 6.96, and the ASDR will decrease from 151.42 per 100,000 to 142.75, with a more pronounced reduction observed in males. This trend is confirmed by the separate ARIMA model. Both models exhibit a high degree of concordance in the core trend of slight decline, confirming the robustness of the predictions (Supplementary Figure S8). Additionally, the age and gender stratified analysis based on BAPC indicates that the mortality and DALY rates for men across all age groups have shown varying degrees of decline, particularly in those over 60 years old (Supplementary Figure S9). In contrast, women's rates have increased in the 20–44 age group and remained stable in the 45–64 age group, with a gradual decline observed after the age of 65 years (Supplementary Figure S10). Critically, despite projected improvements, elderly populations will continue carrying the highest disease burden among all age groups.

## 4 Discussion

This study comprehensively assesses the global impact of overweight and obesity on HHD, utilizing high BMI as the primary indicator. Our findings reveal that the burden of HHD attributable to high BMI continues to escalate worldwide. Over the past 30 years, the absolute deaths and DALYs have more than doubled, with standardized rates also showing an upward trend. In 2021, the PAF for deaths and DALYs reached alarming levels of 44% and 49%, respectively. This is a key strength of this study, indicating that nearly half of the disease burden is related to high BMI.

From a global perspective, the contribution of population growth is particularly pronounced, especially in low SDI regions. In contrast, regions with higher SDI exhibit a relatively balanced impact from both aging and population growth. Worryingly, high-income regions such as North America and Western Europe have experienced the most substantial increases in the disease burden, underscoring the growing health inequalities and the complexity of global public health responses.

The disease burden of high BMI-related HHD exhibits significant regional variations, with middle SDI and low SDI regions accounting for the majority of this burden. Specifically, East Asia, North Africa and Middle East, and South Asia are the most severely affected areas. Social and economic development has led these regions to confront several common challenges. Firstly, the widespread adoption of Westernized lifestyles has led to the rapid expansion of energy-dense and nutritionally imbalanced foods across the upstream and downstream sectors of their supply chains (26). Furthermore, urbanization has normalized a sedentary “office work” model, while the proliferation of electronic devices has contributed to a decline in physical activity (27). Additionally, urbanization has diminished public spaces available for exercise. Relevant studies indicate that South Asian adults may spend up to seven hours a day in sedentary activities, with insufficient physical activity even during leisure time (28, 29). Furthermore, healthcare challenges are evident. A study indicated that 92.6% of hypertensive patients in Sudan are obese (30), while another study confirmed that approximately 50% of patients in the Middle East and North Africa are aware of their hypertension; however, only one-fifth of these patients actively attempt to manage their blood pressure (31). This highlights the inadequate emphasis on health education regarding related diseases in these regions. Concurrently, it underscores the scarcity of medical resources and services, which includes insufficient testing equipment, a lack of effective medications, a shortage of medical personnel, and the absence of relevant medical policies (32).

In addition to common challenges, region-specific issues are also an important characteristic of the burden of obesity-related HHD, especially in economically underdeveloped areas. A notable issue in the African region is the coexistence of obesity and malnutrition, which is rooted in social stratification and complicates the implementation of food system policies (33). Furthermore, South Asian labor immigrants in the Middle East are a focal point of concern, exhibiting the highest rates of obesity and hypertension, which reach 80% and 38%, respectively (34). Notably, the cultural norm of “beautifying obesity” in South Africa has contributed to a concealed rise in chronic diseases such as hypertension, which are often overlooked over time (35). Conversely, weight stigma prevalent elsewhere can reduce medication adherence and indirectly increase hypertension risk through psychological and dietary pathways (36, 37). As a representative country in East Asia, China currently bears the largest global disease burden and faces unique challenges, including a substantial population base, an aging demographic, ethnic diversity, and health disparities between urban and rural areas (38, 39). Therefore, developing relevant policies requires creating differentiated interventions that account for the social, cultural, and environmental characteristics of different regions.

While high SDI regions exhibit a lower absolute disease burden, their increasing rates warrant attention. Frontier analyses indicate that high SDI regions possess greater health potential. Taking high-income North America as an example, the region's more sophisticated health care services contribute to a low absolute disease burden. However, factors such as an aging population, unhealthy diets, lack of exercise, and prolonged high-stress lifestyles contribute to a rising burden of obesity-related HHD. A cross-sectional study indicated that health inequalities in the United States are exacerbated by low-income groups and racial factors (40). In high-income regions, the availability of medications and surgical options far exceeds that in low-income areas. New weight loss medications, such as GLP-1, and metabolic surgery have been shown to effectively reduce weight and lower blood pressure, potentially decreasing reliance on antihypertensive medications. Nevertheless, challenges remain in their widespread adoption due to concerns regarding safety and cost (41). Additionally, inadequate health insurance policies related to obesity pose another issue; only 11% of market health plans in the United States cover weight loss medications, and “insufficient drug reimbursement” remains a significant concern (42).

A consistent finding is the disproportionately higher burden borne by women compared to men. In low- and middle-income countries, the prevalence of obesity in women is more than twice that of their male counterparts. Obese women are twice as likely to experience hypertension compared to non-obese women (43). Reduced estrogen levels are currently considered a significant factor contributing to the increased prevalence of hypertension in postmenopausal obese women. However, recent studies have also shown that obesity diminishes the cardiovascular protective effects of estrogen in premenopausal women (44). Furthermore, factors such as genetic and chromosomal differences, immune system responses, oxidative stress, and the sympathetic nervous system, particularly in postmenopausal women, exhibit notable gender biases (45). A particularly vulnerable group that warrants attention is those experiencing obesity-related gestational hypertension. Research indicates that being overweight or obese significantly heightens the risk of gestational hypertension, which in turn increases the likelihood of sustained postpartum hypertension by 2.35 times, thereby affecting the incidence of obesity and HHD in subsequent generations (46–48). Predictive analyses suggest that although the overall disease burden for both men and women is expected to decline in the future, the rates of mortality and DALYs among young and middle-aged women are projected to rise. This highlights the urgency of the need to focus on these groups.

The current disease burden is predominantly concentrated among the elderly population. Aging exacerbates the obesity-related cardiovascular disease burden through mechanisms such as oxidative stress, inflammation, and metabolic dysregulation (49). Furthermore, elderly patients often experience degenerative changes in cardiac structure and function and are significantly impacted by chronic comorbidities, which complicate their clinical manifestations and prognosis (49). However, the PAF analysis results indicate that young individuals have already assumed a significant role in the risk of disease burden. Concurrently, the APC model cohort effect reveals that new generations in high SDI regions are facing more severe disease risks, followed by those in low-middle and low-SDI regions. Consequently, new policies should consider measures throughout the patient's life cycle, with appropriate attention directed toward young people and even children.

In middle and low SDI regions, it is essential to focus on enhancing food system strategies, such as implementing food labeling, imposing taxes on unhealthy foods, providing subsidies for healthy foods, and promoting healthy eating initiatives. These measures have been positively validated in countries such as Chile and Mexico (50). Furthermore, investing in green infrastructure, like parks and walking trails, is crucial for promoting physical activity. For instance, Colombia's Ciclovía program promotes physical activity by closing streets to pedestrians and cyclists (51). Community health education campaigns should leverage local volunteers, schools, and media to promote awareness of the risks associated with obesity and hypertension, as well as the importance of healthy lifestyles. Strengthening primary healthcare systems is equally important, ensuring timely access to affordable treatments for hypertension and obesity. Additionally, school-based programs can play a pivotal role in early detection and prevention, incorporating regular health screenings and health education into primary and secondary education systems (e.g., China's Chinese Student Nutrition Day and Happy 10 Minutes) (52). Policies in high SDI regions should prioritize precision health interventions, such as leveraging wearable devices and digital health platforms to deliver personalized dietary and exercise recommendations (53, 54). Promoting advanced therapeutic options, including GLP-1 receptor agonists and bariatric surgery, is essential, and ensuring these are covered by health insurance policies will improve accessibility for high-risk populations. Additionally, policy-driven health taxes on sugary drinks and high-fat foods could incentivize healthier consumer behaviors. Following the implementation of sugar taxes in the Philippines, sales of sugary drinks decreased by 8.7% (55). Addressing health inequalities—such as those associated with income, race, and urban-rural divides—is another critical priority. For example, the Canadian Community Paramedicine(CP@clinic) integrates social support systems with healthcare networks, improving access to prevention and treatment services for vulnerable populations (56). Globally, all regions should focus on changing cultural norms around obesity. Comprehensive health education campaigns and media-driven behavior change initiatives can address these cultural barriers.

This study, however, has certain limitations. First, issues related to data availability and quality may arise from variations in statistical systems across different countries. Future research should aim to enhance data collection efforts in low SDI regions, such as Africa and South Asia. Additionally, the measurement of obesity in this study primarily relied on BMI, which does not differentiate between lean body mass and fat mass (12). Future studies could incorporate biological indicators, such as waist circumference and the Triple Mass Index, to conduct a more comprehensive analysis of an individual's fat distribution and visceral fat. Thirdly, although there is an acknowledged pathophysiological link between obesity, diabetes, and hypertension, this study was unable to obtain and include prevalence data for diabetes to explore its specific association with resistant hypertension. Therefore, future research, when data becomes available, should focus on analyzing diabetes and its association with resistant hypertension to gain a more comprehensive understanding of this complex relationship. Fourth, although we employed the ARIMA model to verify the stability and sensitivity of the BAPC predictions, which enhances confidence in the predicted trends, it is important to note that all models have their inherent limitations. For instance, the predictive assumptions of the BAPC model are based on the premise that future economic levels, healthcare intervention strategies, and other factors will not undergo significant changes. This may result in discrepancies between the actual burden and the current predictions. Therefore, future research could utilize larger datasets and explore hybrid predictive models to further refine the results. Finally, while the GBD database encompasses a substantial amount of global data, it still lacks key variables, including genetic susceptibility, social environmental factors (such as violence and pollution), and mental health, which limits the understanding of the relationship between obesity and HHD. Future research should further elucidate the complex disease burden associated with obesity-related HHD through the integration of multi-dimensional data.

## 5 Conclusions

The burden of obesity-related HHD represents not only a public health issue but also a multifaceted challenge that encompasses socioeconomic, cultural, lifestyle, and psychological dimensions. Over the past 30 years, there has been a significant increase in deaths and DALYs associated with high BMI-related HHD. Notably, by 2021, the PAF for deaths and DALYs reached 44% and 49%, respectively, which constitutes a key strength of the study. Significant regional disparities and health inequalities persist globally, with low and middle SDI regions facing particularly severe challenges. Furthermore, women and the elderly bear a disproportionate burden of this disease. Additionally, the research findings challenge traditional assumptions by demonstrating that the vulnerability of both young people and high-income individuals is on the rise. Consequently, countries should tailor interventions to their unique socioeconomic contexts, demographic structures, and cultural backgrounds. Simultaneously, ongoing efforts in public health promotion, nutritional intervention, and medical policy development will be critical in addressing this global challenge. Future public health strategies should emphasize comprehensive interventions and the establishment of a collaborative health promotion system that integrates social, economic, and cultural perspectives.

## Data Availability

The original contributions presented in the study are included in the article/Supplementary Material, further inquiries can be directed to the corresponding author.
