# Supplementary material for: Global burden of hypertensive heart disease attributable to high body mass index from 1990 to 2021: a multidimensional analysis and public health response
Source: Front Cardiovasc Med. 2025 Aug 12;12:1570390. doi: 10.3389/fcvm.2025.1570390 (PMC12379062; doi:10.3389/fcvm.2025.1570390)
Supplement: Supplementary file 1 [file Table1.docx]

Supplementary Table S1 The PAF of deaths and DALYs for hypertensive heart disease attributable to high BMI various regions in 1990 and 2021.

| measure | location | year | PAF (%) | measure | location | year | PAF (%) |
| --- | --- | --- | --- | --- | --- | --- | --- |
| Deaths | Global | 1990 | 32.68 (21.23 to 44.74) | DALYs | Global | 1990 | 35.65 (27.71 to 44.17) |
| Deaths | Global | 2021 | 44.22 (27.90 to 59.64) | DALYs | Global | 2021 | 48.88 (38.38 to 59.61) |
| Deaths | High SDI | 1990 | 45.38 (27.48 to 61.98) | DALYs | High SDI | 1990 | 51.09 (38.73 to 63.31) |
| Deaths | High SDI | 2021 | 56.89 (33.97 to 74.25) | DALYs | High SDI | 2021 | 65.11 (51.83 to 76.34) |
| Deaths | High-middle SDI | 1990 | 37.13 (22.46 to 52.50) | DALYs | High-middle SDI | 1990 | 40.43 (29.87 to 51.45) |
| Deaths | High-middle SDI | 2021 | 47.96 (24.42 to 67.26) | DALYs | High-middle SDI | 2021 | 52.19 (36.40 to 66.80) |
| Deaths | Middle SDI | 1990 | 27.35 (17.27 to 38.75) | DALYs | Middle SDI | 1990 | 30.59 (22.93 to 39.50) |
| Deaths | Middle SDI | 2021 | 40.99 (25.23 to 56.31) | DALYs | Middle SDI | 2021 | 45.94 (34.87 to 57.28) |
| Deaths | Low-middle SDI | 1990 | 29.06 (19.19 to 39.54) | DALYs | Low-middle SDI | 1990 | 32.09 (25.22 to 39.84) |
| Deaths | Low-middle SDI | 2021 | 38.75 (26.17 to 51.59) | DALYs | Low-middle SDI | 2021 | 43.98 (35.00 to 53.22) |
| Deaths | Low SDI | 1990 | 24.76 (17.32 to 33.85) | DALYs | Low SDI | 1990 | 28.50 (21.90 to 35.92) |
| Deaths | Low SDI | 2021 | 33.58 (23.19 to 46.50) | DALYs | Low SDI | 2021 | 38.93 (30.59 to 47.87) |
| Deaths | East Asia | 1990 | 22.75 (12.81 to 34.60) | DALYs | East Asia | 1990 | 25.56 (17.74 to 34.85) |
| Deaths | East Asia | 2021 | 36.78 (18.58 to 54.17) | DALYs | East Asia | 2021 | 41.11 (28.42 to 54.61) |
| Deaths | Oceania | 1990 | 43.67 (32.67 to 55.76) | DALYs | Oceania | 1990 | 50.00 (41.11 to 60.61) |
| Deaths | Oceania | 2021 | 49.10 (38.26 to 60.52) | DALYs | Oceania | 2021 | 56.30 (47.41 to 66.35) |
| Deaths | Southeast Asia | 1990 | 19.27 (13.73 to 26.41) | DALYs | Southeast Asia | 1990 | 23.07 (17.64 to 29.96) |
| Deaths | Southeast Asia | 2021 | 30.42 (21.81 to 40.53) | DALYs | Southeast Asia | 2021 | 36.22 (28.87 to 43.94) |
| Deaths | Central Asia | 1990 | 51.39 (36.27 to 65.79) | DALYs | Central Europe | 1990 | 56.79 (42.90 to 70.15) |
| Deaths | Central Asia | 2021 | 55.11 (34.94 to 71.81) | DALYs | Central Europe | 2021 | 61.41 (43.90 to 76.18) |
| Deaths | Central Europe | 1990 | 52.25 (33.08 to 70.05) | DALYs | Central Asia | 1990 | 56.50 (45.89 to 66.80) |
| Deaths | Central Europe | 2021 | 57.20 (31.51 to 77.01) | DALYs | Central Asia | 2021 | 59.86 (45.96 to 72.30) |
| Deaths | Southern Latin America | 1990 | 50.03 (31.09 to 68.14) | DALYs | Eastern Europe | 1990 | 60.13 (50.47 to 68.77) |
| Deaths | Southern Latin America | 2021 | 56.30 (27.95 to 77.43) | DALYs | Eastern Europe | 2021 | 64.92 (50.79 to 76.75) |
| Deaths | Eastern Europe | 1990 | 55.73 (41.94 to 68.58) | DALYs | High-income Asia Pacific | 1990 | 29.74 (19.60 to 41.80) |
| Deaths | Eastern Europe | 2021 | 60.57 (39.67 to 76.74) | DALYs | High-income Asia Pacific | 2021 | 38.69 (25.92 to 52.47) |
| Deaths | High-income Asia Pacific | 1990 | 26.30 (11.51 to 41.71) | DALYs | Australasia | 1990 | 52.56 (37.53 to 68.35) |
| Deaths | High-income Asia Pacific | 2021 | 33.50 (15.24 to 52.95) | DALYs | Australasia | 2021 | 62.74 (43.51 to 78.28) |
| Deaths | Western Europe | 1990 | 45.50 (22.73 to 66.04) | DALYs | Western Europe | 1990 | 49.46 (33.14 to 64.98) |
| Deaths | Western Europe | 2021 | 50.04 (18.11 to 74.51) | DALYs | Western Europe | 2021 | 53.52 (28.96 to 72.92) |
| Deaths | Australasia | 1990 | 47.20 (25.01 to 67.20) | DALYs | Southern Latin America | 1990 | 54.81 (41.08 to 68.49) |
| Deaths | Australasia | 2021 | 57.00 (27.17 to 78.47) | DALYs | Southern Latin America | 2021 | 60.94 (41.38 to 77.25) |
| Deaths | High-income North America | 1990 | 56.34 (39.10 to 71.88) | DALYs | High-income North America | 1990 | 63.09 (51.54 to 73.67) |
| Deaths | High-income North America | 2021 | 65.39 (45.79 to 80.48) | DALYs | High-income North America | 2021 | 72.81 (62.17 to 81.68) |
| Deaths | Caribbean | 1990 | 38.64 (26.94 to 51.44) | DALYs | Caribbean | 1990 | 43.98 (35.72 to 53.83) |
| Deaths | Caribbean | 2021 | 50.65 (35.24 to 65.30) | DALYs | Caribbean | 2021 | 56.44 (45.66 to 66.86) |
| Deaths | Andean Latin America | 1990 | 40.60 (26.31 to 55.74) | DALYs | Andean Latin America | 1990 | 47.00 (36.23 to 59.03) |
| Deaths | Andean Latin America | 2021 | 52.05 (29.91 to 71.27) | DALYs | Andean Latin America | 2021 | 57.95 (42.87 to 71.99) |
| Deaths | Central Latin America | 1990 | 45.32 (28.09 to 62.85) | DALYs | Central Latin America | 1990 | 50.49 (37.56 to 63.72) |
| Deaths | Central Latin America | 2021 | 56.09 (31.60 to 75.13) | DALYs | Central Latin America | 2021 | 61.05 (44.34 to 75.68) |
| Deaths | Tropical Latin America | 1990 | 44.83 (31.23 to 59.04) | DALYs | North Africa and Middle East | 1990 | 50.91 (38.76 to 62.39) |
| Deaths | Tropical Latin America | 2021 | 55.70 (34.95 to 73.65) | DALYs | North Africa and Middle East | 2021 | 63.62 (47.86 to 76.56) |
| Deaths | North Africa and Middle East | 1990 | 46.35 (29.21 to 62.25) | DALYs | Tropical Latin America | 1990 | 50.87 (40.79 to 61.51) |
| Deaths | North Africa and Middle East | 2021 | 58.74 (35.27 to 76.78) | DALYs | Tropical Latin America | 2021 | 61.09 (46.93 to 73.85) |
| Deaths | South Asia | 1990 | 16.97 (11.26 to 24.62) | DALYs | Central Sub-Saharan Africa | 1990 | 29.40 (22.65 to 37.48) |
| Deaths | South Asia | 2021 | 28.68 (19.14 to 40.46) | DALYs | Central Sub-Saharan Africa | 2021 | 45.10 (34.78 to 56.53) |
| Deaths | Southern Sub-Saharan Africa | 1990 | 48.29 (31.88 to 64.04) | DALYs | South Asia | 1990 | 19.70 (14.71 to 26.30) |
| Deaths | Southern Sub-Saharan Africa | 2021 | 59.90 (38.63 to 76.78) | DALYs | South Asia | 2021 | 33.64 (25.96 to 42.54) |
| Deaths | Eastern Sub-Saharan Africa | 1990 | 23.30 (15.64 to 32.48) | DALYs | Eastern Sub-Saharan Africa | 1990 | 27.03 (20.36 to 34.53) |
| Deaths | Eastern Sub-Saharan Africa | 2021 | 32.80 (21.62 to 46.09) | DALYs | Eastern Sub-Saharan Africa | 2021 | 37.89 (29.34 to 47.25) |
| Deaths | Central Sub-Saharan Africa | 1990 | 25.79 (17.75 to 35.56) | DALYs | Southern Sub-Saharan Africa | 1990 | 54.36 (43.93 to 64.91) |
| Deaths | Central Sub-Saharan Africa | 2021 | 40.03 (26.28 to 54.86) | DALYs | Southern Sub-Saharan Africa | 2021 | 65.02 (51.46 to 76.58) |
| Deaths | Western Sub-Saharan Africa | 1990 | 34.04 (23.94 to 45.53) | DALYs | Western Sub-Saharan Africa | 1990 | 38.16 (30.19 to 46.73) |
| Deaths | Western Sub-Saharan Africa | 2021 | 45.28 (31.59 to 59.36) | DALYs | Western Sub-Saharan Africa | 2021 | 50.09 (40.10 to 60.11) |
| Deaths | North Africa and Middle East | 1990 | 46.35 (29.21 to 62.25) | DALYs | North Africa and Middle East | 1990 | 50.91 (38.76 to 62.39) |
| Deaths | North Africa and Middle East | 2021 | 58.74 (35.27 to 76.78) | DALYs | North Africa and Middle East | 2021 | 63.62 (47.86 to 76.56) |
| Deaths | South Asia | 1990 | 16.97 (11.26 to 24.62) | DALYs | South Asia | 1990 | 19.70 (14.71 to 26.30) |
| Deaths | South Asia | 2021 | 28.68 (19.14 to 40.46) | DALYs | South Asia | 2021 | 33.64 (25.96 to 42.54) |
