# Supplementary material for: Global burden of hypertensive heart disease attributable to high body mass index from 1990 to 2021: a multidimensional analysis and public health response
Source: Front Cardiovasc Med. 2025 Aug 12;12:1570390. doi: 10.3389/fcvm.2025.1570390 (PMC12379062; doi:10.3389/fcvm.2025.1570390)
Supplement: Supplementary file 2 [file Table2.docx]

Supplementary Table S2 Global PAF of deaths and DALYs for hypertensive heart disease attributable to high BMI by age in 1990 and 2021.

| measure | location | age | year | PAF (%) | measure | location | age | year | PAF (%) |
| --- | --- | --- | --- | --- | --- | --- | --- | --- | --- |
| Deaths | Global | 20-24 years | 1990 | 39.26 (16.08 to 60.45) | DALYs | Global | 20-24 years | 1990 | 39.42 (16.16 to 60.58) |
| Deaths | Global | 20-24 years | 2021 | 56.85 (25.26 to 78.64) | DALYs | Global | 20-24 years | 2021 | 57.13 (25.44 to 78.83) |
| Deaths | Global | 25-29 years | 1990 | 46.33 (20.35 to 67.72) | DALYs | Global | 25-29 years | 1990 | 46.55 (20.46 to 67.86) |
| Deaths | Global | 25-29 years | 2021 | 65.35 (31.99 to 86.24) | DALYs | Global | 25-29 years | 2021 | 65.58 (32.17 to 86.33) |
| Deaths | Global | 30-34 years | 1990 | 50.70 (28.62 to 69.35) | DALYs | Global | 30-34 years | 1990 | 50.88 (28.77 to 69.51) |
| Deaths | Global | 30-34 years | 2021 | 69.55 (44.62 to 86.27) | DALYs | Global | 30-34 years | 2021 | 69.67 (44.73 to 86.35) |
| Deaths | Global | 35-39 years | 1990 | 50.71 (31.71 to 67.97) | DALYs | Global | 35-39 years | 1990 | 50.81 (31.79 to 68.09) |
| Deaths | Global | 35-39 years | 2021 | 70.26 (48.65 to 86.55) | DALYs | Global | 35-39 years | 2021 | 70.31 (48.66 to 86.60) |
| Deaths | Global | 40-44 years | 1990 | 49.23 (31.35 to 65.02) | DALYs | Global | 40-44 years | 1990 | 49.30 (31.41 to 65.09) |
| Deaths | Global | 40-44 years | 2021 | 68.04 (48.40 to 83.62) | DALYs | Global | 40-44 years | 2021 | 68.08 (48.46 to 83.64) |
| Deaths | Global | 45-49 years | 1990 | 48.39 (32.34 to 63.27) | DALYs | Global | 45-49 years | 1990 | 48.45 (32.41 to 63.32) |
| Deaths | Global | 45-49 years | 2021 | 65.91 (47.33 to 81.32) | DALYs | Global | 45-49 years | 2021 | 65.94 (47.35 to 81.33) |
| Deaths | Global | 50-54 years | 1990 | 45.87 (28.02 to 61.67) | DALYs | Global | 50-54 years | 1990 | 45.94 (28.07 to 61.74) |
| Deaths | Global | 50-54 years | 2021 | 63.40 (42.09 to 79.64) | DALYs | Global | 50-54 years | 2021 | 63.44 (42.13 to 79.68) |
| Deaths | Global | 55-59 years | 1990 | 42.41 (24.39 to 59.38) | DALYs | Global | 55-59 years | 1990 | 42.44 (24.42 to 59.42) |
| Deaths | Global | 55-59 years | 2021 | 59.57 (36.76 to 77.06) | DALYs | Global | 55-59 years | 2021 | 59.60 (36.82 to 77.11) |
| Deaths | Global | 60-64 years | 1990 | 39.14 (22.41 to 55.05) | DALYs | Global | 60-64 years | 1990 | 39.16 (22.42 to 55.07) |
| Deaths | Global | 60-64 years | 2021 | 55.29 (33.93 to 73.14) | DALYs | Global | 60-64 years | 2021 | 55.33 (33.99 to 73.19) |
| Deaths | Global | 65-69 years | 1990 | 36.21 (17.15 to 52.50) | DALYs | Global | 65-69 years | 1990 | 36.23 (17.18 to 52.53) |
| Deaths | Global | 65-69 years | 2021 | 51.11 (26.69 to 70.12) | DALYs | Global | 65-69 years | 2021 | 51.18 (26.71 to 70.15) |
| Deaths | Global | 70-74 years | 1990 | 31.84 (14.76 to 49.85) | DALYs | Global | 70-74 years | 1990 | 31.86 (14.77 to 49.84) |
| Deaths | Global | 70-74 years | 2021 | 45.74 (22.47 to 66.84) | DALYs | Global | 70-74 years | 2021 | 45.83 (22.53 to 67.00) |
| Deaths | Global | 75-79 years | 1990 | 30.62 (9.58 to 49.98) | DALYs | Global | 75-79 years | 1990 | 30.57 (9.56 to 49.96) |
| Deaths | Global | 75-79 years | 2021 | 41.81 (13.34 to 64.83) | DALYs | Global | 75-79 years | 2021 | 41.85 (13.36 to 64.87) |
| Deaths | Global | 80-84 years | 1990 | 26.60 (3.66 to 48.09) | DALYs | Global | 80-84 years | 1990 | 26.56 (3.65 to 48.04) |
| Deaths | Global | 80-84 years | 2021 | 35.58 (5.35 to 62.33) | DALYs | Global | 80-84 years | 2021 | 35.60 (5.35 to 62.37) |
| Deaths | Global | 85-89 years | 1990 | 27.91 (3.91 to 49.57) | DALYs | Global | 85-89 years | 1990 | 27.88 (3.90 to 49.53) |
| Deaths | Global | 85-89 years | 2021 | 37.48 (5.71 to 64.83) | DALYs | Global | 85-89 years | 2021 | 37.48 (5.71 to 64.87) |
| Deaths | Global | 90-94 years | 1990 | 29.61 (4.23 to 52.79) | DALYs | Global | 90-94 years | 1990 | 29.60 (4.23 to 52.77) |
| Deaths | Global | 90-94 years | 2021 | 39.73 (6.20 to 67.87) | DALYs | Global | 90-94 years | 2021 | 39.73 (6.20 to 67.89) |
| Deaths | Global | 95+ years | 1990 | 31.93 (4.63 to 56.02) | DALYs | Global | 95+ years | 1990 | 31.89 (4.62 to 55.98) |
| Deaths | Global | 95+ years | 2021 | 42.26 (6.67 to 70.80) | DALYs | Global | 95+ years | 2021 | 42.24 (6.67 to 70.79) |
