# Supplementary material for: Global burden of hypertensive heart disease attributable to high body mass index from 1990 to 2021: a multidimensional analysis and public health response
Source: Front Cardiovasc Med. 2025 Aug 12;12:1570390. doi: 10.3389/fcvm.2025.1570390 (PMC12379062; doi:10.3389/fcvm.2025.1570390)
Supplement: Supplementary file 3 [file Table3.docx]

Supplementary Table S3 Changes in deaths and DALYs according to population-level determinants and causes from 1990 to 2021.

| Location | DALYs changes due to population-level determinants* | | | Mortality changes due to population-level determinants* | | |
| --- | --- | --- | --- | --- | --- | --- |
|  | Ageing | Population | EC | Ageing | Population | EC |
| Global |  | | | | | |
| Both | 2230842.35 (32.4%) | 4604307.73 (66.87%) | 50585.17 (0.73%) | 135316.01 (38.14%) | 207243.34 (58.41%) | 12243.75 (3.45%) |
| Male | 990108.28 (30.64%) | 2004740.38 (62.04%) | 236641.6 (7.32%) | 55719.27 (37.28%) | 82321.24 (55.08%) | 11407.10 (7.63%) |
| Female | 1209463.31 (33.1%) | 2602553.90 (71.22%) | -157772.22 (-4.32%) | 77363.98 (37.67%) | 125120.13 (60.93%) | 2871.38 (1.4%) |
| High SDI |  | | | | | |
| Both | 401908.37 (39.03%) | 412269.77 (40.03%) | 215645.36 (20.94%) | 30857.85 (51.97%) | 21330.49 (35.92%) | 7187.88 (12.11%) |
| Male | 182022.47 (31.13%) | 217407.85 (37.18%) | 185375.06 (31.7%) | 11837.9 (44.83%) | 9144.65 (34.63%) | 5425.88 (20.55%) |
| Female | 217458.80 (48.87%) | 194459.19 (43.7%) | 33100.13 (7.44%) | 18671.77 (56.64%) | 11910.81 (36.13%) | 2385.21 (7.23%) |
| High-middle SDI |  | | | | | |
| Both | 646347.29 (59.16%) | 618273.81 (56.59%) | -172153.71 (-15.76%) | 43793.67 (57.05%) | 32355.60 (42.15%) | 609.05 (0.79%) |
| Male | 308187.45 (59.21%) | 292523.83 (56.2%) | -80198.12 (-15.41%) | 18626.18 (59.61%) | 13534.21 (43.31%) | -911.18 (-2.92%) |
| Female | 340806.45 (59.59%) | 324816.31 (56.79%) | -93668.53 (-16.38%) | 25312.99 (55.62%) | 18690.85 (41.07%) | 1505.31 (3.31%) |
| Middle SDI |  | | | | | |
| Both | 1227407.34 (53.92%) | 1714249.06 (75.3%) | -665182.67 (-29.22%) | 67472.50 (57.86%) | 74771.07 (64.11%) | -25621.96 (-21.97%) |
| Male | 543346.97 (47.88%) | 766973.23 (67.59%) | -175548.23 (-15.47%) | 28328.27 (52.31%) | 31444.94 (58.06%) | -5614.34 (-10.37%) |
| Female | 677851 (59.37%) | 951954.52 (83.38%) | -488103.76 (-42.75%) | 38737.69 (62.02%) | 43651.91 (69.88%) | -19926.85 (-31.9%) |
| Low-middle SDI |  | | | | | |
| Both | 287022.50 (16.78%) | 1247245.35 (72.92%) | 176083.31 (10.3%) | 15539.35 (21.48%) | 49932.59 (69.03%) | 6865.09 (9.49%) |
| Male | 84047.41 (11.57%) | 472618.01 (65.05%) | 169922.83 (23.39%) | 4296.69 (15.03%) | 17782.27 (62.22%) | 6502.40 (22.75%) |
| Female | 215514.25 (21.91%) | 784416.70 (79.74%) | -16168.058 (-1.64%) | 11957.66 (27.33%) | 32630.13 (74.57%) | -832.14 (-1.9%) |
| Low SDI |  | | | | | |
| Both | -41449.68 (-5.4%) | 771335.06 (100.46%) | 37926.90 (4.94%) | -1221.72 (-4.18%) | 28228.59 (96.65%) | 2198.92 (7.53%) |
| Male | -19526.77 (-7.5%) | 248431.94 (95.4%) | 31494.08 (12.09%) | -677.80 (-7.67%) | 8533.42 (96.59%) | 979.37 (11.09%) |
| Female | 13373.72 (-2.64%) | 525756.53 (103.62%) | -4969.78 (-0.98%) | -182.65 (-0.9%) | 19808.71 (97.24%) | 744.74 (3.66%) |

DALY–disability-adjusted life years, EC–Epidemiological change, SDI–sociodemographic index,*Percentages contribute to the total changes.
