# Supplementary material for: Global burden of hypertensive heart disease attributable to high body mass index from 1990 to 2021: a multidimensional analysis and public health response
Source: Front Cardiovasc Med. 2025 Aug 12;12:1570390. doi: 10.3389/fcvm.2025.1570390 (PMC12379062; doi:10.3389/fcvm.2025.1570390)
Supplement: Supplementary file 4 [file Table4.docx]

Supplementary Table S4 Deaths and ASMR of hypertensive heart disease attributable to high BMI in 204 countries and territories in 1990 and 2021, and the temporal trends from 1990 to 2021.

| **Deaths** | 1990 | | 2021 | | 1990–2021 |
| --- | --- | --- | --- | --- | --- |
| Location | Deaths cases  No. (95% UI) | ASMR per 100,000  no. (95% UI) | Deaths cases  no. (95% UI) | ASMR per 100,000  no. (95% UI) | EAPC in ASMR  no. (95% CI) |
| Afghanistan | 2338 (868 to 3962) | 36.97 (14.6 to 61.15) | 2995 (1428 to 4803) | 36.6 (17.84 to 58.05) | -0.12 (-0.24 to 0) |
| Albania | 125 (71 to 189) | 7.5 (3.95 to 11.64) | 237 (106 to 398) | 5.79 (2.45 to 9.91) | -0.42 (-0.66 to -0.18) |
| Algeria | 2237 (1375 to 3318) | 25.95 (12.71 to 42.5) | 7084 (3894 to 10626) | 27.42 (12.35 to 42.8) | 0.57 (0.42 to 0.73) |
| American Samoa | 2 (2 to 3) | 11.23 (7.39 to 15.15) | 4 (3 to 5) | 8.89 (6.26 to 11.59) | -0.98 (-1.25 to -0.71) |
| Andorra | 2 (1 to 4) | 5.14 (2.01 to 8.6) | 7 (2 to 12) | 3.59 (1.35 to 6.25) | -0.74 (-0.92 to -0.55) |
| Angola | 650 (340 to 967) | 19.45 (10.1 to 29.51) | 2099 (1288 to 3057) | 22.51 (13.29 to 35.02) | 0.19 (0.08 to 0.31) |
| Antigua and Barbuda | 9 (6 to 12) | 15.96 (10.32 to 21.71) | 23 (16 to 30) | 24.66 (15.05 to 33.16) | 1.76 (1.31 to 2.22) |
| Argentina | 2701 (1779 to 3529) | 8.97 (5.51 to 12.09) | 4863 (2452 to 6899) | 8.31 (4.25 to 11.72) | 0.16 (-0.02 to 0.34) |
| Armenia | 221 (136 to 309) | 9.73 (5.62 to 13.97) | 357 (187 to 515) | 8.28 (4.51 to 11.85) | -0.27 (-0.63 to 0.1) |
| Australia | 279 (154 to 405) | 1.55 (0.78 to 2.29) | 728 (285 to 1069) | 1.36 (0.62 to 1.95) | -0.1 (-0.51 to 0.31) |
| Austria | 567 (277 to 827) | 4.65 (2.32 to 6.75) | 1372 (431 to 2210) | 5.65 (2.05 to 8.88) | 1.59 (1.22 to 1.97) |
| Azerbaijan | 587 (380 to 839) | 13.71 (8.19 to 20.51) | 959 (611 to 1434) | 11.47 (6.75 to 17.63) | -0.21 (-0.44 to 0.02) |
| Bahamas | 42 (31 to 52) | 28.12 (20.15 to 36.02) | 141 (101 to 185) | 37.1 (24.71 to 50.31) | 1.12 (0.87 to 1.38) |
| Bahrain | 19 (14 to 25) | 18.32 (9.9 to 26.57) | 69 (45 to 98) | 15.32 (7.41 to 23.37) | -0.8 (-1.09 to -0.51) |
| Bangladesh | 1407 (807 to 2289) | 3.38 (1.84 to 5.75) | 5594 (2924 to 10799) | 4.68 (2.25 to 9.36) | 1.21 (0.97 to 1.45) |
| Barbados | 26 (16 to 36) | 8.77 (5.43 to 12.06) | 50 (29 to 71) | 9.71 (5.54 to 13.91) | 0.91 (0.75 to 1.07) |
| Belarus | 329 (234 to 438) | 2.55 (1.79 to 3.4) | 109 (73 to 145) | 0.69 (0.48 to 0.91) | -5.33 (-6.45 to -4.2) |
| Belgium | 174 (80 to 271) | 1.14 (0.5 to 1.79) | 339 (115 to 547) | 1.05 (0.44 to 1.64) | -0.36 (-0.95 to 0.23) |
| Belize | 9 (6 to 12) | 9.82 (6.85 to 12.66) | 45 (32 to 57) | 16.13 (10.59 to 21.24) | 2.11 (1.84 to 2.38) |
| Benin | 171 (98 to 243) | 8.95 (5.15 to 13.14) | 516 (294 to 733) | 10.78 (6.06 to 15.67) | 0.61 (0.5 to 0.73) |
| Bermuda | 3 (2 to 4) | 5.15 (3.2 to 6.98) | 8 (4 to 11) | 5.26 (3 to 7.3) | 0.47 (-0.08 to 1.02) |
| Bhutan | 15 (7 to 24) | 7.33 (3.3 to 12.18) | 38 (22 to 58) | 6.78 (3.89 to 10.56) | -0.28 (-0.34 to -0.22) |
| Bolivia (Plurinational State of) | 205 (84 to 321) | 7.5 (2.98 to 12.02) | 563 (278 to 865) | 7.32 (3.35 to 11.52) | -0.03 (-0.07 to 0.01) |
| Bosnia and Herzegovina | 234 (154 to 338) | 6.75 (4.27 to 10.19) | 493 (247 to 775) | 7.73 (3.94 to 12.11) | 0.69 (0.51 to 0.87) |
| Botswana | 106 (68 to 157) | 23.62 (14.36 to 38.48) | 289 (196 to 406) | 25.32 (14.94 to 36.56) | 0.61 (0.3 to 0.93) |
| Brazil | 8248 (6242 to 10305) | 10.41 (7.07 to 13.84) | 16586 (10637 to 21885) | 6.83 (4.26 to 9.14) | -1.2 (-1.35 to -1.05) |
| Brunei Darussalam | 5 (3 to 7) | 5.3 (2.9 to 8.32) | 12 (9 to 17) | 4.58 (2.53 to 6.96) | -0.12 (-0.34 to 0.11) |
| Bulgaria | 2157 (1606 to 2776) | 21.51 (13.33 to 29.43) | 8511 (4945 to 11904) | 59.71 (34.53 to 83.6) | 4.28 (3.49 to 5.07) |
| Burkina Faso | 288 (167 to 431) | 7.06 (3.98 to 11.01) | 869 (456 to 1391) | 10.01 (5.18 to 16.63) | 1.38 (1.28 to 1.48) |
| Burundi | 218 (18 to 379) | 10.72 (0.93 to 19.09) | 378 (179 to 567) | 9.53 (4.43 to 15.62) | -0.89 (-1.09 to -0.68) |
| Cabo Verde | 22 (13 to 32) | 9.45 (5.76 to 13.94) | 45 (28 to 64) | 10.48 (6.42 to 15.04) | 0.09 (-0.21 to 0.39) |
| Cambodia | 319 (149 to 502) | 7.47 (3.38 to 11.98) | 854 (444 to 1281) | 7.53 (3.89 to 11.72) | 0 (-0.15 to 0.15) |
| Cameroon | 670 (341 to 952) | 16.73 (8.05 to 24.42) | 2032 (950 to 3078) | 18.44 (8.55 to 28.66) | 0.27 (0.08 to 0.46) |
| Canada | 320 (190 to 439) | 1.01 (0.59 to 1.41) | 1262 (671 to 1736) | 1.66 (0.99 to 2.19) | 1.97 (1.56 to 2.37) |
| Central African Republic | 177 (58 to 299) | 18.27 (6.15 to 30.77) | 446 (169 to 750) | 24.91 (9.44 to 42.94) | 0.97 (0.91 to 1.03) |
| Chad | 270 (120 to 401) | 10.2 (4.38 to 15.69) | 690 (322 to 1066) | 12.97 (5.85 to 20.24) | 0.68 (0.44 to 0.91) |
| Chile | 624 (390 to 836) | 7.07 (4.08 to 9.82) | 1817 (824 to 2586) | 6.87 (3.16 to 9.76) | 0.28 (0 to 0.56) |
| China | 58894 (33794 to 82600) | 9.66 (4.72 to 14.65) | 124764 (63334 to 198271) | 6.9 (3.14 to 11.62) | -1.17 (-1.58 to -0.75) |
| Colombia | 1864 (1274 to 2480) | 12.36 (7.68 to 17.27) | 2276 (1172 to 3345) | 3.98 (2.09 to 5.81) | -4.08 (-4.3 to -3.85) |
| Comoros | 28 (13 to 45) | 17.34 (7.81 to 27.37) | 74 (40 to 118) | 17.8 (8.76 to 29.31) | -0.14 (-0.33 to 0.05) |
| Congo | 255 (116 to 397) | 27.03 (12.64 to 43.42) | 689 (384 to 1033) | 31.5 (16.46 to 49.46) | 0.24 (0.12 to 0.37) |
| Cook Islands | 7 (5 to 9) | 62.96 (44.14 to 83.76) | 9 (6 to 12) | 36.45 (24.19 to 48.43) | -1.78 (-1.88 to -1.68) |
| Costa Rica | 91 (59 to 125) | 5.57 (3.43 to 7.8) | 285 (147 to 411) | 4.99 (2.63 to 7.12) | -1.41 (-1.85 to -0.96) |
| Croatia | 745 (404 to 1080) | 14.68 (7.53 to 21.74) | 752 (339 to 1103) | 7.74 (3.74 to 11.21) | -1.06 (-1.51 to -0.6) |
| Cuba | 244 (184 to 315) | 2.46 (1.82 to 3.23) | 1617 (1002 to 2254) | 7.84 (5.06 to 10.72) | 4.32 (4.12 to 4.53) |
| Cyprus | 51 (20 to 94) | 10.91 (3.11 to 22.1) | 101 (41 to 162) | 7.2 (2.32 to 11.94) | -1.45 (-1.8 to -1.1) |
| Czechia | 298 (213 to 380) | 2.21 (1.56 to 2.83) | 1302 (689 to 1868) | 5.58 (2.98 to 7.97) | 2.81 (1.95 to 3.69) |
| Côte d'Ivoire | 386 (210 to 551) | 11.11 (5.77 to 16.35) | 1534 (857 to 2217) | 15.38 (8.21 to 22.68) | 1.06 (0.81 to 1.31) |
| Democratic People's Republic of Korea | 763 (407 to 1247) | 6.67 (3.09 to 11.57) | 2877 (1593 to 4573) | 10.34 (5.43 to 17.02) | 1.65 (1.54 to 1.77) |
| Democratic Republic of the Congo | 1963 (858 to 3121) | 15.15 (6.72 to 24.07) | 7562 (4059 to 11812) | 27.04 (13.58 to 43.96) | 1.92 (1.85 to 1.99) |
| Denmark | 97 (52 to 142) | 1.12 (0.61 to 1.62) | 142 (54 to 224) | 1.02 (0.42 to 1.58) | -0.27 (-0.57 to 0.02) |
| Djibouti | 13 (8 to 20) | 12.14 (7.01 to 19.4) | 55 (33 to 89) | 11.28 (5.89 to 19.64) | -0.45 (-0.53 to -0.37) |
| Dominica | 18 (11 to 24) | 30.71 (18.93 to 41.73) | 24 (15 to 33) | 30.76 (18.2 to 42.31) | 0.14 (-0.02 to 0.31) |
| Dominican Republic | 260 (183 to 362) | 7.89 (4.96 to 11.57) | 872 (530 to 1282) | 8.89 (5.34 to 13.28) | 0.94 (0.73 to 1.15) |
| Ecuador | 478 (323 to 652) | 10.2 (5.92 to 14.45) | 1051 (556 to 1593) | 7.32 (3.52 to 11.22) | 0.41 (-0.44 to 1.26) |
| Egypt | 9908 (6765 to 14098) | 52.77 (28.54 to 82.63) | 18827 (13394 to 25036) | 42.72 (25 to 60.52) | -0.33 (-0.49 to -0.17) |
| El Salvador | 104 (68 to 144) | 3.65 (2.3 to 5.12) | 207 (110 to 317) | 3.05 (1.71 to 4.54) | -0.59 (-0.81 to -0.37) |
| Equatorial Guinea | 49 (26 to 79) | 28.87 (15.7 to 45.88) | 103 (53 to 170) | 25.79 (12.44 to 46.01) | -0.65 (-0.96 to -0.33) |
| Eritrea | 128 (54 to 199) | 13.87 (6.27 to 21.98) | 307 (168 to 454) | 14.36 (7.24 to 23.7) | 0.09 (-0.02 to 0.21) |
| Estonia | 133 (97 to 166) | 6.72 (4.86 to 8.42) | 1273 (551 to 1845) | 38.87 (19.96 to 54.6) | 8.19 (7.09 to 9.31) |
| Eswatini | 82 (50 to 112) | 36.14 (20.35 to 52.03) | 190 (99 to 289) | 42.48 (22.43 to 64.28) | 0.99 (0.53 to 1.44) |
| Ethiopia | 2414 (1179 to 3457) | 13.91 (7.08 to 20.64) | 2888 (1829 to 4285) | 7.54 (4.35 to 12.04) | -2.55 (-2.76 to -2.34) |
| Fiji | 58 (44 to 76) | 17.73 (12.32 to 24.05) | 109 (78 to 145) | 16.6 (10.87 to 22.68) | -0.5 (-0.68 to -0.33) |
| Finland | 217 (121 to 315) | 3.04 (1.66 to 4.43) | 1057 (377 to 1635) | 6.49 (2.76 to 9.65) | 3.6 (2.87 to 4.33) |
| France | 2384 (892 to 3846) | 2.69 (1.05 to 4.27) | 4384 (1239 to 7303) | 2 (0.7 to 3.18) | -0.86 (-1.01 to -0.71) |
| Gabon | 166 (95 to 243) | 32.8 (17.7 to 49.79) | 287 (162 to 451) | 35.02 (18.98 to 58.78) | 0.06 (-0.1 to 0.22) |
| Gambia | 38 (24 to 55) | 11.86 (7.02 to 17.99) | 167 (92 to 249) | 18.3 (9.88 to 27.85) | 1.26 (1.06 to 1.46) |
| Georgia | 407 (293 to 549) | 6.93 (4.81 to 9.48) | 1526 (776 to 2159) | 23.52 (13.24 to 32.44) | 6.67 (5.37 to 7.99) |
| Germany | 10032 (5081 to 14664) | 7.54 (3.89 to 10.97) | 15953 (4783 to 25532) | 6.21 (2.14 to 9.66) | 0.49 (0.11 to 0.87) |
| Ghana | 728 (444 to 1062) | 12.14 (7.58 to 18.31) | 2345 (1392 to 3344) | 15.77 (9.06 to 23.46) | 0.55 (0.31 to 0.79) |
| Greece | 494 (230 to 753) | 3.58 (1.51 to 5.61) | 1698 (527 to 2664) | 4.92 (1.93 to 7.37) | 1.76 (1.21 to 2.32) |
| Greenland | 2 (1 to 2) | 6.14 (3.96 to 8.57) | 2 (2 to 3) | 3.78 (2.52 to 5.12) | -1.3 (-1.52 to -1.08) |
| Grenada | 8 (6 to 11) | 11.47 (8.47 to 14.82) | 18 (13 to 23) | 17.43 (11.71 to 23.22) | 1.86 (1.6 to 2.12) |
| Guam | 15 (9 to 19) | 23.59 (13.21 to 32.99) | 14 (11 to 22) | 6.85 (5.25 to 10.48) | -3.91 (-4.56 to -3.26) |
| Guatemala | 104 (77 to 130) | 4.1 (2.45 to 5.6) | 240 (143 to 334) | 2.68 (1.38 to 3.81) | -0.76 (-1.25 to -0.27) |
| Guinea | 343 (177 to 520) | 10.8 (5.51 to 16.81) | 726 (387 to 1068) | 13.66 (7.34 to 20.98) | 0.77 (0.57 to 0.96) |
| Guinea-Bissau | 62 (29 to 96) | 16.22 (7.69 to 25.92) | 143 (69 to 221) | 21.16 (10.06 to 33.13) | 0.84 (0.66 to 1.02) |
| Guyana | 126 (95 to 162) | 36.07 (25.43 to 47.33) | 199 (144 to 266) | 33.71 (23 to 46.64) | 0.43 (0.07 to 0.79) |
| Haiti | 298 (108 to 514) | 10.09 (3.78 to 17.51) | 786 (352 to 1291) | 11.87 (5.03 to 20.65) | 0.64 (0.59 to 0.7) |
| Honduras | 202 (141 to 297) | 11.36 (7.02 to 17.91) | 875 (598 to 1243) | 16.44 (9.86 to 25.21) | 1.34 (1.15 to 1.53) |
| Hungary | 2288 (1478 to 3026) | 16.68 (9.91 to 22.57) | 3598 (1969 to 4965) | 16.9 (9.68 to 22.93) | 1.03 (0.55 to 1.52) |
| Iceland | 5 (2 to 7) | 1.59 (0.78 to 2.34) | 12 (4 to 19) | 1.68 (0.7 to 2.53) | 1.11 (0.77 to 1.44) |
| India | 10367 (5646 to 15883) | 2.61 (1.33 to 4.35) | 45931 (29768 to 67057) | 4.35 (2.56 to 6.74) | 1.89 (1.74 to 2.05) |
| Indonesia | 5476 (2815 to 8194) | 5.96 (2.95 to 9.28) | 19834 (11647 to 28220) | 9.29 (5.22 to 13.91) | 1.55 (1.41 to 1.68) |
| Iran (Islamic Republic of) | 3216 (2328 to 4403) | 16.2 (9.72 to 24.6) | 10994 (6671 to 14834) | 16.48 (9.17 to 22.89) | 0.43 (0.26 to 0.61) |
| Iraq | 1562 (859 to 2246) | 21.25 (11.52 to 31) | 3615 (2331 to 4923) | 20.54 (11.11 to 28.94) | -0.66 (-0.83 to -0.49) |
| Ireland | 60 (31 to 88) | 1.61 (0.75 to 2.43) | 97 (38 to 151) | 1.13 (0.46 to 1.74) | -0.11 (-0.37 to 0.14) |
| Israel | 116 (63 to 168) | 2.62 (1.29 to 3.9) | 177 (67 to 277) | 1.22 (0.5 to 1.86) | -2.52 (-3.29 to -1.76) |
| Italy | 5033 (2525 to 7599) | 5.76 (2.73 to 8.83) | 15577 (4617 to 25734) | 7.32 (2.57 to 11.66) | 0.95 (0.83 to 1.07) |
| Jamaica | 325 (206 to 453) | 17.74 (11.51 to 24.49) | 575 (361 to 810) | 17.39 (11.35 to 24.15) | 0.87 (0.18 to 1.57) |
| Japan | 3691 (1742 to 5851) | 2.53 (1.03 to 4.17) | 4836 (1440 to 8578) | 0.84 (0.36 to 1.35) | -3.14 (-4.07 to -2.21) |
| Jordan | 334 (225 to 457) | 32.55 (18.83 to 46.57) | 1296 (858 to 1723) | 24.41 (13.7 to 34.49) | -1.04 (-1.24 to -0.83) |
| Kazakhstan | 637 (495 to 781) | 5.31 (3.87 to 6.63) | 780 (545 to 1031) | 5.25 (3.34 to 7.14) | -1.13 (-2.81 to 0.58) |
| Kenya | 643 (408 to 915) | 9.04 (5.35 to 13.81) | 2509 (1518 to 3508) | 14.1 (7.37 to 21.83) | 1.9 (1.72 to 2.07) |
| Kiribati | 3 (2 to 4) | 8.65 (5.64 to 11.62) | 6 (4 to 8) | 9 (5.61 to 12.22) | 0.09 (0.07 to 0.11) |
| Kuwait | 108 (82 to 134) | 22.71 (14.35 to 30.72) | 247 (141 to 356) | 11.22 (5.77 to 16.4) | -2.16 (-2.5 to -1.82) |
| Kyrgyzstan | 165 (119 to 215) | 5.92 (4.11 to 7.95) | 422 (300 to 552) | 10.21 (6.7 to 13.68) | 1.39 (1.02 to 1.75) |
| Lao People's Democratic Republic | 212 (74 to 348) | 11.12 (3.92 to 19.27) | 433 (247 to 623) | 10.27 (5.73 to 15.24) | -0.27 (-0.33 to -0.21) |
| Latvia | 60 (44 to 74) | 1.71 (1.25 to 2.13) | 529 (273 to 734) | 11.67 (6.73 to 15.63) | 8.46 (7.23 to 9.71) |
| Lebanon | 281 (106 to 469) | 15.71 (5.72 to 27.24) | 492 (236 to 729) | 7.38 (3.82 to 10.77) | -2.77 (-2.95 to -2.59) |
| Lesotho | 189 (119 to 268) | 25.64 (15.52 to 39.17) | 389 (204 to 567) | 43.12 (21.12 to 64.86) | 2.57 (2.06 to 3.08) |
| Liberia | 165 (101 to 236) | 15.07 (9.15 to 22.03) | 415 (223 to 633) | 20.92 (11.06 to 32.76) | 0.99 (0.84 to 1.13) |
| Libya | 253 (137 to 391) | 14.91 (7.88 to 23.52) | 1036 (555 to 1610) | 23.79 (12.04 to 37.29) | 2 (1.84 to 2.16) |
| Lithuania | 86 (62 to 110) | 1.94 (1.38 to 2.49) | 410 (229 to 560) | 6.41 (4.07 to 8.41) | 4.87 (4.19 to 5.55) |
| Luxembourg | 14 (7 to 22) | 2.82 (1.24 to 4.32) | 35 (12 to 54) | 2.7 (1.01 to 4.1) | 0.35 (0.12 to 0.59) |
| Madagascar | 857 (526 to 1255) | 19.08 (11.47 to 29.87) | 2457 (1517 to 3653) | 27.12 (15.71 to 41.7) | 1.05 (0.97 to 1.12) |
| Malawi | 300 (131 to 464) | 9.42 (3.95 to 15.21) | 794 (410 to 1166) | 12.76 (6.4 to 19.95) | 0.75 (0.6 to 0.9) |
| Malaysia | 271 (173 to 369) | 3.07 (1.88 to 4.29) | 691 (496 to 912) | 2.65 (1.75 to 3.73) | -1.13 (-1.42 to -0.83) |
| Maldives | 4 (1 to 7) | 4.78 (1.64 to 7.95) | 9 (6 to 13) | 2.93 (1.86 to 4.46) | -1.9 (-2.12 to -1.69) |
| Mali | 391 (145 to 603) | 10.53 (3.99 to 16.41) | 851 (373 to 1302) | 10.15 (4.41 to 15.68) | -0.16 (-0.22 to -0.1) |
| Malta | 10 (6 to 15) | 2.76 (1.36 to 4.12) | 31 (12 to 47) | 2.69 (1.17 to 4.03) | 0.65 (0.35 to 0.96) |
| Marshall Islands | 3 (2 to 5) | 22.75 (13.53 to 32.93) | 7 (4 to 10) | 21.05 (12.69 to 30.3) | -0.35 (-0.44 to -0.25) |
| Mauritania | 204 (116 to 307) | 22.05 (12.25 to 33.48) | 412 (231 to 655) | 21.39 (11.12 to 34.38) | -0.26 (-0.36 to -0.17) |
| Mauritius | 109 (83 to 137) | 16.22 (11.7 to 21.52) | 218 (151 to 291) | 12.73 (8.26 to 17.58) | -0.49 (-1.13 to 0.14) |
| Mexico | 2051 (1258 to 2842) | 6.13 (3.21 to 8.95) | 5059 (2919 to 6926) | 4.5 (2.39 to 6.26) | -0.77 (-1.05 to -0.49) |
| Micronesia (Federated States of) | 11 (6 to 16) | 24.01 (13.67 to 33.73) | 15 (9 to 20) | 21.38 (12.92 to 29.61) | -0.43 (-0.46 to -0.39) |
| Monaco | 2 (1 to 4) | 3.02 (1.3 to 5.03) | 5 (2 to 8) | 3.67 (1.49 to 5.98) | 0.83 (0.4 to 1.27) |
| Mongolia | 61 (36 to 93) | 6.4 (3.7 to 10.06) | 82 (51 to 125) | 4.49 (2.4 to 7.17) | -1.68 (-1.92 to -1.43) |
| Montenegro | 29 (16 to 42) | 4.96 (2.71 to 7.34) | 64 (35 to 97) | 7.56 (3.79 to 11.73) | 1.52 (1.32 to 1.72) |
| Morocco | 2789 (1467 to 4249) | 21.98 (11.61 to 34.85) | 7418 (4276 to 11151) | 25.18 (13.48 to 39.38) | 0.71 (0.56 to 0.86) |
| Mozambique | 741 (408 to 1069) | 14.87 (8.03 to 21.87) | 2047 (1150 to 3209) | 22.43 (11.81 to 37.13) | 1.78 (1.59 to 1.98) |
| Myanmar | 2116 (773 to 3417) | 9.49 (3.44 to 15.29) | 3567 (1747 to 5467) | 7.7 (3.61 to 12.09) | -1.03 (-1.15 to -0.9) |
| Namibia | 123 (81 to 174) | 23.79 (13.65 to 36.44) | 346 (216 to 509) | 32.17 (18 to 49.6) | 0.79 (0.45 to 1.13) |
| Nauru | 1 (1 to 2) | 23.76 (11.59 to 35.25) | 1 (1 to 2) | 22.32 (11.34 to 33.87) | -0.31 (-0.56 to -0.05) |
| Nepal | 257 (129 to 410) | 2.97 (1.46 to 4.92) | 944 (615 to 1383) | 4.48 (2.77 to 6.94) | 1.64 (1.24 to 2.04) |
| Netherlands | 210 (106 to 315) | 1.04 (0.52 to 1.56) | 571 (177 to 926) | 1.39 (0.46 to 2.22) | 1.37 (1.08 to 1.66) |
| New Zealand | 76 (49 to 105) | 2.05 (1.25 to 2.89) | 110 (50 to 161) | 1.19 (0.6 to 1.7) | -2.12 (-2.44 to -1.79) |
| Nicaragua | 89 (58 to 121) | 6.69 (4 to 9.51) | 249 (155 to 356) | 5.91 (3.37 to 8.61) | -0.46 (-0.65 to -0.27) |
| Niger | 223 (77 to 359) | 9.05 (2.87 to 15.23) | 704 (232 to 1139) | 9.35 (3.07 to 15.41) | -0.02 (-0.18 to 0.14) |
| Nigeria | 4639 (2829 to 7001) | 12.04 (6.91 to 18.32) | 9083 (5167 to 13061) | 11.76 (6.6 to 17.55) | -0.51 (-0.73 to -0.29) |
| Niue | 0 (0 to 1) | 15.97 (10.06 to 22.37) | 0 (0 to 0) | 14.57 (9.22 to 20.04) | -0.47 (-0.52 to -0.42) |
| North Macedonia | 313 (180 to 459) | 19.9 (10.62 to 30.15) | 571 (327 to 874) | 25.01 (10.33 to 40.14) | 0.89 (0.46 to 1.32) |
| Northern Mariana Islands | 1 (1 to 1) | 4.76 (3.07 to 6.65) | 2 (2 to 2) | 4.47 (2.88 to 5.83) | -0.12 (-0.25 to 0.02) |
| Norway | 89 (42 to 136) | 1.17 (0.57 to 1.76) | 116 (40 to 192) | 0.9 (0.34 to 1.45) | -0.41 (-0.97 to 0.16) |
| Oman | 122 (77 to 194) | 21.46 (12.17 to 34.92) | 370 (247 to 503) | 26.71 (15.22 to 37.67) | 1.6 (1.1 to 2.1) |
| Pakistan | 2699 (1439 to 4052) | 5.48 (2.84 to 8.53) | 8927 (5743 to 13254) | 8.93 (5.27 to 13.88) | 1.49 (1.18 to 1.81) |
| Palau | 1 (0 to 1) | 6.36 (4.39 to 8.88) | 1 (1 to 1) | 5.54 (3.81 to 7.6) | -0.36 (-0.42 to -0.31) |
| Palestine | 223 (129 to 344) | 31.54 (16.3 to 49.88) | 416 (262 to 562) | 24.12 (12.35 to 34.76) | -0.82 (-1.02 to -0.62) |
| Panama | 44 (28 to 59) | 3.24 (1.92 to 4.38) | 222 (119 to 314) | 4.84 (2.67 to 6.73) | 0.96 (0.42 to 1.49) |
| Papua New Guinea | 148 (73 to 238) | 8 (4.12 to 12.76) | 398 (217 to 670) | 7.4 (4.09 to 12.49) | -0.31 (-0.35 to -0.28) |
| Paraguay | 169 (110 to 237) | 8.19 (4.96 to 11.76) | 543 (326 to 774) | 10.02 (5.7 to 14.42) | 0.94 (0.82 to 1.06) |
| Peru | 411 (260 to 587) | 3.69 (2.16 to 5.42) | 862 (460 to 1326) | 2.56 (1.37 to 3.92) | -1.41 (-1.71 to -1.11) |
| Philippines | 2459 (1841 to 3168) | 9.17 (6.37 to 13.15) | 10203 (7272 to 13304) | 13.31 (9 to 17.91) | 1.42 (1.3 to 1.54) |
| Poland | 2959 (2018 to 3845) | 7.21 (4.63 to 9.53) | 6180 (3201 to 8565) | 8 (4.43 to 10.94) | 0.24 (-0.04 to 0.52) |
| Portugal | 420 (212 to 629) | 3.57 (1.5 to 5.62) | 1068 (302 to 1749) | 3.19 (1.07 to 5.06) | -0.3 (-0.47 to -0.13) |
| Puerto Rico | 258 (183 to 330) | 7.49 (5.15 to 9.81) | 554 (290 to 795) | 6.61 (4.23 to 8.99) | -0.06 (-0.41 to 0.3) |
| Qatar | 11 (7 to 14) | 18.31 (9.56 to 26.81) | 40 (27 to 58) | 9.18 (4.68 to 13.45) | -2.61 (-3.18 to -2.04) |
| Republic of Korea | 885 (487 to 1291) | 4.46 (1.9 to 7.27) | 1644 (495 to 3078) | 1.89 (0.56 to 3.59) | -2.71 (-2.97 to -2.45) |
| Republic of Moldova | 116 (92 to 139) | 2.87 (2.1 to 3.58) | 1174 (765 to 1554) | 19.39 (12.75 to 25.69) | 7.24 (6.83 to 7.66) |
| Romania | 5264 (3559 to 6988) | 21.67 (12.79 to 30.33) | 8390 (4041 to 11997) | 20.39 (10.44 to 28.56) | 0.44 (0.07 to 0.82) |
| Russian Federation | 3659 (2875 to 4376) | 2.13 (1.61 to 2.61) | 9038 (5879 to 11708) | 3.78 (2.5 to 4.88) | 1.75 (0.44 to 3.07) |
| Rwanda | 452 (102 to 717) | 18.59 (4.43 to 29.77) | 608 (153 to 963) | 12.44 (3.35 to 21.62) | -2.23 (-2.6 to -1.85) |
| Saint Kitts and Nevis | 5 (3 to 6) | 13.46 (8.9 to 17.93) | 10 (7 to 13) | 17.1 (10.07 to 23.31) | 1.81 (1.34 to 2.27) |
| Saint Lucia | 14 (9 to 18) | 17.96 (10.55 to 25.73) | 37 (22 to 52) | 15.87 (8.99 to 22.88) | -0.23 (-0.75 to 0.29) |
| Saint Vincent and the Grenadines | 11 (8 to 15) | 16.34 (10.6 to 23.06) | 34 (23 to 46) | 26.32 (16.3 to 36.8) | 1.62 (1.05 to 2.19) |
| Samoa | 16 (10 to 21) | 19.88 (12.46 to 27.84) | 23 (15 to 31) | 17.16 (10.82 to 23.51) | -0.51 (-0.64 to -0.38) |
| San Marino | 2 (1 to 3) | 3.99 (1.64 to 6.66) | 3 (1 to 5) | 2.53 (1 to 4.1) | -0.34 (-0.74 to 0.07) |
| Sao Tome and Principe | 5 (3 to 6) | 7.75 (5.21 to 10.55) | 9 (6 to 13) | 9.2 (5.89 to 13.23) | 0.43 (0.31 to 0.55) |
| Saudi Arabia | 1529 (879 to 2358) | 32.22 (16.86 to 50.08) | 4582 (2411 to 6872) | 33.52 (15.82 to 50.48) | -0.07 (-0.21 to 0.08) |
| Senegal | 329 (213 to 461) | 10.84 (6.96 to 15.62) | 1011 (591 to 1458) | 14.19 (8.05 to 20.96) | 0.88 (0.79 to 0.97) |
| Serbia | 1409 (782 to 2156) | 17.64 (8.58 to 27.89) | 2562 (1258 to 3813) | 14.76 (7.16 to 22.04) | -0.89 (-1.07 to -0.71) |
| Seychelles | 19 (13 to 25) | 33.01 (23.64 to 44.2) | 30 (21 to 40) | 28.42 (18.31 to 39.92) | -0.44 (-0.6 to -0.29) |
| Sierra Leone | 168 (97 to 248) | 8.81 (5.07 to 13.66) | 424 (225 to 642) | 12.06 (6.27 to 18.47) | 1.11 (0.86 to 1.37) |
| Singapore | 81 (57 to 109) | 4.12 (2.5 to 6) | 232 (151 to 317) | 2.73 (1.75 to 3.78) | -1.04 (-1.44 to -0.65) |
| Slovakia | 383 (239 to 598) | 6.64 (3.93 to 10.76) | 685 (368 to 1023) | 7.18 (3.83 to 10.75) | 0.81 (0.54 to 1.07) |
| Slovenia | 223 (133 to 299) | 9.15 (5.5 to 12.43) | 572 (177 to 894) | 9.83 (3.49 to 15.1) | 0.94 (0.64 to 1.23) |
| Solomon Islands | 14 (7 to 21) | 10.38 (5.38 to 15.86) | 36 (22 to 55) | 10.63 (6.08 to 15.89) | 0.1 (0.05 to 0.15) |
| Somalia | 363 (159 to 573) | 16.59 (7.68 to 25.43) | 817 (340 to 1323) | 15.98 (6.94 to 26.04) | -0.12 (-0.24 to 0) |
| South Africa | 3539 (2431 to 4875) | 18.67 (11.76 to 26.97) | 11034 (7725 to 14190) | 28.15 (17.06 to 38.09) | 1.38 (0.91 to 1.87) |
| South Sudan | 214 (99 to 344) | 9.56 (4.12 to 15.76) | 288 (123 to 444) | 8.73 (3.58 to 13.77) | -0.5 (-0.86 to -0.14) |
| Spain | 1371 (631 to 2042) | 2.65 (1.13 to 4.02) | 5113 (1504 to 8053) | 3.52 (1.26 to 5.37) | 1.08 (0.91 to 1.25) |
| Sri Lanka | 768 (533 to 1036) | 8 (4.98 to 11.58) | 1517 (866 to 2671) | 5.97 (3.23 to 10.68) | -1.11 (-1.31 to -0.91) |
| Sudan | 2297 (1176 to 3506) | 28.5 (14.76 to 43.33) | 4672 (2629 to 7207) | 28.7 (15.27 to 44.64) | 0.04 (-0.11 to 0.18) |
| Suriname | 25 (17 to 34) | 10.15 (6.82 to 14.29) | 62 (41 to 91) | 10.12 (6.49 to 15.11) | 0.29 (0.12 to 0.46) |
| Sweden | 141 (62 to 220) | 0.83 (0.38 to 1.29) | 858 (261 to 1416) | 2.9 (0.98 to 4.68) | 5.53 (4.93 to 6.14) |
| Switzerland | 410 (162 to 647) | 3.62 (1.51 to 5.63) | 1047 (311 to 1785) | 3.99 (1.41 to 6.59) | 0.86 (0.46 to 1.27) |
| Syrian Arab Republic | 1052 (644 to 1462) | 24.5 (13.56 to 35.96) | 2327 (1410 to 3440) | 24.78 (12.68 to 36.81) | -0.42 (-0.71 to -0.12) |
| Taiwan (Province of China) | 893 (635 to 1188) | 7.18 (4.3 to 10.67) | 2190 (1051 to 3298) | 4.92 (2.52 to 7.23) | -0.03 (-0.84 to 0.78) |
| Tajikistan | 531 (294 to 904) | 21.74 (11.24 to 38.73) | 769 (482 to 1106) | 17.72 (9.29 to 26.41) | -0.91 (-1.32 to -0.5) |
| Thailand | 363 (225 to 521) | 1.14 (0.64 to 1.77) | 1441 (890 to 2200) | 1.33 (0.82 to 2.04) | 0.13 (-0.02 to 0.28) |
| Timor-Leste | 12 (5 to 20) | 4.88 (2.1 to 8.37) | 47 (28 to 70) | 5.81 (3.43 to 8.97) | 0.71 (0.44 to 0.99) |
| Togo | 131 (79 to 193) | 11.49 (6.63 to 18.04) | 570 (294 to 874) | 17.18 (8.93 to 26.42) | 1.34 (1.15 to 1.52) |
| Tokelau | 0 (0 to 0) | 18.55 (11.93 to 26.58) | 0 (0 to 0) | 13.15 (8.28 to 18.69) | -1.2 (-1.23 to -1.16) |
| Tonga | 3 (2 to 4) | 4.95 (3.21 to 6.83) | 4 (3 to 5) | 5.07 (3.4 to 6.89) | 0.06 (-0.09 to 0.22) |
| Trinidad and Tobago | 139 (99 to 181) | 19.13 (12.1 to 26.99) | 204 (136 to 287) | 10.9 (7.07 to 15.42) | -2 (-2.27 to -1.73) |
| Tunisia | 702 (442 to 1022) | 17.47 (9.79 to 26.77) | 2471 (1162 to 4552) | 21.1 (9.45 to 39.42) | 0.68 (0.62 to 0.75) |
| Turkmenistan | 149 (95 to 206) | 8.68 (5.15 to 12.5) | 406 (261 to 611) | 11.39 (6.98 to 17.47) | 0.55 (0.31 to 0.8) |
| Tuvalu | 1 (1 to 2) | 22.83 (13.25 to 32.38) | 2 (1 to 2) | 17.5 (11.36 to 24.1) | -0.83 (-0.9 to -0.76) |
| Turkey | 5355 (2828 to 7866) | 19.91 (9.41 to 30.03) | 12264 (6190 to 17883) | 15.21 (6.84 to 22.63) | -0.6 (-1.06 to -0.14) |
| Uganda | 465 (138 to 775) | 8.32 (2.55 to 14.09) | 1186 (447 to 1944) | 9.71 (3.49 to 17.74) | 0.02 (-0.17 to 0.21) |
| Ukraine | 2270 (1670 to 2890) | 3.31 (2.31 to 4.33) | 3330 (2030 to 4840) | 4.28 (2.67 to 6.15) | 0.55 (0.27 to 0.83) |
| United Arab Emirates | 57 (37 to 82) | 18.07 (9.85 to 28.25) | 346 (246 to 462) | 21.2 (12.2 to 29.33) | 2.43 (1.75 to 3.12) |
| United Kingdom | 1520 (991 to 2020) | 1.65 (1.1 to 2.16) | 2665 (1393 to 3698) | 1.86 (1.1 to 2.48) | 0.96 (0.78 to 1.13) |
| United Republic of Tanzania | 1404 (617 to 2123) | 15.94 (6.21 to 25.71) | 3699 (1540 to 5732) | 18.12 (7 to 29.89) | 0.41 (0.35 to 0.48) |
| United States of America | 13299 (8979 to 17356) | 4.2 (2.95 to 5.38) | 43431 (27559 to 56335) | 7.44 (5.13 to 9.4) | 2.09 (1.94 to 2.24) |
| United States Virgin Islands | 12 (8 to 16) | 17 (10.55 to 24.2) | 16 (10 to 24) | 9.75 (5.48 to 14.34) | -1.39 (-1.55 to -1.23) |
| Uruguay | 177 (113 to 238) | 4.55 (2.82 to 6.19) | 436 (189 to 643) | 6.47 (3.34 to 9.17) | 1.05 (0.92 to 1.17) |
| Uzbekistan | 786 (499 to 1155) | 7.27 (4.54 to 10.84) | 2525 (1716 to 3551) | 11.34 (7.19 to 16.28) | 1.69 (1.44 to 1.94) |
| Vanuatu | 7 (4 to 10) | 11.54 (6.63 to 17.17) | 21 (14 to 29) | 12.44 (7.97 to 17.1) | 0.18 (0.13 to 0.24) |
| Venezuela (Bolivarian Republic of) | 1380 (987 to 1788) | 15.98 (10.78 to 21.44) | 3354 (1953 to 4864) | 11.96 (6.53 to 17.62) | -1.3 (-1.63 to -0.98) |
| Viet Nam | 1453 (796 to 2405) | 3.94 (2.07 to 6.81) | 4145 (2208 to 6678) | 4.69 (2.41 to 7.93) | 0.87 (0.66 to 1.07) |
| Yemen | 1023 (495 to 1568) | 25.45 (12.14 to 41.07) | 3614 (1866 to 6085) | 31.93 (15.18 to 54.51) | 0.56 (0.37 to 0.74) |
| Zambia | 454 (267 to 637) | 18.38 (10.73 to 26.58) | 1356 (694 to 1994) | 24.44 (12.11 to 37.63) | 0.67 (0.54 to 0.8) |
| Zimbabwe | 410 (286 to 578) | 12.07 (7.63 to 18.72) | 1405 (879 to 2088) | 23.98 (14.67 to 36.18) | 3 (2.47 to 3.54) |
