# Supplementary material for: Global burden of hypertensive heart disease attributable to high body mass index from 1990 to 2021: a multidimensional analysis and public health response
Source: Front Cardiovasc Med. 2025 Aug 12;12:1570390. doi: 10.3389/fcvm.2025.1570390 (PMC12379062; doi:10.3389/fcvm.2025.1570390)
Supplement: Supplementary file 5 [file Table5.docx]

Supplementary Table S5 DALYs and ASDR of hypertensive heart disease attributable to high BMI in 204 countries and territories in 1990 and 2021, and the temporal trends from 1990 to 2021.

| **DALY** | 1990 | | 2021 | | 1990–2021 |
| --- | --- | --- | --- | --- | --- |
| Location | DALYs cases  No. (95% UI) | ASDR per 100,000  No. (95% UI) | DALYs cases  No. (95% UI) | ASDR per 100,000  No. (95% UI) | EAPC in ASDR  No. (95% CI) |
| Afghanistan | 61151 (21259 to 104982) | 865.66 (315.13 to 1466.02) | 83305 (38782 to 135678) | 802.84 (387.04 to 1302.15) | -0.37 (-0.51 to -0.23) |
| Albania | 2392 (1521 to 3436) | 129.1 (79.12 to 189.37) | 3921 (2160 to 6239) | 92.36 (49.77 to 146.75) | -0.65 (-0.88 to -0.42) |
| Algeria | 53557 (34556 to 79684) | 482.37 (289.02 to 728.78) | 145616 (89971 to 210695) | 459.09 (268.54 to 676.13) | 0.08 (-0.04 to 0.2) |
| American Samoa | 70 (51 to 87) | 278.83 (198.62 to 349.45) | 111 (86 to 141) | 219.9 (168.73 to 278.56) | -1 (-1.27 to -0.74) |
| Andorra | 40 (24 to 61) | 79.97 (42.73 to 125.59) | 92 (45 to 151) | 53.72 (28.77 to 87.03) | -0.9 (-1.08 to -0.72) |
| Angola | 18802 (9617 to 28056) | 453.9 (244.09 to 676.16) | 57503 (36082 to 83904) | 480.53 (299.48 to 700.29) | -0.08 (-0.19 to 0.02) |
| Antigua and Barbuda | 185 (139 to 235) | 350.21 (272.03 to 435.79) | 509 (405 to 613) | 486.53 (370.6 to 600.67) | 1.46 (1 to 1.92) |
| Argentina | 55802 (43117 to 68643) | 176.22 (132.8 to 220.16) | 79771 (53240 to 103843) | 139.87 (95.04 to 180.37) | -0.44 (-0.61 to -0.28) |
| Armenia | 4511 (3257 to 5859) | 180.01 (127.01 to 237.4) | 6286 (4068 to 8709) | 145.55 (95.58 to 201.08) | -0.34 (-0.65 to -0.04) |
| Australia | 5140 (3611 to 6737) | 27.3 (18.79 to 36.45) | 11621 (7093 to 15466) | 24.76 (16.42 to 31.56) | -0.05 (-0.49 to 0.38) |
| Austria | 9465 (5952 to 12650) | 79.21 (52.2 to 104.29) | 17159 (7301 to 25909) | 78.65 (40.8 to 113.33) | 0.8 (0.49 to 1.11) |
| Azerbaijan | 13916 (9706 to 18930) | 286.27 (193.48 to 395.45) | 22173 (14661 to 32323) | 228.3 (147.87 to 332.85) | -0.55 (-0.81 to -0.29) |
| Bahamas | 1122 (918 to 1330) | 680.09 (540.23 to 820.54) | 3568 (2701 to 4476) | 851.26 (632.91 to 1078.31) | 0.98 (0.77 to 1.19) |
| Bahrain | 491 (370 to 625) | 326.77 (217.89 to 440.03) | 1706 (1208 to 2366) | 249.19 (149.96 to 358.19) | -1.24 (-1.43 to -1.04) |
| Bangladesh | 35525 (20846 to 54108) | 73.92 (42.8 to 113.7) | 130349 (75491 to 257675) | 96.18 (53.84 to 183.83) | 1.17 (0.98 to 1.36) |
| Barbados | 510 (369 to 650) | 181.4 (136.37 to 226.03) | 991 (712 to 1327) | 196.58 (141.24 to 263.64) | 0.82 (0.66 to 0.97) |
| Belarus | 8400 (6172 to 10907) | 64.62 (48.15 to 82.59) | 2558 (1969 to 3288) | 16.97 (13.09 to 21.78) | -5.4 (-6.54 to -4.24) |
| Belgium | 2741 (1603 to 3876) | 17.94 (10.72 to 25.17) | 4377 (2078 to 6474) | 15.9 (9.08 to 21.91) | -0.43 (-0.9 to 0.05) |
| Belize | 216 (174 to 260) | 225.9 (180 to 273.85) | 1106 (916 to 1326) | 356.34 (277.77 to 439.27) | 1.92 (1.67 to 2.17) |
| Benin | 4717 (2818 to 6553) | 225.1 (133.59 to 314.81) | 14812 (8804 to 21058) | 258.9 (152.65 to 365.51) | 0.44 (0.32 to 0.56) |
| Bermuda | 65 (50 to 80) | 105.37 (79.24 to 132.91) | 143 (97 to 189) | 104.76 (75.09 to 134.87) | 0.4 (-0.16 to 0.95) |
| Bhutan | 419 (203 to 632) | 166.95 (78.95 to 258.7) | 838 (538 to 1221) | 139.12 (88.89 to 204.39) | -0.66 (-0.7 to -0.62) |
| Bolivia (Plurinational State of) | 5184 (2280 to 7818) | 160.07 (68.94 to 244.42) | 12772 (7210 to 19100) | 144.71 (79.27 to 216.13) | -0.36 (-0.4 to -0.32) |
| Bosnia and Herzegovina | 5342 (3849 to 7327) | 137.32 (95.47 to 194.1) | 8368 (5117 to 12215) | 132.67 (81.87 to 192.99) | 0.09 (-0.09 to 0.27) |
| Botswana | 2780 (1821 to 4072) | 504.37 (327.36 to 749.41) | 7020 (4903 to 10010) | 497.27 (340.74 to 698.25) | 0.29 (-0.02 to 0.6) |
| Brazil | 213068 (175781 to 252165) | 232.92 (184.52 to 283.37) | 355515 (277792 to 428607) | 142.2 (109.04 to 173.05) | -1.54 (-1.68 to -1.41) |
| Brunei Darussalam | 135 (93 to 178) | 110.82 (73.2 to 154.36) | 348 (264 to 472) | 94.64 (65 to 131.26) | -0.35 (-0.54 to -0.16) |
| Bulgaria | 49422 (39526 to 60558) | 429.9 (324.17 to 535.84) | 149848 (105423 to 194684) | 1083.92 (779.93 to 1396.41) | 3.84 (3.11 to 4.57) |
| Burkina Faso | 8468 (5071 to 12582) | 181.65 (106.43 to 271.5) | 24698 (13079 to 38620) | 243.06 (128.27 to 382.08) | 1.13 (1.04 to 1.22) |
| Burundi | 5751 (556 to 10006) | 241.56 (23.78 to 415.99) | 10425 (5172 to 15226) | 204.86 (100.92 to 302.88) | -1.06 (-1.28 to -0.84) |
| Cabo Verde | 523 (345 to 736) | 237.73 (157.89 to 333.77) | 1064 (772 to 1427) | 231.29 (166.6 to 312.19) | -0.32 (-0.56 to -0.09) |
| Cambodia | 9228 (4273 to 14476) | 189.06 (89.07 to 298.81) | 23262 (12538 to 34222) | 178.51 (95.73 to 265.98) | -0.22 (-0.35 to -0.09) |
| Cameroon | 18908 (10056 to 25997) | 399.08 (207.46 to 557.69) | 58225 (28234 to 87725) | 422.29 (204.91 to 637.6) | 0.14 (-0.07 to 0.34) |
| Canada | 7124 (5439 to 8794) | 22.46 (17.28 to 27.82) | 26238 (19264 to 32746) | 40.78 (32.4 to 48.52) | 2.38 (1.99 to 2.77) |
| Central African Republic | 5100 (1616 to 8956) | 424.77 (141.46 to 714.9) | 13092 (5018 to 22156) | 560.91 (216.52 to 923.31) | 0.85 (0.8 to 0.91) |
| Chad | 7115 (3225 to 10323) | 247.18 (112.72 to 363.56) | 19834 (9757 to 31365) | 312.25 (151.22 to 476.8) | 0.64 (0.4 to 0.88) |
| Chile | 12638 (9536 to 15691) | 131.05 (93.14 to 167.23) | 29270 (18811 to 37707) | 113.04 (73.79 to 144.73) | -0.08 (-0.3 to 0.15) |
| China | 1393488 (812664 to 1929010) | 182.56 (104.75 to 260.36) | 2373040 (1485461 to 3462005) | 120.03 (71.9 to 180.13) | -1.46 (-1.89 to -1.03) |
| Colombia | 41200 (32173 to 50435) | 243.14 (177.22 to 308.23) | 40959 (27363 to 55516) | 73.42 (49.29 to 99) | -4.34 (-4.6 to -4.08) |
| Comoros | 776 (361 to 1260) | 382.23 (182.03 to 603.73) | 1803 (1043 to 2744) | 369.91 (208.35 to 574.37) | -0.39 (-0.6 to -0.17) |
| Congo | 7133 (3332 to 11161) | 641.02 (297.12 to 994.06) | 18745 (10495 to 28548) | 676.86 (383.18 to 1007.27) | -0.12 (-0.27 to 0.04) |
| Cook Islands | 199 (151 to 252) | 1515.14 (1136.8 to 1925.02) | 213 (161 to 276) | 858.6 (639.22 to 1119.65) | -1.79 (-1.91 to -1.66) |
| Costa Rica | 1933 (1470 to 2406) | 111.47 (82.76 to 141.78) | 5078 (3446 to 6601) | 91.11 (62.22 to 118.03) | -1.66 (-2.03 to -1.3) |
| Croatia | 13130 (8981 to 17450) | 239.15 (157.29 to 327.96) | 11106 (6716 to 15328) | 121.21 (77.57 to 162.83) | -1.35 (-1.78 to -0.91) |
| Cuba | 6409 (5266 to 7662) | 62.73 (51.24 to 75.25) | 32207 (23775 to 40642) | 165.76 (125.45 to 207.12) | 3.65 (3.47 to 3.84) |
| Cyprus | 862 (405 to 1410) | 148.04 (57.98 to 277.17) | 1461 (837 to 2148) | 89.31 (42.91 to 135.43) | -1.76 (-2.02 to -1.5) |
| Czechia | 6706 (5373 to 8002) | 49.72 (39.72 to 59.43) | 21180 (13511 to 28223) | 95.73 (64.02 to 125.74) | 1.93 (1.16 to 2.71) |
| Côte d'Ivoire | 11948 (6872 to 17031) | 263.42 (146.9 to 380.78) | 46070 (25948 to 66259) | 360.6 (204.47 to 520.59) | 1.03 (0.78 to 1.27) |
| Democratic People's Republic of Korea | 16712 (9433 to 26524) | 119.56 (65.15 to 194.38) | 53922 (34411 to 81006) | 177.24 (109.72 to 267.15) | 1.46 (1.37 to 1.56) |
| Democratic Republic of the Congo | 53145 (23562 to 86057) | 336.79 (147.9 to 529.69) | 193107 (106135 to 304851) | 551.64 (301.35 to 855.44) | 1.62 (1.55 to 1.69) |
| Denmark | 1654 (1110 to 2230) | 20.03 (14.08 to 26.41) | 2078 (1149 to 2953) | 16.45 (10.14 to 22.48) | -0.65 (-0.9 to -0.4) |
| Djibouti | 394 (236 to 597) | 265.61 (162.94 to 405.27) | 1578 (980 to 2470) | 237.15 (141.92 to 378.77) | -0.6 (-0.68 to -0.51) |
| Dominica | 351 (256 to 441) | 603.83 (431.62 to 764.14) | 503 (364 to 671) | 611.43 (437.42 to 811.42) | 0.24 (0.04 to 0.43) |
| Dominican Republic | 7025 (5367 to 9207) | 180.87 (132.97 to 245.52) | 21129 (14641 to 29318) | 207.6 (142.35 to 288.03) | 0.92 (0.75 to 1.1) |
| Ecuador | 11227 (8808 to 13944) | 207.77 (154.75 to 266.87) | 20046 (13455 to 28553) | 128.42 (82.05 to 185.69) | -0.12 (-1.01 to 0.78) |
| Egypt | 243840 (179802 to 332338) | 987.4 (649.85 to 1419.28) | 462209 (351183 to 602731) | 806.63 (564.79 to 1086.13) | -0.43 (-0.57 to -0.29) |
| El Salvador | 2312 (1759 to 2940) | 77.65 (57.79 to 100.21) | 3825 (2575 to 5366) | 59.88 (40.61 to 83.52) | -0.88 (-1.13 to -0.62) |
| Equatorial Guinea | 1376 (702 to 2211) | 679.97 (357.64 to 1082.97) | 2645 (1434 to 4272) | 524.48 (272.37 to 864.79) | -1.17 (-1.5 to -0.84) |
| Eritrea | 4035 (1620 to 6344) | 323.37 (145.7 to 498.41) | 8760 (4863 to 13257) | 306.46 (165.12 to 452.8) | -0.2 (-0.33 to -0.07) |
| Estonia | 3595 (2860 to 4286) | 182.32 (146.17 to 217.51) | 18481 (10965 to 25065) | 631.73 (413.55 to 825.35) | 5.88 (4.92 to 6.85) |
| Eswatini | 2072 (1278 to 2759) | 740.46 (458.08 to 1018.61) | 5084 (2629 to 7927) | 906.55 (483.63 to 1372.17) | 1.05 (0.52 to 1.57) |
| Ethiopia | 72524 (33373 to 105388) | 336.22 (171.3 to 486.87) | 76754 (52644 to 110255) | 167.65 (109.45 to 244.03) | -2.88 (-3.1 to -2.66) |
| Fiji | 1766 (1351 to 2279) | 434.73 (327.82 to 564.77) | 2967 (2141 to 4006) | 376.47 (272.84 to 503.69) | -0.67 (-0.8 to -0.55) |
| Finland | 3868 (2661 to 5139) | 54.42 (37.45 to 72.14) | 14387 (7302 to 20370) | 103.06 (61.97 to 137.64) | 3.33 (2.68 to 3.98) |
| France | 36886 (19299 to 55235) | 42.74 (23.91 to 62.48) | 58326 (23943 to 90871) | 32.28 (17.86 to 46.61) | -0.83 (-0.94 to -0.71) |
| Gabon | 3944 (2294 to 5697) | 702.1 (401.54 to 1018.62) | 6903 (4057 to 10505) | 691.05 (395.41 to 1079.35) | -0.23 (-0.41 to -0.05) |
| Gambia | 1105 (699 to 1593) | 288.71 (183.13 to 415.47) | 4670 (2718 to 6835) | 435.11 (247.71 to 638.75) | 1.15 (0.92 to 1.37) |
| Georgia | 9616 (7377 to 12543) | 156.12 (119.2 to 204.47) | 26247 (17349 to 34304) | 434 (309.91 to 555.5) | 5.52 (4.34 to 6.72) |
| Germany | 169391 (108703 to 224682) | 131.46 (89.98 to 170.59) | 198363 (78567 to 294689) | 85.13 (42.5 to 119.89) | -0.25 (-0.6 to 0.09) |
| Ghana | 22560 (13844 to 31536) | 314.69 (195.3 to 453.43) | 66852 (40176 to 92700) | 366.62 (221.46 to 517.49) | 0.21 (-0.03 to 0.45) |
| Greece | 7807 (4683 to 10902) | 53.98 (31.28 to 76.47) | 20969 (8967 to 30952) | 71.42 (41.05 to 97.73) | 1.67 (1.14 to 2.2) |
| Greenland | 63 (49 to 80) | 151.74 (115.33 to 197.85) | 70 (52 to 89) | 99.37 (73.88 to 126.86) | -1.06 (-1.31 to -0.82) |
| Grenada | 192 (148 to 238) | 284.07 (224.11 to 344.96) | 421 (339 to 501) | 371.21 (286.67 to 452.73) | 1.43 (1.1 to 1.75) |
| Guam | 427 (273 to 537) | 529.34 (326.25 to 687.17) | 423 (334 to 614) | 207.24 (164.56 to 300.5) | -2.99 (-3.6 to -2.38) |
| Guatemala | 2625 (2213 to 3087) | 80.06 (60.12 to 98.89) | 4777 (3491 to 6114) | 46.77 (31.47 to 61.39) | -1.28 (-1.79 to -0.76) |
| Guinea | 9316 (4882 to 14213) | 270.08 (142.18 to 407.76) | 20343 (10986 to 30225) | 330.78 (177.17 to 481.93) | 0.65 (0.47 to 0.83) |
| Guinea-Bissau | 1881 (857 to 2891) | 423.62 (199.53 to 651.63) | 4547 (2224 to 6950) | 525.19 (257.42 to 810.74) | 0.67 (0.48 to 0.85) |
| Guyana | 3264 (2609 to 3927) | 829.44 (639.85 to 1034.87) | 5321 (3965 to 7087) | 794.66 (576.88 to 1060.21) | 0.56 (0.16 to 0.96) |
| Haiti | 8733 (3175 to 14819) | 249.46 (91.48 to 427.27) | 23041 (10227 to 37306) | 286.98 (130.74 to 467.14) | 0.6 (0.53 to 0.66) |
| Honduras | 5180 (3924 to 6970) | 248.9 (181.9 to 353.06) | 20049 (14778 to 27202) | 326.55 (235.11 to 450.74) | 1.02 (0.87 to 1.16) |
| Hungary | 45298 (34434 to 55524) | 316.77 (236.73 to 389.9) | 61056 (42677 to 77478) | 310.2 (229.91 to 384.36) | 0.77 (0.31 to 1.24) |
| Iceland | 77 (47 to 105) | 26.06 (16.75 to 35.1) | 161 (85 to 231) | 24.81 (14.56 to 34.72) | 0.71 (0.42 to 0.99) |
| India | 289985 (160729 to 430767) | 60.29 (33.67 to 92.22) | 1106654 (761713 to 1570792) | 93.42 (63.88 to 132.15) | 1.57 (1.49 to 1.66) |
| Indonesia | 162633 (86301 to 233137) | 149.87 (78.02 to 221.57) | 551510 (335103 to 755839) | 216.25 (129.28 to 304.59) | 1.31 (1.15 to 1.46) |
| Iran (Islamic Republic of) | 81781 (61269 to 108317) | 327.6 (236.86 to 453.71) | 225779 (172036 to 277482) | 304.65 (218.62 to 388.26) | 0.15 (-0.02 to 0.31) |
| Iraq | 35579 (20653 to 50515) | 446.7 (258.18 to 630.79) | 81876 (57551 to 110032) | 375.81 (246.43 to 509.09) | -1.06 (-1.21 to -0.91) |
| Ireland | 1010 (673 to 1350) | 25.69 (15.95 to 34.84) | 1455 (800 to 2036) | 17.78 (10.26 to 24.43) | -0.27 (-0.52 to -0.02) |
| Israel | 1981 (1337 to 2684) | 42.12 (26.77 to 57.36) | 2533 (1371 to 3622) | 18.97 (11.24 to 26.23) | -2.52 (-3.25 to -1.77) |
| Italy | 81839 (52235 to 111995) | 91.49 (57.05 to 125.7) | 188764 (74349 to 288894) | 101.33 (50.64 to 144) | 0.41 (0.29 to 0.53) |
| Jamaica | 6604 (5006 to 8462) | 370.14 (284.2 to 466.52) | 12354 (8936 to 16461) | 392.2 (284.14 to 527.44) | 0.96 (0.36 to 1.57) |
| Japan | 64151 (40601 to 91066) | 41.05 (24.36 to 59.93) | 63563 (28398 to 102207) | 15.53 (10.15 to 21.43) | -2.91 (-3.75 to -2.05) |
| Jordan | 8221 (5852 to 11004) | 645.69 (442.13 to 873.01) | 29545 (21782 to 37672) | 441.04 (297.66 to 576.11) | -1.39 (-1.62 to -1.17) |
| Kazakhstan | 16998 (13847 to 20173) | 130.96 (106.95 to 156) | 18664 (14354 to 24394) | 107.38 (79.59 to 140.22) | -1.87 (-3.58 to -0.12) |
| Kenya | 16971 (11497 to 23075) | 198.8 (131.06 to 277.31) | 65298 (43060 to 87438) | 286.37 (175.82 to 398.55) | 1.58 (1.43 to 1.73) |
| Kiribati | 102 (67 to 130) | 240.57 (158.43 to 311.83) | 194 (127 to 264) | 234.11 (154.43 to 313.76) | -0.14 (-0.17 to -0.12) |
| Kuwait | 2939 (2467 to 3442) | 461.9 (355.41 to 570.72) | 5418 (3972 to 7192) | 196.8 (129.64 to 273.79) | -2.65 (-2.99 to -2.31) |
| Kyrgyzstan | 4114 (3216 to 5017) | 137.7 (106.34 to 169.17) | 10216 (8007 to 12828) | 214.81 (164.14 to 273.56) | 1.01 (0.7 to 1.33) |
| Lao People's Democratic Republic | 6041 (2064 to 9943) | 274.51 (98.34 to 453.73) | 12188 (7006 to 17180) | 243.06 (140.67 to 342.51) | -0.36 (-0.4 to -0.33) |
| Latvia | 1545 (1231 to 1832) | 44.5 (35.74 to 52.58) | 8740 (5708 to 11285) | 216.24 (157.87 to 267.1) | 6.79 (5.65 to 7.94) |
| Lebanon | 6252 (2387 to 9908) | 304.81 (117.82 to 493.96) | 8213 (5189 to 11212) | 129.9 (86.09 to 173.96) | -3.07 (-3.29 to -2.86) |
| Lesotho | 4399 (2890 to 6108) | 532.98 (350.15 to 748.32) | 9962 (5291 to 14646) | 932.26 (490.14 to 1361.03) | 2.69 (2.16 to 3.22) |
| Liberia | 4556 (2832 to 6396) | 375.89 (234.69 to 533.42) | 12526 (6883 to 18842) | 503.34 (274.68 to 757.18) | 0.85 (0.7 to 1.01) |
| Libya | 5920 (3345 to 9189) | 310.57 (175.4 to 476.1) | 24362 (13751 to 38589) | 471.02 (260 to 729.2) | 1.69 (1.56 to 1.83) |
| Lithuania | 2137 (1655 to 2598) | 48.11 (37.11 to 58.46) | 7221 (5110 to 9139) | 127.75 (96.09 to 160.59) | 4 (3.28 to 4.73) |
| Luxembourg | 230 (145 to 319) | 43.75 (26.16 to 60.94) | 461 (228 to 664) | 38.61 (21.12 to 54.35) | 0.11 (-0.12 to 0.33) |
| Madagascar | 23418 (14193 to 33365) | 439.07 (268.65 to 637.95) | 71253 (42697 to 104949) | 592.02 (375.91 to 866.64) | 0.87 (0.79 to 0.95) |
| Malawi | 8553 (3646 to 13330) | 212.26 (94.88 to 325.72) | 22162 (11797 to 32596) | 280.95 (150.5 to 410.59) | 0.62 (0.46 to 0.79) |
| Malaysia | 7303 (4874 to 9504) | 74.94 (49.46 to 98.69) | 17352 (13119 to 21580) | 60.16 (44.76 to 76.29) | -1.32 (-1.6 to -1.03) |
| Maldives | 128 (42 to 203) | 124.45 (43.62 to 198.92) | 254 (189 to 342) | 67.43 (48.3 to 93.97) | -2.32 (-2.54 to -2.1) |
| Mali | 11459 (4184 to 17583) | 264.53 (101.44 to 404.49) | 25757 (11385 to 39189) | 255.45 (115.88 to 385.24) | -0.16 (-0.22 to -0.09) |
| Malta | 185 (128 to 245) | 45.49 (29.73 to 61.41) | 453 (248 to 631) | 43.82 (26.81 to 59.08) | 0.62 (0.35 to 0.89) |
| Marshall Islands | 100 (61 to 144) | 567.97 (355.36 to 808.47) | 218 (129 to 322) | 534.88 (322.78 to 768.21) | -0.29 (-0.41 to -0.18) |
| Mauritania | 5272 (3044 to 7794) | 513.5 (294.87 to 760.34) | 10258 (6010 to 16658) | 465.29 (268.67 to 744.76) | -0.49 (-0.58 to -0.39) |
| Mauritius | 2879 (2295 to 3451) | 380.65 (298.86 to 469.08) | 4881 (3903 to 5954) | 275.63 (217.1 to 342.22) | -0.78 (-1.45 to -0.1) |
| Mexico | 42338 (31988 to 53010) | 108.14 (75.04 to 142.49) | 97361 (66873 to 128508) | 80.47 (53.12 to 107.55) | -0.78 (-1.08 to -0.48) |
| Micronesia (Federated States of) | 325 (187 to 462) | 629.33 (359.42 to 889.36) | 444 (278 to 618) | 540.42 (344.13 to 744.75) | -0.55 (-0.58 to -0.51) |
| Monaco | 37 (20 to 59) | 49.9 (30.22 to 77.15) | 64 (31 to 99) | 58 (32.61 to 84.69) | 0.69 (0.28 to 1.1) |
| Mongolia | 1452 (874 to 2124) | 140.02 (84.12 to 206.58) | 2081 (1366 to 3038) | 92.13 (57.69 to 136.41) | -1.85 (-2.11 to -1.6) |
| Montenegro | 577 (392 to 806) | 94.67 (63.42 to 133.17) | 1117 (727 to 1663) | 121.75 (75.15 to 179.32) | 0.92 (0.84 to 1.01) |
| Morocco | 65049 (34161 to 97081) | 459.95 (244.42 to 687.37) | 162264 (92590 to 239538) | 489.6 (286.75 to 725.69) | 0.44 (0.31 to 0.58) |
| Mozambique | 20180 (11622 to 29031) | 328.29 (181.94 to 475.07) | 57205 (32844 to 88435) | 487.2 (276.82 to 751.69) | 1.72 (1.53 to 1.91) |
| Myanmar | 62317 (23291 to 100477) | 247.55 (91.61 to 397.88) | 96446 (49984 to 146391) | 187.96 (95.84 to 286.12) | -1.24 (-1.37 to -1.12) |
| Namibia | 3228 (2250 to 4425) | 509.16 (332.71 to 721.1) | 8288 (5441 to 12055) | 631.26 (398.28 to 920.96) | 0.44 (0.07 to 0.81) |
| Nauru | 36 (17 to 52) | 646.11 (305.64 to 942.13) | 39 (19 to 58) | 581.64 (284.42 to 863.54) | -0.45 (-0.72 to -0.18) |
| Nepal | 7484 (3712 to 11671) | 73.4 (37.09 to 116.24) | 24422 (16471 to 35025) | 102.71 (68.67 to 147.04) | 1.4 (1 to 1.8) |
| Netherlands | 3550 (2343 to 4822) | 17.69 (11.8 to 23.84) | 7544 (3490 to 11293) | 19.59 (10 to 28.49) | 0.8 (0.53 to 1.06) |
| New Zealand | 1559 (1178 to 1927) | 41.07 (30.97 to 51.39) | 1836 (1199 to 2408) | 21.8 (15.27 to 27.72) | -2.48 (-2.88 to -2.08) |
| Nicaragua | 2056 (1567 to 2618) | 135.7 (97.05 to 176.54) | 5342 (3903 to 7224) | 114.12 (80.32 to 155.69) | -0.69 (-0.85 to -0.53) |
| Niger | 6619 (2411 to 10535) | 218.02 (77.5 to 350.87) | 20256 (7135 to 32319) | 226.05 (76.82 to 358.2) | -0.02 (-0.18 to 0.14) |
| Nigeria | 116968 (77724 to 168839) | 261.91 (167.51 to 388.15) | 241907 (131765 to 341700) | 254.16 (146.87 to 359.53) | -0.51 (-0.73 to -0.29) |
| Niue | 8 (6 to 12) | 391.99 (260.55 to 539.92) | 7 (5 to 10) | 347.48 (232.57 to 463.35) | -0.6 (-0.66 to -0.54) |
| North Macedonia | 5847 (3853 to 8001) | 343.38 (213.57 to 483.49) | 10077 (6469 to 14803) | 368 (205.71 to 552.64) | 0.44 (0.11 to 0.77) |
| Northern Mariana Islands | 27 (19 to 38) | 118 (81.21 to 159.46) | 57 (45 to 68) | 103.33 (79.2 to 126.22) | -0.3 (-0.4 to -0.19) |
| Norway | 1394 (869 to 1939) | 19.45 (12.77 to 26.28) | 1532 (720 to 2310) | 13.21 (7.13 to 19.13) | -0.82 (-1.4 to -0.23) |
| Oman | 3192 (2054 to 4768) | 456.92 (293.08 to 717.54) | 9309 (6344 to 12707) | 498.82 (333.96 to 675.71) | 1.11 (0.74 to 1.48) |
| Pakistan | 65694 (38074 to 94840) | 116.96 (65.32 to 170.76) | 227920 (152006 to 326159) | 185.96 (121.14 to 272.14) | 1.4 (1.06 to 1.73) |
| Palau | 16 (12 to 22) | 157.61 (114.96 to 215.65) | 31 (23 to 41) | 133.36 (97.08 to 175.11) | -0.51 (-0.56 to -0.46) |
| Palestine | 4630 (2877 to 6944) | 572.3 (347.31 to 864.12) | 8712 (6235 to 11173) | 403.91 (260.58 to 538.63) | -1.1 (-1.24 to -0.95) |
| Panama | 924 (694 to 1145) | 63.63 (46.78 to 80.27) | 4051 (2756 to 5322) | 90.46 (61.98 to 118.45) | 0.78 (0.28 to 1.28) |
| Papua New Guinea | 4898 (2360 to 7894) | 223.72 (113.55 to 355.53) | 13385 (7187 to 22306) | 207.21 (115.01 to 349.31) | -0.31 (-0.35 to -0.27) |
| Paraguay | 3840 (2864 to 4984) | 171.72 (123.27 to 224.09) | 11406 (7968 to 15647) | 198.2 (136.05 to 273.43) | 0.63 (0.51 to 0.75) |
| Peru | 9950 (7241 to 12933) | 80.85 (57.35 to 105.81) | 18673 (12800 to 26390) | 54.81 (37.35 to 77.92) | -1.44 (-1.73 to -1.15) |
| Philippines | 71260 (54987 to 87457) | 220.1 (167.78 to 280.47) | 281427 (211302 to 355909) | 321.32 (234.1 to 411.39) | 1.4 (1.28 to 1.52) |
| Poland | 63955 (49315 to 77982) | 149.64 (116.13 to 183.83) | 107121 (71758 to 138898) | 148.2 (103.98 to 189.19) | -0.06 (-0.36 to 0.24) |
| Portugal | 6972 (4427 to 9529) | 54.64 (31.76 to 77.05) | 13514 (5504 to 20438) | 45.92 (23.75 to 65.77) | -0.44 (-0.59 to -0.29) |
| Puerto Rico | 6062 (4855 to 7207) | 170.33 (135.6 to 203.81) | 10077 (7150 to 13267) | 148.65 (116.08 to 186.73) | -0.14 (-0.48 to 0.21) |
| Qatar | 293 (216 to 373) | 314.03 (198.67 to 424.03) | 1228 (878 to 1787) | 154.21 (96.21 to 215.63) | -2.52 (-3 to -2.05) |
| Republic of Korea | 21499 (12662 to 29386) | 79.27 (44.68 to 115.41) | 24234 (11110 to 41023) | 27.47 (12.46 to 46.46) | -3.41 (-3.64 to -3.17) |
| Republic of Moldova | 3220 (2658 to 3764) | 72.33 (59.26 to 85.17) | 21735 (16139 to 27288) | 360 (269.91 to 450.7) | 5.92 (5.5 to 6.34) |
| Romania | 105141 (80450 to 130040) | 394.62 (285.14 to 501.63) | 135027 (84590 to 177964) | 350.94 (237.75 to 451.91) | 0.25 (-0.11 to 0.61) |
| Russian Federation | 97220 (81579 to 110769) | 54.3 (45.44 to 62.08) | 183316 (139860 to 222963) | 78.14 (60.34 to 94.07) | 0.76 (-0.51 to 2.05) |
| Rwanda | 13149 (2821 to 20605) | 433.38 (100.37 to 680.74) | 15669 (4283 to 23506) | 251.39 (70.11 to 394.66) | -2.8 (-3.21 to -2.39) |
| Saint Kitts and Nevis | 107 (82 to 132) | 301.77 (233.9 to 371.29) | 228 (174 to 288) | 338.6 (247.25 to 438.36) | 1.3 (0.79 to 1.82) |
| Saint Lucia | 305 (240 to 379) | 365.7 (277.28 to 464.93) | 768 (573 to 999) | 322.64 (236.46 to 418.56) | -0.1 (-0.59 to 0.41) |
| Saint Vincent and the Grenadines | 249 (191 to 313) | 354.94 (267.59 to 449.44) | 772 (605 to 967) | 558.16 (425.97 to 705.61) | 1.55 (1.01 to 2.1) |
| Samoa | 443 (291 to 585) | 493.82 (328.02 to 650.99) | 640 (427 to 846) | 420.62 (280.04 to 553.5) | -0.54 (-0.69 to -0.4) |
| San Marino | 22 (12 to 34) | 60.06 (32.76 to 92.04) | 38 (17 to 61) | 41 (21.78 to 63.73) | -0.12 (-0.47 to 0.23) |
| Sao Tome and Principe | 122 (91 to 156) | 187.38 (137.4 to 242.41) | 259 (184 to 358) | 215.3 (152.15 to 294.08) | 0.23 (0.05 to 0.42) |
| Saudi Arabia | 37962 (22400 to 57395) | 650.11 (380 to 993.4) | 135582 (73388 to 208492) | 661.34 (359.51 to 975.85) | -0.08 (-0.21 to 0.06) |
| Senegal | 9346 (6093 to 13105) | 269.61 (177.71 to 376.53) | 27541 (17142 to 38647) | 332.99 (203.21 to 469.65) | 0.72 (0.61 to 0.82) |
| Serbia | 26104 (17492 to 37448) | 282.04 (172.23 to 413.72) | 39870 (23996 to 55792) | 234.09 (143.24 to 325.33) | -0.8 (-0.96 to -0.65) |
| Seychelles | 444 (338 to 541) | 785.9 (600.07 to 959.33) | 690 (546 to 877) | 594.82 (454.14 to 769.85) | -0.88 (-1.04 to -0.71) |
| Sierra Leone | 4426 (2700 to 6382) | 209.85 (125.03 to 301.85) | 12045 (6280 to 17729) | 288.83 (156.27 to 428) | 1.16 (0.9 to 1.41) |
| Singapore | 2140 (1674 to 2695) | 90.65 (67.37 to 117.7) | 5397 (4264 to 6642) | 62.61 (49.04 to 77.74) | -0.9 (-1.3 to -0.5) |
| Slovakia | 8112 (5686 to 11810) | 137.44 (95.03 to 201.5) | 12556 (8126 to 17695) | 132.58 (86.26 to 186.5) | 0.36 (0.09 to 0.64) |
| Slovenia | 4211 (3069 to 5231) | 171.39 (125.12 to 214.4) | 7139 (3096 to 10561) | 135.55 (69.7 to 193.57) | -0.24 (-0.5 to 0.03) |
| Solomon Islands | 430 (192 to 690) | 274.25 (135.06 to 423.29) | 1163 (693 to 1775) | 282.24 (168.71 to 424.63) | 0.13 (0.07 to 0.18) |
| Somalia | 11744 (4899 to 18856) | 406.58 (185.25 to 640.04) | 25322 (10975 to 41439) | 368.41 (166.02 to 567.92) | -0.39 (-0.52 to -0.25) |
| South Africa | 93260 (74130 to 118417) | 425.86 (326.62 to 555.64) | 259860 (206910 to 321624) | 569.58 (422.84 to 712.96) | 1.02 (0.56 to 1.48) |
| South Sudan | 5645 (2583 to 9093) | 215.46 (100.97 to 347.61) | 8256 (3546 to 12986) | 197.97 (88.18 to 301.63) | -0.5 (-0.88 to -0.12) |
| Spain | 21385 (12827 to 29590) | 40.25 (23.24 to 56.17) | 62243 (25398 to 92011) | 49.46 (25.76 to 69.01) | 0.81 (0.67 to 0.95) |
| Sri Lanka | 20226 (14714 to 26263) | 181.32 (130.05 to 241.48) | 35028 (20667 to 59217) | 130.5 (77.75 to 221.27) | -1.33 (-1.55 to -1.11) |
| Sudan | 57712 (28531 to 88332) | 617.32 (319.73 to 923.89) | 114245 (63212 to 172715) | 581.26 (333.71 to 885.15) | -0.17 (-0.31 to -0.04) |
| Suriname | 629 (483 to 798) | 238.46 (179.72 to 307.23) | 1546 (1102 to 2100) | 240.71 (171.15 to 326.63) | 0.23 (0.06 to 0.41) |
| Sweden | 2271 (1276 to 3279) | 14.12 (8.56 to 19.74) | 10978 (4594 to 16746) | 41.57 (19.93 to 60.42) | 4.99 (4.42 to 5.56) |
| Switzerland | 5985 (3326 to 8748) | 54.92 (32.75 to 78.34) | 12449 (5146 to 19791) | 54.32 (26.1 to 81.52) | 0.46 (0.12 to 0.79) |
| Syrian Arab Republic | 23821 (16051 to 31947) | 468.53 (308.28 to 630.82) | 50018 (33644 to 69783) | 429.61 (262.54 to 614.01) | -0.77 (-1.1 to -0.45) |
| Taiwan (Province of China) | 20116 (15277 to 24972) | 136.32 (98.16 to 180.79) | 41594 (27876 to 54340) | 99.27 (70.22 to 127.78) | 0.35 (-0.52 to 1.23) |
| Tajikistan | 11726 (7767 to 17202) | 439.07 (281.04 to 667.89) | 18501 (12150 to 26286) | 340.78 (219.36 to 484) | -1.07 (-1.43 to -0.72) |
| Thailand | 10253 (6761 to 14176) | 27.14 (17.74 to 37.87) | 33580 (24021 to 46615) | 31.81 (22.91 to 44.35) | 0.12 (-0.06 to 0.29) |
| Timor-Leste | 356 (156 to 573) | 113.72 (49.79 to 187.24) | 1257 (779 to 1911) | 141.89 (88.63 to 212.25) | 0.87 (0.58 to 1.16) |
| Togo | 3861 (2361 to 5619) | 280.41 (170.76 to 413.36) | 16526 (8557 to 25166) | 395.4 (211.24 to 602.52) | 1.15 (0.96 to 1.34) |
| Tokelau | 6 (4 to 9) | 467.94 (309.28 to 654.69) | 5 (3 to 7) | 322.83 (214.51 to 453.87) | -1.32 (-1.36 to -1.28) |
| Tonga | 74 (53 to 99) | 126.53 (89.67 to 168.94) | 100 (71 to 133) | 122.06 (87.26 to 162.51) | -0.15 (-0.29 to -0.01) |
| Trinidad and Tobago | 3275 (2672 to 3899) | 398.18 (308.59 to 493.52) | 4817 (3580 to 6373) | 256.31 (189.83 to 340.45) | -1.68 (-1.95 to -1.41) |
| Tunisia | 16380 (10974 to 22966) | 343.37 (226.14 to 492.06) | 48995 (24831 to 87022) | 385.35 (194.06 to 684.24) | 0.43 (0.37 to 0.48) |
| Turkmenistan | 3780 (2610 to 5031) | 196.34 (132.24 to 268.35) | 10120 (6743 to 14958) | 249.52 (162.64 to 374.88) | 0.5 (0.25 to 0.74) |
| Tuvalu | 43 (25 to 60) | 601.11 (352.28 to 835.25) | 47 (32 to 63) | 437.9 (297.01 to 591.49) | -0.99 (-1.06 to -0.92) |
| Turkey | 107446 (65406 to 152464) | 345.2 (202 to 500.01) | 209593 (132729 to 288111) | 241.8 (145.2 to 339.3) | -1.06 (-1.43 to -0.69) |
| Uganda | 12567 (4004 to 21083) | 188.89 (60.26 to 307.97) | 32106 (13248 to 51545) | 208.77 (83.95 to 334.95) | -0.23 (-0.45 to -0.01) |
| Ukraine | 52564 (41992 to 63286) | 74.42 (59.1 to 89.52) | 70685 (48194 to 97016) | 94.41 (64.64 to 128.57) | 0.52 (0.26 to 0.79) |
| United Arab Emirates | 1666 (1160 to 2431) | 364.92 (233 to 531.8) | 11290 (8289 to 15158) | 369.47 (251.2 to 491.63) | 1.57 (1.02 to 2.13) |
| United Kingdom | 30389 (22954 to 37608) | 35.16 (27.42 to 42.66) | 47226 (33276 to 59587) | 38.24 (29.47 to 46.21) | 0.86 (0.7 to 1.02) |
| United Republic of Tanzania | 36186 (16351 to 52874) | 337.26 (154.51 to 504.2) | 87628 (38264 to 131386) | 353.84 (153.42 to 540.51) | 0.13 (0.07 to 0.18) |
| United States of America | 311355 (248534 to 373661) | 103.83 (84.32 to 122.7) | 957210 (758537 to 1146595) | 187.98 (155.9 to 219.82) | 2.34 (2.2 to 2.48) |
| United States Virgin Islands | 297 (230 to 386) | 353.58 (262.54 to 470.06) | 336 (229 to 467) | 207.53 (142.96 to 286.96) | -1.32 (-1.47 to -1.17) |
| Uruguay | 3451 (2559 to 4322) | 88.86 (66.18 to 110.88) | 6577 (3978 to 8855) | 110.7 (76.54 to 141.88) | 0.67 (0.52 to 0.83) |
| Uzbekistan | 19396 (13075 to 27193) | 167.48 (112.61 to 235.82) | 63158 (43476 to 88022) | 241.96 (167.97 to 334.49) | 1.38 (1.13 to 1.63) |
| Vanuatu | 214 (129 to 325) | 298.93 (181.68 to 446.23) | 656 (455 to 889) | 325.67 (220.35 to 440.92) | 0.19 (0.12 to 0.27) |
| Venezuela (Bolivarian Republic of) | 32345 (26223 to 38694) | 334.77 (256.4 to 411.6) | 66935 (47228 to 94252) | 228.02 (157.99 to 323.83) | -1.65 (-2 to -1.29) |
| Viet Nam | 35017 (20916 to 56529) | 87.59 (53.07 to 141.9) | 100161 (56888 to 161294) | 100.48 (57.12 to 158.84) | 0.83 (0.56 to 1.09) |
| Yemen | 27134 (13112 to 42124) | 552.43 (270.79 to 848.11) | 89228 (45625 to 148664) | 643.38 (336.39 to 1081.3) | 0.32 (0.16 to 0.48) |
| Zambia | 12898 (7525 to 17940) | 421.59 (248.32 to 586.38) | 36650 (18576 to 53881) | 512.88 (267.16 to 749.03) | 0.31 (0.17 to 0.45) |
| Zimbabwe | 10573 (7520 to 14323) | 261.99 (183.01 to 371.58) | 38534 (24148 to 57153) | 540.18 (338.44 to 796.76) | 3.13 (2.53 to 3.73) |
