# Supplementary material for: Global burden of hypertensive heart disease attributable to high body mass index from 1990 to 2021: a multidimensional analysis and public health response
Source: Front Cardiovasc Med. 2025 Aug 12;12:1570390. doi: 10.3389/fcvm.2025.1570390 (PMC12379062; doi:10.3389/fcvm.2025.1570390)
Supplement: Supplementary file 6 [file Datasheet1.pdf]

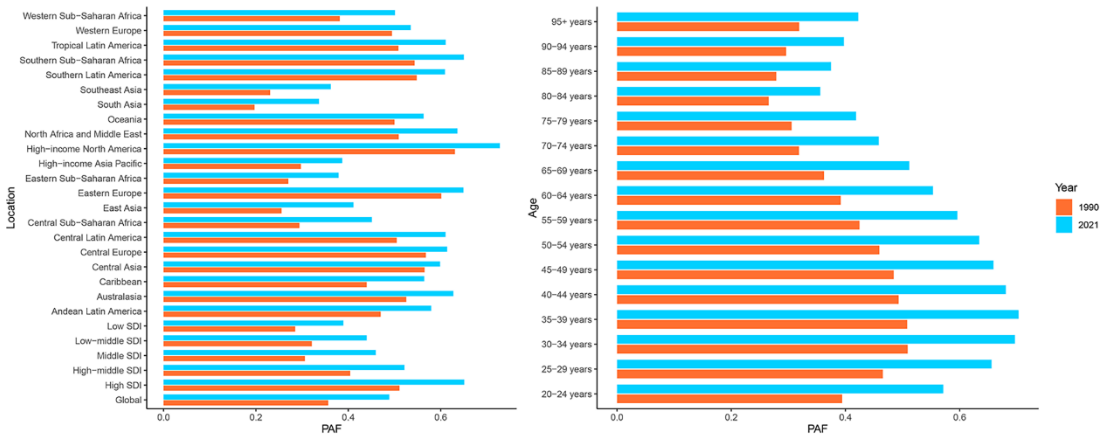


Supplementary Figure S1 Changes in the PAF of DALYs for hypertensive heart disease attributable to high BMI from 1990 to 2021.
