# Supplementary material for: Global burden of hypertensive heart disease attributable to high body mass index from 1990 to 2021: a multidimensional analysis and public health response
Source: Front Cardiovasc Med. 2025 Aug 12;12:1570390. doi: 10.3389/fcvm.2025.1570390 (PMC12379062; doi:10.3389/fcvm.2025.1570390)
Supplement: Supplementary file 9 [file Datasheet4.pdf]

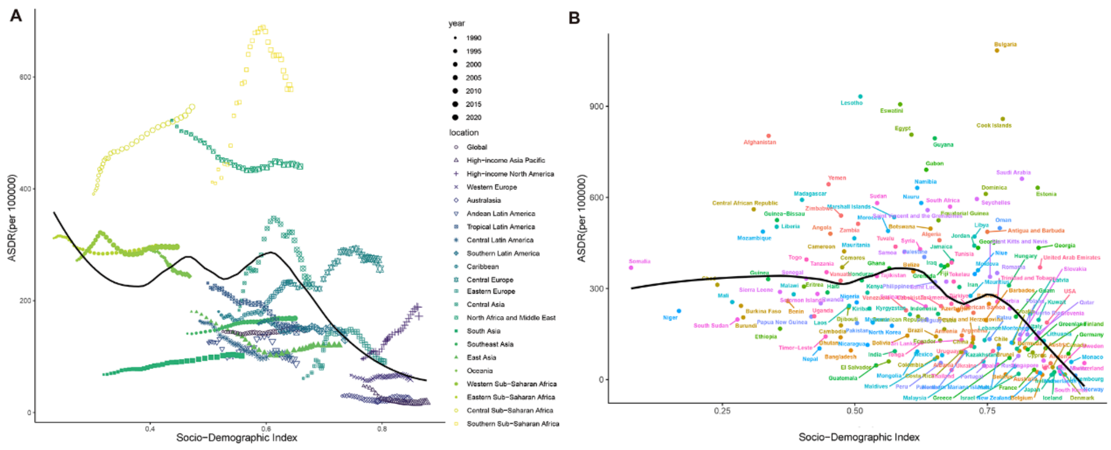

Supplementary Figure S4 Correlation between ASDR of high BMI-related hypertensive heart disease and SDI from 1990 to 2021, stratified by region or country: (A) 21 GBD regions, (B) 204 countries.
