# Supplementary material for: Global burden of hypertensive heart disease attributable to high body mass index from 1990 to 2021: a multidimensional analysis and public health response
Source: Front Cardiovasc Med. 2025 Aug 12;12:1570390. doi: 10.3389/fcvm.2025.1570390 (PMC12379062; doi:10.3389/fcvm.2025.1570390)
Supplement: Supplementary file 10 [file Datasheet5.pdf]

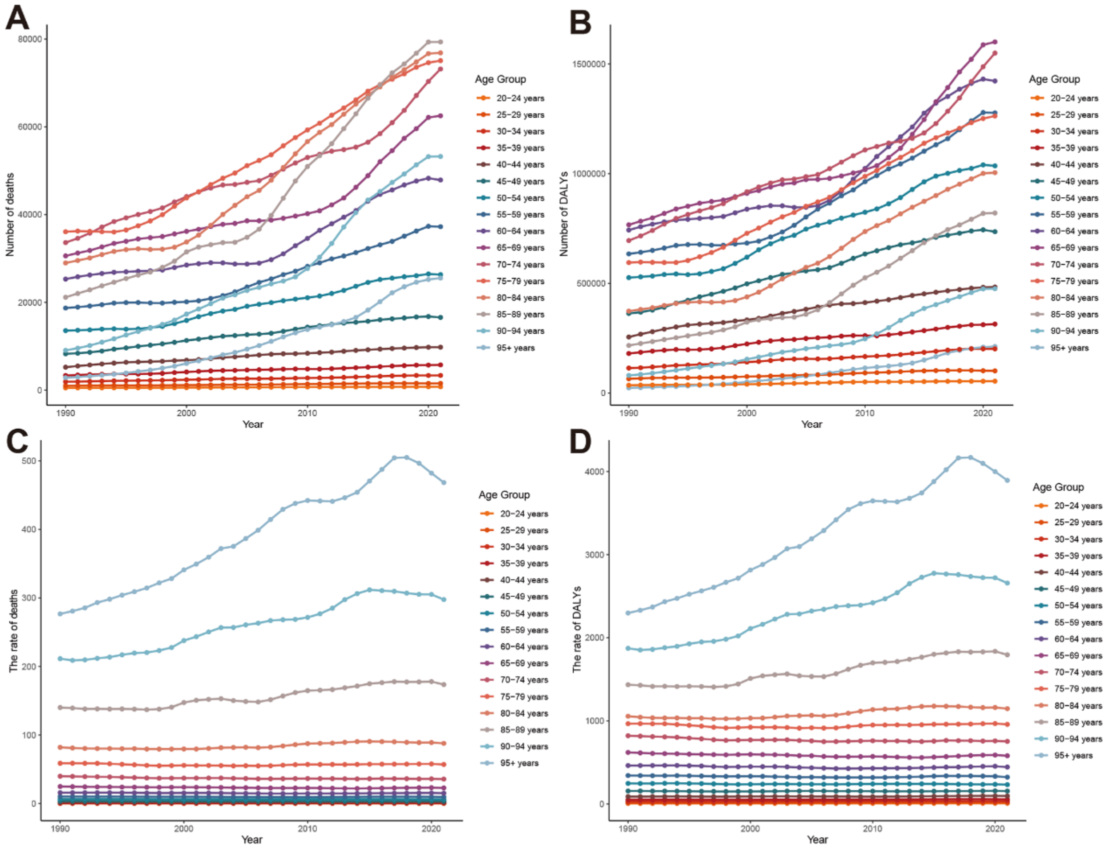

Supplementary Figure S5 Trends in the global burden of high BMI-related HHD from 1990 to 2021, stratified by age group: (A) Deaths, (B) DALYs, (C) Mortality rate, (D) DALY rate.
