# Supplementary material for: Global burden of hypertensive heart disease attributable to high body mass index from 1990 to 2021: a multidimensional analysis and public health response
Source: Front Cardiovasc Med. 2025 Aug 12;12:1570390. doi: 10.3389/fcvm.2025.1570390 (PMC12379062; doi:10.3389/fcvm.2025.1570390)
Supplement: Supplementary file 11 [file Datasheet6.pdf]

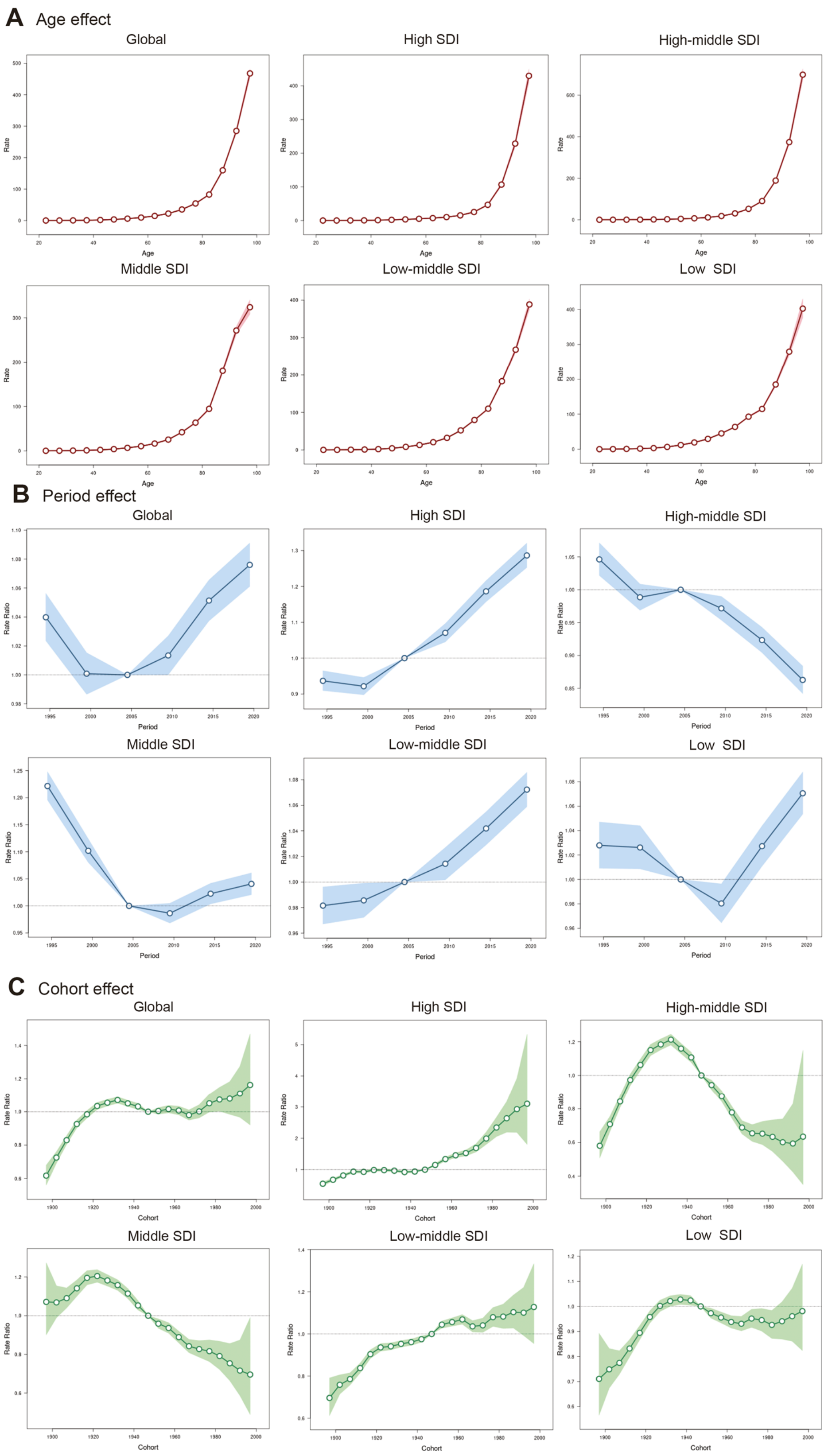

Supplementary Figure S6 Age-period-cohort effects on mortality from high BMI-related hypertensive heart disease from 1990 to 2021, stratified by SDI level: (A) Age effect, (B) Period effect, (C) Cohort effect.
