# Supplementary material for: Global burden of hypertensive heart disease attributable to high body mass index from 1990 to 2021: a multidimensional analysis and public health response
Source: Front Cardiovasc Med. 2025 Aug 12;12:1570390. doi: 10.3389/fcvm.2025.1570390 (PMC12379062; doi:10.3389/fcvm.2025.1570390)
Supplement: Supplementary file 13 [file Datasheet8.pdf]

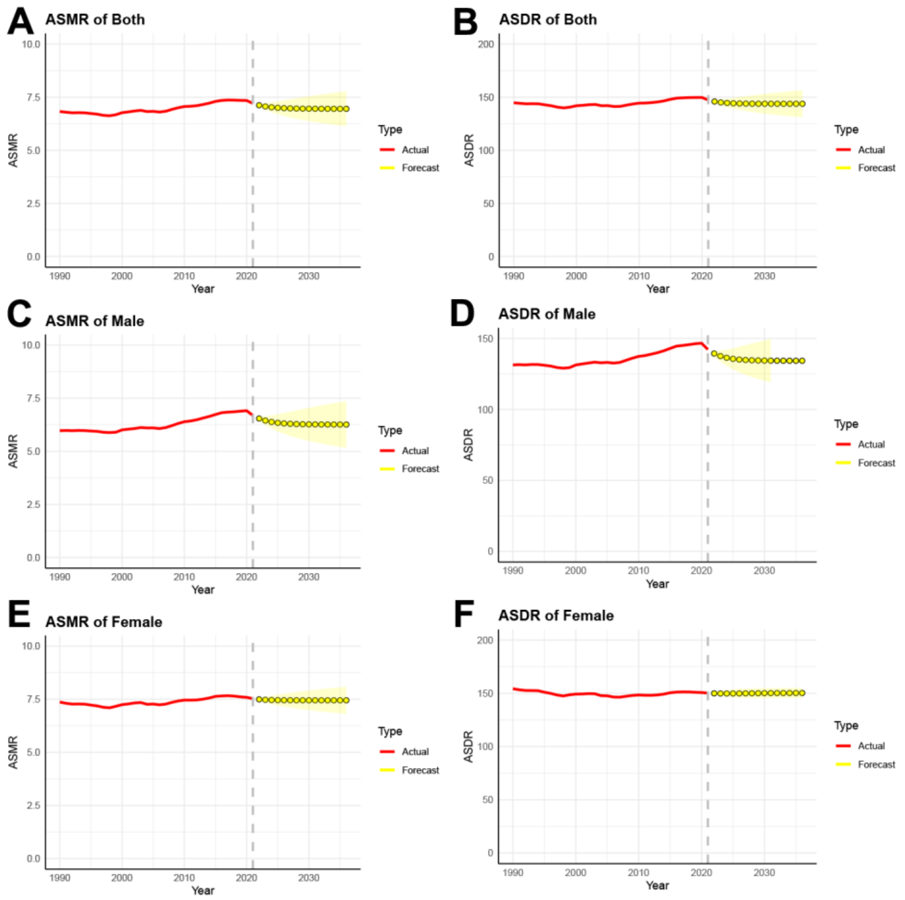

Supplementary Figure S8 Trends in ASMR and ASDR for high BMI-related HHD from 1990 to 2036, observed and predicted using the ARIMA model by sex: (A) Both ASMR, (B) Both ASDR, (C) Male ASMR, (D) Male ASDR, (E) Female ASMR, (F) Female ASDR.
