# Supplementary material for: Global burden of hypertensive heart disease attributable to high body mass index from 1990 to 2021: a multidimensional analysis and public health response
Source: Front Cardiovasc Med. 2025 Aug 12;12:1570390. doi: 10.3389/fcvm.2025.1570390 (PMC12379062; doi:10.3389/fcvm.2025.1570390)
Supplement: Supplementary file 14 [file Datasheet9.pdf]

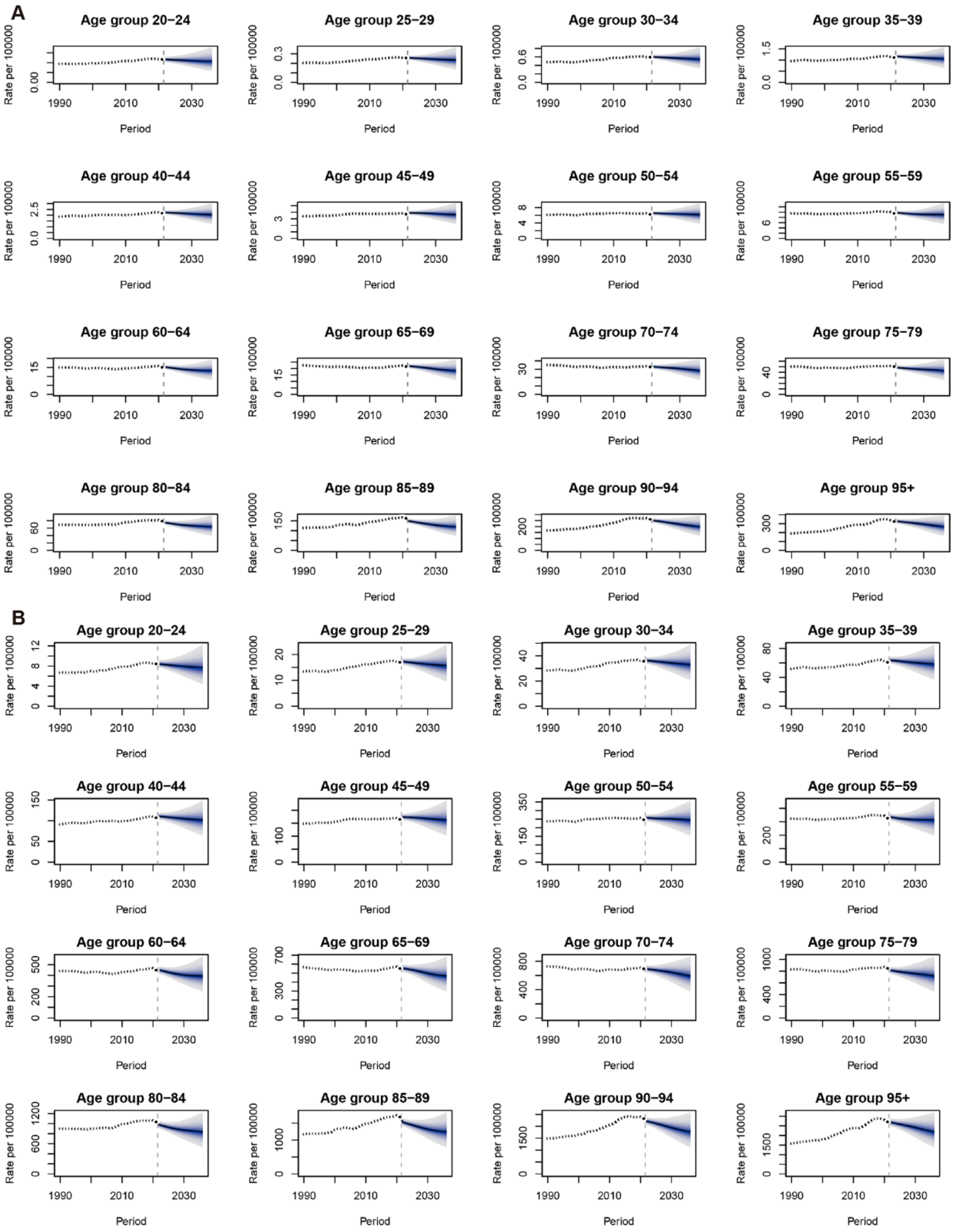

Supplementary Figure S9 Observed and predicted age-stratified trends in deaths and DALYs in men with high BMI-related hypertensive heart disease from 1990 to 2036 using the BAPC model: (A) Mortality rate, (B) DALYs rate.
